# Cancer cells differentially modulate mitochondrial respiration to alter redox state and enable biomass synthesis in nutrient-limited environments

**DOI:** 10.7554/eLife.107123

**Published:** 2026-11-04

**Authors:** Sarah M Chang, Muhammad Bin Munim, Sonia E Trojan, Huel Cox, Anna Shevzov-Zebrun, Keene L Abbott, Renee Chang, Matthew G Vander Heiden

**Affiliations:** 1 Koch Institute for Integrative Cancer Research, Massachusetts Institute of Technology Cambridge United States; 2 Department of Biology, Massachusetts Institute of Technology Cambridge United States; 3 Harvard/MIT MD-PhD Program Boston United States; 4 Jagiellonian University Medical College, Faculty of Medicine, Chair of Medical Biochemistry Krakow Poland; 5 Dana-Farber Cancer Institute Boston United States; The Lowy Medical Research Institute United States; University of Oxford United Kingdom

**Keywords:** NAD+/NADH ratio, mitochondria, serine, cancer metabolism, lipid depletion, Human

## Abstract

The cell NAD+/NADH ratio can constrain biomass synthesis and influence proliferation in nutrient-limited environments. However, which cell processes regulate the NAD+/NADH ratio is not known. Here, we find that some cancer cells elevate the NAD+/NADH ratio in response to serine deprivation by increasing mitochondrial respiration. Cancer cells that elevate mitochondrial respiration have higher serine production and proliferation in serine-limiting conditions than cells with no mitochondrial respiration response, independent of serine synthesis enzyme expression. Increases in mitochondrial respiration and the NAD+/NADH ratio promote serine synthesis regardless of whether serine is environmentally limiting. Lipid deprivation can increase the NAD+/NADH ratio via mitochondrial respiration in some cells, including cells that do not increase respiration following serine deprivation. Thus, in cancer cells where lipid depletion raises the NAD+/NADH ratio, proliferation in serine-depleted environments improves when lipids are also depleted. Taken together, these data suggest that changes in mitochondrial respiration in response to nutrient deprivation can influence the NAD+/NADH ratio in a cell-specific manner to impact oxidative biomass synthesis and proliferation. Given the complexity of tumor microenvironments, this work provides a metabolic framework for understanding how levels of more than one environmental nutrient affect cancer cell proliferation.

## Introduction

Rapidly proliferating cells, including cancer cells, must acquire biomass precursors such as amino acids, lipids, and nucleotides to support cell growth and division (DeBerardinis et al., 2008; Faubert et al., 2020; Hanahan and Weinberg, 2011; Pavlova and Thompson, 2016; Vander Heiden and DeBerardinis, 2017). While cells can obtain biomass precursors from the environment, many tissue and tumor environments can be deficient in necessary nutrients (Cognet and Muir, 2024; Gullino et al., 1964; Lyssiotis and Kimmelman, 2017). In these conditions, cells depend on synthesis pathways to accumulate sufficient biomass for proliferation (Apiz Saab et al., 2023; Ferraro et al., 2021; Ngo et al., 2020; Sciacovelli et al., 2022; Sullivan et al., 2019b; Sullivan et al., 2019a). Synthesis of many biomass precursors involves oxidation reactions that require the redox cofactor NAD+ to act as an electron acceptor. There is accumulating evidence that NAD+ availability and the cell redox state (NAD+/NADH ratio) can limit the synthesis of biomass precursors, including aspartate (Birsoy et al., 2015; Sullivan et al., 2015), asparagine (Krall et al., 2021), fatty acids (Li et al., 2022), serine (Baksh et al., 2020; Bao et al., 2016; Broeks et al., 2023; Diehl et al., 2019), and nucleotides (Bao et al., 2016; Diehl et al., 2019; Wu et al., 2024). Thus, the NAD+/NADH ratio impacts cancer cell proliferation in nutrient environments that increase dependence on biosynthetic oxidation reactions. However, what determines the cell NAD+/NADH ratio and whether the NAD+/NADH ratio is modulated in response to environmental nutrient availability is not well characterized. Moreover, whether differences in the NAD+/NADH ratio across cell types affect sensitivity to different nutrient limitations is not known.

Some tumor microenvironments have low levels of serine, an amino acid used to produce many biomass components, including proteins, nucleotides, glutathione, and lipids. Thus, low serine environments can limit cancer cell proliferation and tumor growth (Banh et al., 2020; Maddocks et al., 2013; Maddocks et al., 2017; Ngo et al., 2020; Sullivan et al., 2019b). In serine-depleted environments, cancer cells must increase serine synthesis to maintain proliferation (DeNicola et al., 2015; Labuschagne et al., 2014; Locasale et al., 2011; Possemato et al., 2011; Sullivan et al., 2019b). Serine synthesis involves the conversion of the glycolytic intermediate 3-phosphoglycerate (3PG) into serine. The production of 3PG from glucose involves an oxidation reaction catalyzed by glyceraldehyde 3-phosphate dehydrogenase (GADPH). 3PG is then oxidized by phosphoglycerate dehydrogenase (PHGDH) in the first step of the serine synthesis pathway. Both GADPH and PHGDH require NAD+ as an electron acceptor to enable serine synthesis. Experimentally blunting NAD+ regeneration from NADH reduces serine synthesis and is rescued by oxidizing the NAD+/NADH ratio via exogenous electron acceptor supplementation (Bao et al., 2016; Diehl et al., 2019; Gravel et al., 2014). Similarly, de novo fatty acid synthesis can be constrained by the NAD+/NADH ratio, as the production of fatty acids involves multiple oxidation reactions to generate citrate from either glucose or glutamine. Thus, decreasing the NAD+/NADH ratio sensitizes cells to environmental lipid depletion (Li et al., 2022). Together, these findings demonstrate that exogenous changes to the NAD+/NADH ratio can alter serine and citrate production and impact proliferation in serine- or lipid-depleted environments. However, auxotrophy for a single nutrient is not sufficient to determine whether cancer cells can form tumors in a tissue, where levels of variable nutrients may be environmentally limiting for proliferation (Abbott et al., 2026; Sivanand et al., 2024; Sullivan et al., 2019a). Thus, we aimed to uncover how the endogenous NAD+/NADH ratio of cancer cells is regulated to influence biosynthesis in different nutrient-depleted environments and its role in determining cancer cell proliferation when multiple oxidized nutrients are limiting.

By culturing cancer cells in serine-depleted conditions and examining the relationship between the NAD+/NADH ratio and serine synthesis, we find that the NAD+/NADH ratio is elevated in response to serine deprivation in some but not all cancer cells. The elevated NAD+/NADH ratio is driven by increased mitochondrial respiration and promotes serine synthesis to confer resistance to environmental serine depletion. A similar relationship between mitochondrial respiration and the NAD+/NADH ratio was also observed in lipid-depleted environments, where higher mitochondrial respiration in some cells led to elevated NAD+/NADH ratios and enhanced citrate synthesis. Lastly, we find that any perturbation that increases the NAD+/NADH ratio, including lipid deprivation, could paradoxically improve the proliferation of cells in serine-depleted conditions. Together, these data reveal that the NAD+/NADH ratio is regulated by mitochondrial respiration in response to environmental nutrients in a cancer cell-specific manner and that this influences oxidative biosynthesis and proliferation. These data also provide insight into how levels of multiple nutrients in tissue microenvironments may cooperate to enable biomass synthesis and cell proliferation.

## Results

### Modulating the cell NAD+/NADH ratio proportionally alters serine synthesis

**Figure 1.**
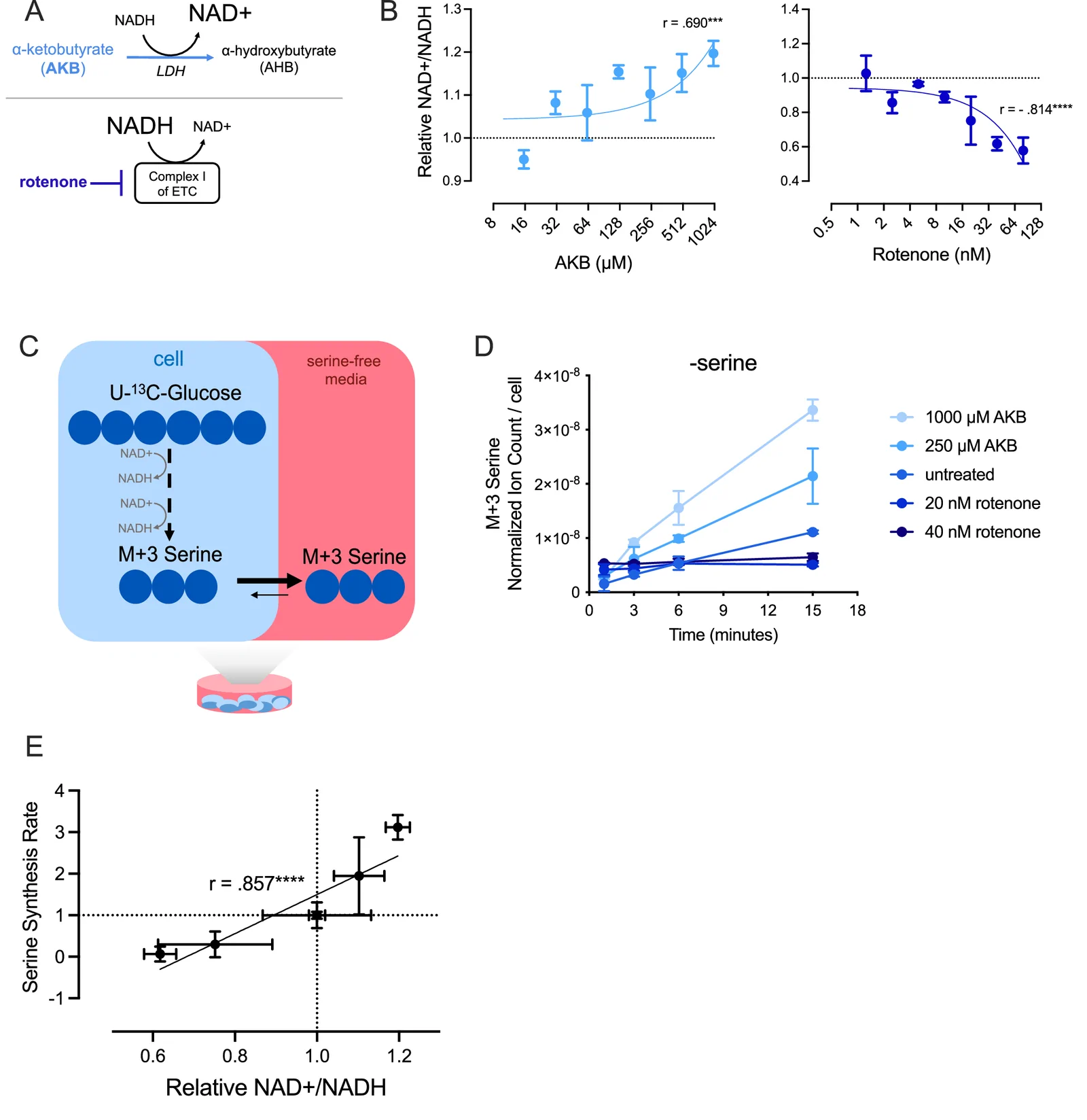
The NAD+/NADH ratio is proportional to serine synthesis rate. (**A**) α-Ketobutyrate (AKB) is an exogenous electron acceptor that promotes the oxidation of NADH into NAD+ through its conversion to α-hydroxybutyrate (AHB). Rotenone inhibits activity of complex I of the mitochondrial electron transport chain (ETC) to block NADH oxidation into NAD+. (**B**) NAD+/NADH ratio measured in A549 cells cultured without serine and indicated concentrations of AKB (left) and rotenone (right) for 24 hr. NAD+/NADH ratios are normalized to untreated cells cultured without serine, n=3. Pearson correlation coefficients and p-values were calculated by simple linear regression, ***p<0.005, ****p<0.001. (**C**) Schematic depicting NAD+ requirement to produce serine labeled (M+3) from U-^13^C-glucose. Under culture conditions without serine, intracellular serine rapidly effluxes from cells into culture media. Thus, cells and media were jointly analyzed at each time point of kinetic tracing to better capture levels of newly synthesized serine. (**D**) Serine labeled (M+3) from U-^13^C-glucose over time in A549 cells cultured without serine and with indicated concentrations of AKB or rotenone for 24 hr prior to U-^13^C-glucose exposure. Serine levels are normalized to internal norvaline standard and cell number measured in indicated conditions, n=3. (**E**) Relative NAD+/NADH ratios from A549 cells cultured without serine after exposure to 250 μM AKB, 1000 μM AKB, 20 nM rotenone, or 40 nM rotenone for 24 hr are plotted against the serine synthesis rates from corresponding conditions as shown in (**D**). Serine synthesis rates are calculated from labeled serine (M+3) after 1 and 15 min of U-^13^C-glucose exposure and normalized to untreated culture conditions without serine, n=3. Pearson correlation coefficients and p-values were calculated by simple linear regression, ****p<0.001. Data shown for all panels represent mean ± SD. Figure 1—source data 1.Excel file for the data in Figure 1B, D and E.

To begin investigating the processes that determine the cell NAD+/NADH ratio and influence biomass synthesis, we examined the relationship between the NAD+/NADH ratio and serine synthesis in serine-replete conditions (400 μM serine, the concentration of serine in DMEM) and serine-depleted conditions (DMEM media formulated without serine). Previous studies have found that decreasing the cell NAD+/NADH ratio (more reduced) can suppress serine production (Baksh et al., 2020; Bao et al., 2016; Broeks et al., 2023; Diehl et al., 2019). To confirm this, we varied the cell NAD+/NADH ratio upon serine withdrawal and measured the serine synthesis rate of A549 non-small cell lung cancer cells, which transcriptionally upregulate the serine synthesis enzyme PHGDH (DeNicola et al., 2015). We increased the NAD+/NADH ratio by treating cells with the exogenous electron acceptor alpha-ketobutyrate (AKB), which can be reduced to alpha-hydroxybutyrate to regenerate NAD+ (Sullivan et al., 2015; Figure 1A). We decreased the NAD+/NADH ratio by treating cells with rotenone, an inhibitor of mitochondrial complex I, which regenerates NAD+ in support of respiration. AKB and rotenone dose-dependently increased and decreased the cell NAD+/NADH ratio, respectively (Figure 1B). To measure the serine synthesis rate in cells, we performed kinetic isotope tracing using uniformly ^13^C-labeled glucose (U-^13^C-glucose) and measured production of M+3-labeled serine (Figure 1C). Because intracellular serine, including newly synthesized serine, rapidly exchanges with the extracellular environment (Labuschagne et al., 2014), we collected both media and cells to help improve the detection of newly synthesized serine produced from glucose. We find that increasing the cell NAD+/NADH ratio with AKB led to proportionally higher serine synthesis rates, whereas lowering the NAD+/NADH ratio with rotenone led to proportionally lower serine synthesis rates (Figure 1D). This was not explained by changes in PHGDH protein expression, as altering the NAD+/NADH ratio with AKB or rotenone did not affect PHGDH protein levels despite modulating serine synthesis (Figure 1—figure supplement 1A and B). Additionally, neither rotenone treatment nor serine deprivation had a large effect on cell viability (Figure 1—figure supplement 1C). Of note, we find that serine synthesis rate is positively correlated with the cell NAD+/NADH ratio (Figure 1E). Together, these data demonstrate that modulating the NAD+/NADH ratio leads to proportional changes in serine synthesis rates.

**Figure 1—figure supplement 1.**
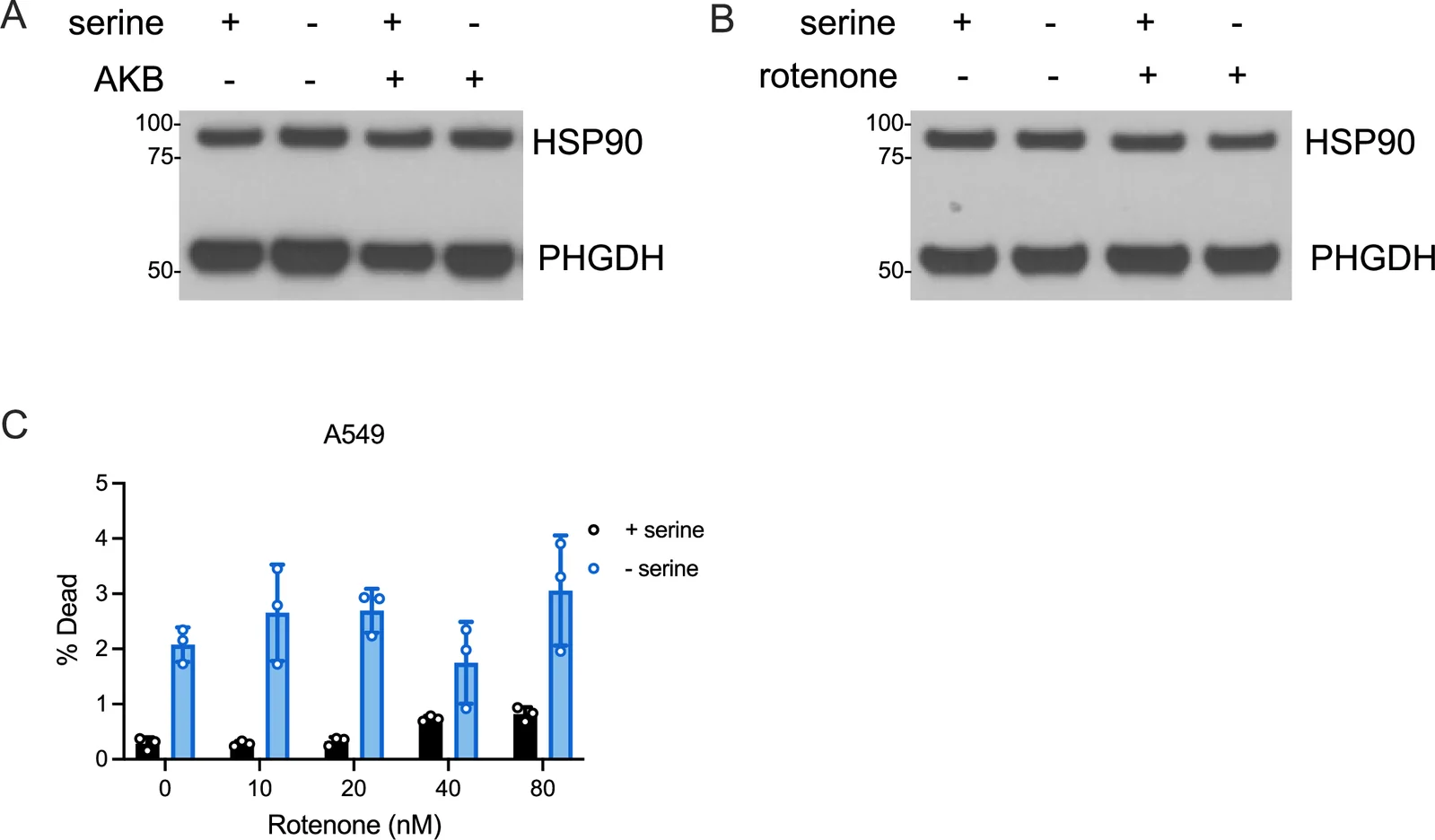
Altering NAD+/NADH with α-ketobutyrate (AKB) or rotenone does not change PHGDH protein levels and minimally changes cell viability. (**A, B**) Immunoblots assessing PHGDH protein levels in A549 cells cultured with or without serine, 1 mM AKB (**A**), or 20 nM rotenone (**B**), as indicated for 24 hr. HSP90 expression is shown as a loading control. (**C**) Percent of A549 cells dead, measured using dye exclusion 72 hr after exposure to the indicated concentrations of rotenone in serine-replete or serine-depleted conditions, n=3. Serine depletion led to a statistically significant increase in cell death compared to serine-replete conditions at all concentrations of rotenone with the exception of 40 nM rotenone, p<0.05. There was no statistical difference in cell death between 0 and 20 nM rotenone in either serine-replete or serine-depleted conditions. p-Values were calculated using unpaired Student’s t-test. Data shown are means ± SD. Figure 1—figure supplement 1—source data 1.Original file for western blots displayed in Figure 1—figure supplement 1A and B. Figure 1—figure supplement 1—source data 2.PDF file containing original western blots for Figure 1—figure supplement 1A and B indicating relevant bands and treatments. Figure 1—figure supplement 1—source data 3.Excel file for the data in Figure 1—figure supplement 1C.

### Cancer cells with elevated NAD+/NADH ratios following serine withdrawal exhibit greater serine synthesis

**Figure 2.**
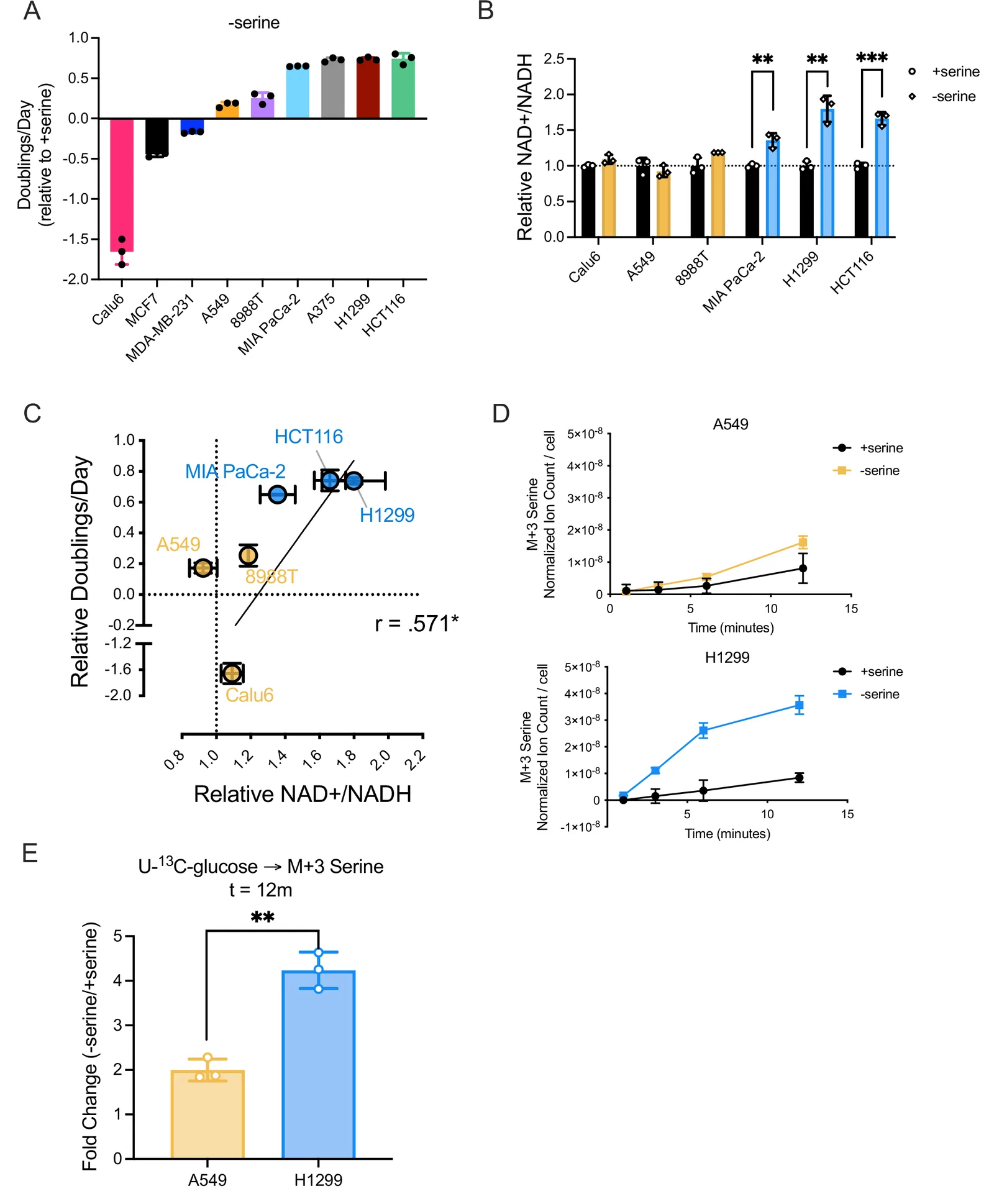
The NAD+/NADH ratio differs between cancer cells upon serine withdrawal and correlates with proliferation in serine-depleted conditions. (**A**) Proliferation rate (doublings per day) of indicated cancer cells cultured without serine for 72 hr normalized to proliferation rate of corresponding cancer cells cultured with serine, n=3. (**B**) NAD+/NADH ratios of indicated cells cultured with or without serine for 24 hr. Yellow indicates cells with unaltered NAD+/NADH ratios (redox non-responder cells), and blue indicates cells with elevated NAD+/NADH ratios when cultured without serine (redox responder cells). Values are normalized to the NAD+/NADH ratios of cells cultured with serine, n=3. p-Values were calculated by unpaired Student’s t-test, **p<0.01, ***p<0.005. (**C**) Relative NAD+/NADH ratios of cells cultured without serine are plotted against proliferation rates of corresponding cells cultured without serine. Data points are labeled to denote the different cell lines (Calu6, A549, 8988T, MIA PaCa-2, H1299, and HCT116). Pearson correlation coefficient and p-values were calculated by simple linear regression, *p<0.05. (**D**) Serine labeled (M+3) from U-^13^C-glucose over time in A549 and H1299 cells cultured with or without serine for 24 hr prior to U-^13^C-glucose exposure. Serine levels were normalized to internal norvaline standard, and cell number measured in indicated conditions, n=3. (**E**) Absolute fold change of serine labeled (M+3) from U-^13^C-glucose in A549 and H1299 cells cultured without serine relative to serine labeled (M+3) from U-^13^C-glucose in A549 and H1299 cells cultured with serine for 24 hr after 12 min of U-^13^C-glucose exposure, n=3. p-Values were calculated by unpaired Student’s t-test, **p<0.01. Data shown for all panels are means ± SD. Figure 2—source data 1.Excel file for the data in Figure 2A, B, D and E.

Based on our finding that the NAD+/NADH ratio correlates with serine synthesis rate, we wondered whether endogenous cell NAD+/NADH ratios, which can differ across cancer cells, are predictive of proliferation rate following serine deprivation. To test this, we measured how proliferation rate varied in a panel of cancer cells derived from various tissues-of-origin and with different genetic driver mutations, and found a wide range of sensitivity to serine deprivation (Figure 2A, Figure 2—figure supplement 1A, Supplementary file 1). Because PHGDH expression is important for proliferation in serine-depleted conditions (DeNicola et al., 2015; Locasale et al., 2011; Maddocks et al., 2017; Possemato et al., 2011; Sullivan et al., 2019b), we first assessed the protein expression of the serine synthesis enzymes PHGDH, PSAT1, and PSPH across the different cancer cells to examine how well this explained environmental serine dependence. As expected, cells with low serine synthesis enzyme protein levels (MCF7, MDA-MB-231) were most sensitive to serine withdrawal while cells with higher serine synthesis enzyme protein levels were more resistant to serine withdrawal (PHGDH-overexpressing MDA-MB-231, A375; Figure 2—figure supplement 1A-C). However, for many cancer cells (Calu6, A549, 8988T, MIA PaCa-2, H1299, HCT116), serine synthesis enzyme expression alone could not fully explain sensitivity to serine withdrawal (Figure 2—figure supplement 1C). For example, when comparing the non-small cell lung cancer cells A549 and H1299, H1299 cells express less PHGDH protein and similar PSAT1 and PSPH protein levels than A549 cells but are more resistant to serine deprivation (Figure 2—figure supplement 1B-E). Consistent with lower PHGDH expression, H1299 cells express lower levels of NRF2, a transcriptional regulator of serine biosynthesis enzymes (DeNicola et al., 2015). When examining the cancer cells where serine synthesis enzyme expression could not fully explain the differences in proliferation upon serine depletion, there was a surprising negative correlation between proliferation and PHGDH protein expression, no relationship between proliferation and PSAT1 protein expression, and a positive correlation between proliferation and PSPH protein expression (Figure 2—figure supplement 1F). Given our finding that the NAD+/NADH ratio correlates with serine synthesis rate, we hypothesized that differences in the NAD+/NADH ratio upon serine deprivation could contribute to the variability in sensitivity to serine withdrawal across cells with similar serine synthesis pathway protein levels. To test this, we measured the cell NAD+/NADH ratios in the presence and absence of exogenous serine. Interestingly, we find that serine starvation elevated the NAD+/NADH ratio in some cells, which we term ‘redox responder cells’ and label in blue. In contrast, we also find that certain cancer cells minimally change the NAD+/NADH ratio in response to serine depletion, which we term ‘redox non-responder cells’ and label in yellow (Figure 2B, Figure 2—figure supplement 1G). Of note, whether the NAD+/NADH ratio of a cell was more or less oxidized in serine-replete conditions was not predictive of response to serine withdrawal (Figure 2—figure supplement 1G). Strikingly, redox responder cells were more resistant to serine depletion compared to redox non-responder cells (Figure 2C). The NAD+/NADH ratio was also elevated in cancer cells with higher serine synthesis enzyme levels upon serine depletion and were the most resistant to serine depletion (A375, PHGDH-overexpressing MDA-MB-231; Figure 2—figure supplement 1B,C,G). To confirm that an elevated NAD+/NADH ratio in response to serine depletion was associated with elevated serine synthesis, we compared how serine synthesis rates changed following serine withdrawal in A549 and H1299 cells. We performed focused comparisons between A549 and H1299 cells because they exhibit differences in proliferation upon serine deprivation that are not explained by PHGDH protein expression, demonstrate differing responses of the cell NAD+/NADH ratio upon serine deprivation, and have similar basal proliferation rates. We find that after 24 hr in serine-depleted conditions, H1299 cells demonstrated a higher rate of serine synthesis, a greater increase in serine synthesis, and greater intracellular serine levels than A549 cells, consistent with the elevated NAD+/NADH ratio in H1299 cells (Figure 2D and E; Figure 2—figure supplement 1H,I). Importantly, we confirmed kinetic U-^13^C-glucose tracing was performed at metabolic steady state by ensuring metabolite levels were stable at each collected time point (Figure 2—figure supplement 1I). Taken together, these data suggest that in some cells, the cell NAD+/NADH ratio is responsive to serine availability. Moreover, increases in the NAD+/NADH ratio correlate with increased serine synthesis and proliferation rates in serine-depleted conditions independent of PHGDH protein expression.

**Figure 2—figure supplement 1.**
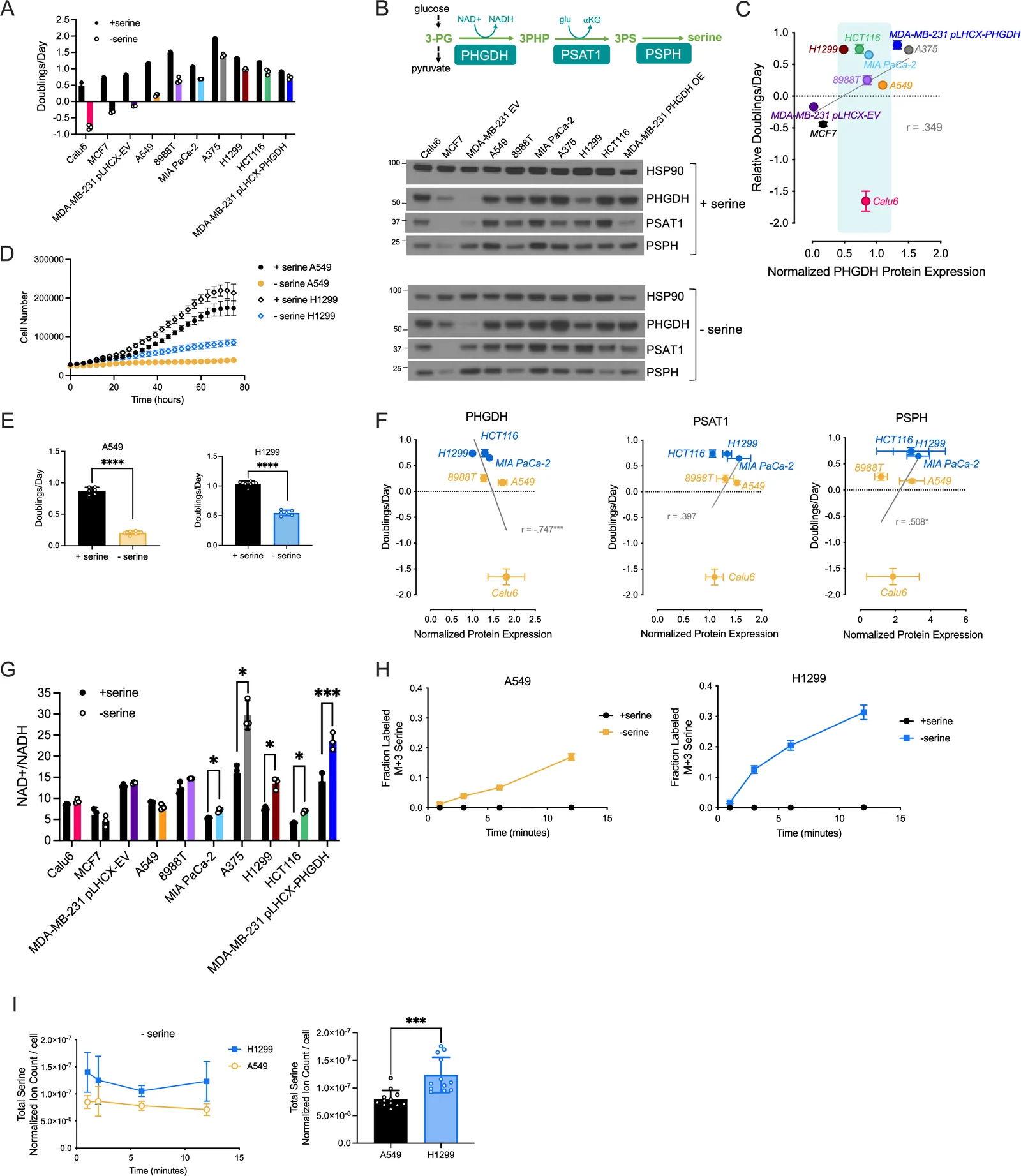
Relationship between PHGDH protein expression, cell NAD+/NADH ratio, and cell proliferation rate following serine deprivation. (**A**) Proliferation rates (doublings per day) of cells cultured with or without serine for 72 hr, n=3. (**B**) Above: Schematic of the serine synthesis pathway. Below: Immunoblot assessing protein levels of PHGDH, PSAT1, and PSPH in the indicated cells cultured with or without serine for 24 hr. HSP90 protein expression is shown as a loading control. These data are representative of three independent experiments. 3PG, 3-phosphoglycerate; PHGDH, phosphoglycerate dehydrogenase; 3PHP, 3-phosphohydroxypyruvate; glu, glutamate; αKG, α-ketoglutarate; PSAT1, phosphoserine aminotransferase 1; 3PS, 3-phosphoserine; PSPH, phosphoserine phosphatase. (**C**) Quantification of PHGDH protein expression for the indicated cells under serine-depleted conditions (normalized to HSP90 protein expression) from three biological replicates, correlated with the corresponding relative proliferation rate of indicated cells under serine-depleted conditions based on data presented in (**A**). Cell lines enclosed in the light blue shaded box indicate cancer cell lines with serine synthesis enzyme expression that could not fully predict proliferation in the absence of serine that we chose to examine further. Pearson correlation coefficient calculated by simple linear regression. (**D**) Cell number over time for either A549 cells (yellow) or H1299 cells (blue) in either serine-replete or serine-depleted conditions, n=6. (**E**) Calculated doublings per day from the data in (D) for the indicated cells and conditions, n=6. p-Values were calculated using unpaired Student’s t-test, ****p<0.001. (**F**) Correlation between the protein expression of serine synthesis enzymes PHGDH, PSAT1, and PSPH across three biological replicates and proliferation rate in serine-depleted conditions for the indicated cell lines, n=3. Pearson correlation coefficient and p-values were calculated by simple linear regression, *p<0.05, ***p<0.005. (**G**) Unnormalized NAD+/NADH ratios measured for the indicated cell lines cultured with or without serine for 24 hr, n=3 per each cell line. p-Values were calculated by unpaired Student’s t-test, *p<0.05, ***p<0.005. (**H**) Fraction of total serine labeled from U-^13^C-glucose over time in A549 and H1299 cells that were cultured with or without serine for 24 hr prior to U-^13^C-glucose exposure as indicated, n=3. (**I**) Left: Total intracellular serine levels over time from the kinetic tracing with A549 and H1299 cells cultured with or without serine for 24 hr prior to U-^13^C-glucose exposure as described in (**H**). Right: Average of total intracellular serine levels from all time points of the kinetic U-^13^C-glucose isotope tracing experiment shown in (**H**). Serine levels were normalized to internal norvaline standard and cell number. p-Values were calculated by unpaired Student’s t-test, ***p<0.005. Data shown for all panels are means ± SD. Figure 2—figure supplement 1—source data 1.Original file for western blots 1160 in Figure 2—figure supplement 1B, exposure 1. Figure 2—figure supplement 1—source data 2.Original file for western blots in Figure 2—figure supplement 1B, exposure 2. Figure 2—figure supplement 1—source data 3.Original file for western blots in Figure 2—figure supplement 1B, exposure 3. Figure 2—figure supplement 1—source data 4.PDF file containing original western blots in Figure 2—figure supplement 1B, exposure 1, indicating the relevant bands and treatments. Figure 2—figure supplement 1—source data 5.PDF file containing original western blots in Figure 2—figure supplement 1B, exposure 2, indicating the relevant bands and treatments. Figure 2—figure supplement 1—source data 6.PDF file containing original western blots in Figure 2—figure supplement 1B, exposure 3, indicating the relevant bands and treatments. Figure 2—figure supplement 1—source data 7.Excel file for the data in Figure 2—figure supplement 1A, C, D, F, G, H and I.

### Increased mitochondrial respiration is associated with an elevated NAD+/NADH ratio and increased serine synthesis

**Figure 3.**
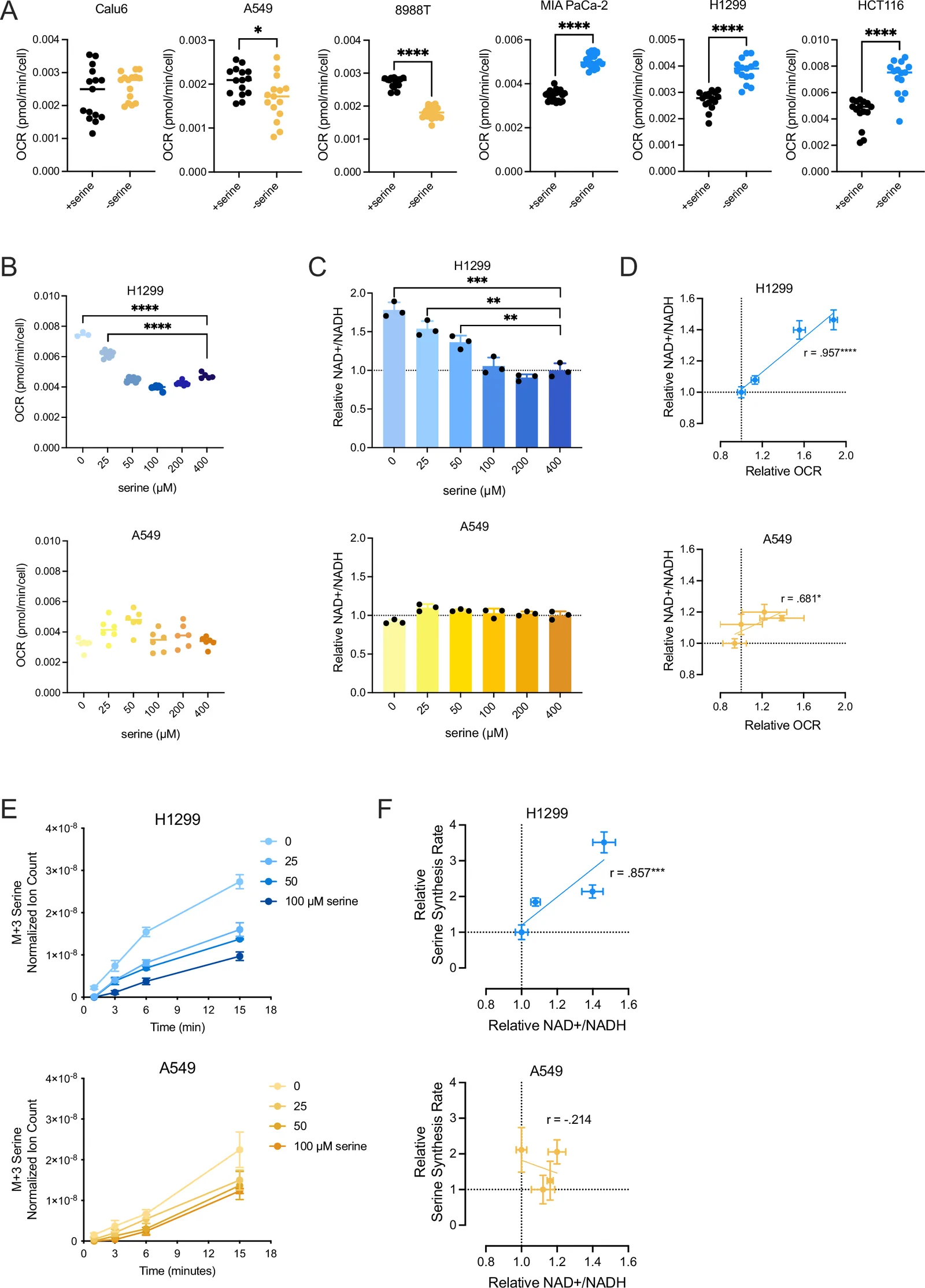
Elevated mitochondrial respiration is associated with increased NAD+/NADH ratios and serine synthesis rates. (**A**) Oxygen consumption rate (OCR) of indicated cells cultured with or without serine for 24 hr. Values are averages of three repeat measurements, n=14–20. p-Values were calculated by unpaired Student’s t-test, *p<0.05, ****p<0.001. (**B**) OCR of cells (H1299 top, A549 bottom) cultured with indicated concentrations of serine for 24 hr. Values are averages of three repeat measurements, n=3–9. p-Values were calculated by unpaired Student’s t-test, ****p<0.001. (**C**) NAD+/NADH ratios of cells (H1299 top, A549 bottom) cultured with indicated concentrations of serine for 24 hr. Values are normalized to the NAD+/NADH ratio of cells cultured in 400 μM serine (serine concentration in DMEM), n=3. p-Values were calculated by unpaired Student’s t-test, **p<0.01, ***p<0.005. (**D**) Relative OCR plotted against relative NAD+/NADH ratios in H1299 and A549 cells cultured in 0, 25, 50, and 100 μM serine for 24 hr. OCR and NAD+/NADH ratios are normalized to measurements from the 100 μM serine condition, n=3. Pearson correlation coefficients and p-values were calculated by simple linear regression, *p<0.05, ****p<0.001. (**E**) Serine labeled (M+3) from U-^13^C-glucose over time in cells (H1299 top, A549 bottom) cultured with indicated serine concentrations for 24 hr prior to U-^13^C-glucose exposure. Serine levels are normalized to internal norvaline standard and cell number measured in indicated conditions, n=3. (**F**) Relative NAD+/NADH ratios of cells (H1299 top, A549 bottom) cultured with 0, 25, 50, and 100 μM serine for 24 hr plotted against relative serine synthesis rates of cells cultured in corresponding conditions. Serine synthesis rates are calculated from labeled serine (M+3) after 1 and 15 min of U-^13^C-glucose exposure and normalized to untreated culture conditions without serine, n=3. Pearson correlation coefficients and p-values were calculated by simple linear regression, ***p<0.005. Data shown for all panels are means ± SD. Figure 3—source data 1.Excel file for the data in Figure 3A–F.

The finding that some cancer cells exhibit a more oxidized NAD+/NADH ratio upon serine starvation is surprising, particularly when increased serine synthesis will result in a higher demand for NAD+ to support increased flux through GAPDH and PHGDH (Diehl et al., 2019; Maddocks et al., 2017). We hypothesized that a more oxidized NAD+/NADH ratio could support greater serine synthesis and thus sought to identify the processes that increase the NAD+/NADH ratio in some but not all cancer cells. Lactate production via lactate dehydrogenase (LDH) is a major NAD+ regenerating process, particularly in cancer cells with high glucose fermentation (Fantin et al., 2006; Luengo et al., 2021; Wang et al., 2022; Figure 3—figure supplement 1A); however, lactate secretion relative to glucose consumption was not elevated following serine deprivation in any cells tested (Figure 3—figure supplement 1B). The NAD+ salvage pathway regenerates NAD+ from nicotinamide via nicotinamide phosphoribosyl transferase (NAMPT) and nicotinamide mononucleotide adenylyltransferase (NMNAT) and can be important for supporting PHGDH-driven serine synthesis (Figure 3—figure supplement 1A; Murphy et al., 2018). While A549 cells were more sensitive to the NAMPT inhibitor FK866 in serine-replete conditions, there was no difference in the sensitivity of A549 and H1299 cells to FK866 in serine-depleted conditions (Figure 3—figure supplement 1C). These data suggest that redox responder and non-responder cells do not have differential dependency on the NAD+ salvage pathway when cultured without serine. Moreover, supplementing redox non-responder A549 cells with nicotinamide mononucleotide (NMN), a precursor to NAD+, did not improve proliferation in serine-depleted conditions (Figure 3—figure supplement 1D). Mitochondrial respiration is another major way cells regenerate NAD+ from NADH (Handy and Loscalzo, 2012). Thus, we tested whether changes in mitochondrial respiration, as measured by cellular oxygen consumption rate (OCR), accompany the increase in the NAD+/NADH ratio following serine withdrawal. Indeed, cells that increased their NAD+/NADH ratio following serine deprivation also exhibited increased OCR, whereas cells with unaltered NAD+/NADH ratios following serine withdrawal did not (Figure 3A, Figure 3—figure supplement 1E). To test whether elevated oxygen consumption was related to serine deprivation, we cultured H1299 and A549 cells in varying amounts of extracellular serine and measured OCR. Oxygen consumption of A549 cells was not impacted by varying extracellular serine levels, while oxygen consumption of H1299 cells increased as extracellular serine decreased below 100 μM, the serine concentration where proliferation begins to be affected by serine limitation (Figure 3B, Figure 3—figure supplement 1F). The increase in OCR also corresponded with the NAD+/NADH ratio at different serine levels in H1299 cells, while the NAD+/NADH ratio in A549 cells was unchanged by extracellular serine availability (Figure 3C and D). Consistently, the NAD+/NADH ratio of H1299 cells cultured in different concentrations of serine positively correlated with serine synthesis, whereas A549 cells displayed minimal changes in serine synthesis rate (Figure 3E and F). Notably, PHGDH protein levels remained constant as extracellular serine levels were decreased in both A549 and H1299 cells (Figure 3—figure supplement 1G). Together, these findings suggest that elevated NAD+/NADH ratios in response to decreasing environmental serine are linked to elevated mitochondrial respiration in select cancer cells and correlate with increased serine synthesis.

**Figure 3—figure supplement 1.**
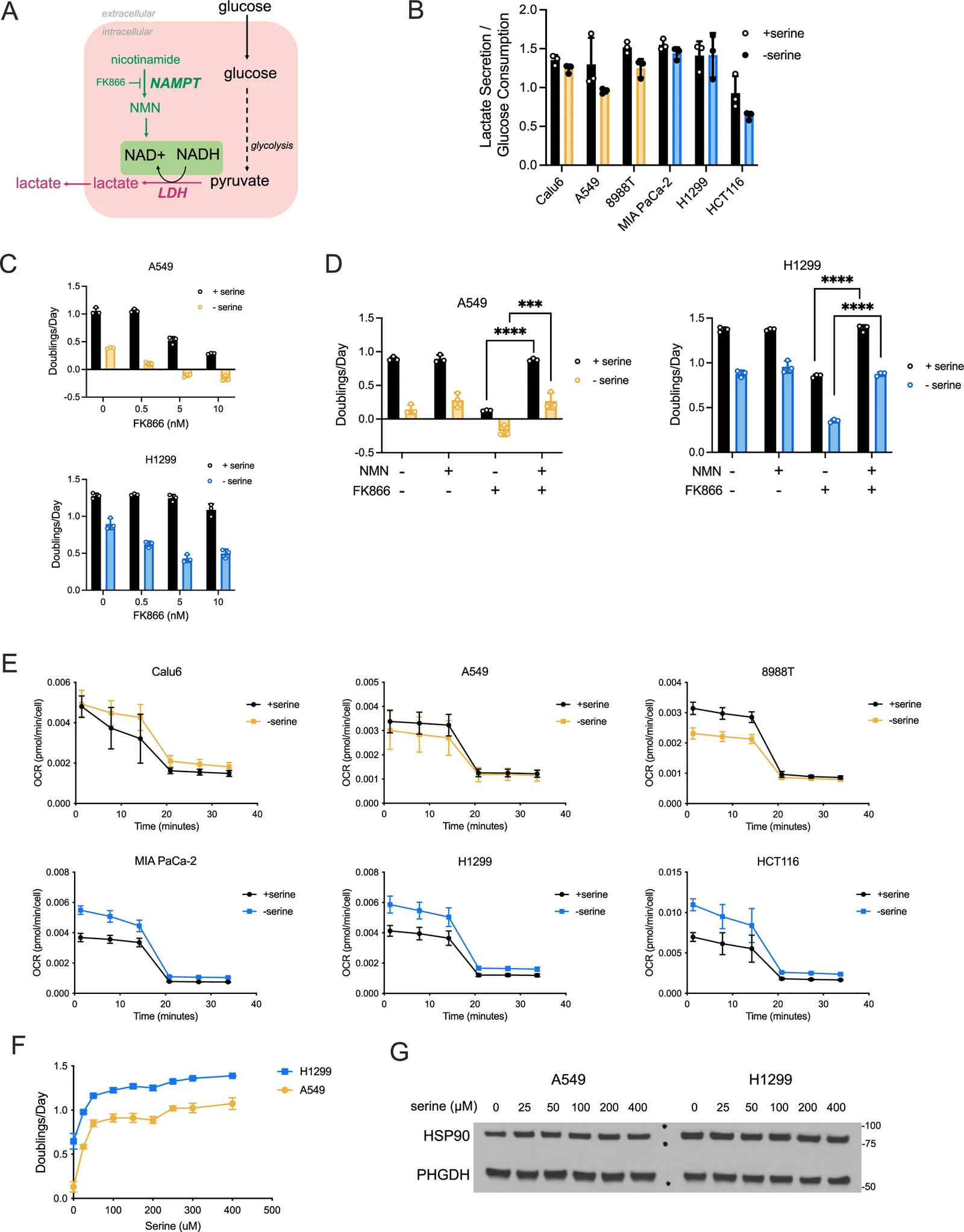
Lactate dehydrogenase (LDH) activity, reliance on the NAD+ salvage pathway, mitochondrial respiration, and PHGDH protein levels following serine deprivation. (**A**) Schematic depicting the contributions of LDH and the NAD+ salvage pathway to NAD+ generation. Glucose is consumed by cells and converted to pyruvate via glycolysis. LDH can convert pyruvate to lactate by oxidizing NADH to NAD+. In the NAD+ salvage pathway, nicotinamide can be converted to nicotinamide mononucleotide (NMN) by the enzyme nicotinamide phosphoribosyltransferase (NAMPT), which is inhibited by the small molecule FK866. NMN is subsequently converted to NAD+ via NMN adenylyltransferase. (**B**) Lactate secretion rate normalized to glucose consumption rate as measured in the indicated cells cultured with or without serine for 72 hr, n=3. (**C**) Proliferation rate (doublings per day) of A549 (above) and H1299 cells (below) cultured with or without serine and with or without the indicated concentrations of FK866 for 72 hr, n=3. There was a statistically significant difference in the sensitivity of A549 and H1299 cells to FK866 in the serine-replete condition (p<0.001), but not in the serine-depleted conditions. p-Values were calculated by ANCOVA analysis followed by post-hoc Tukey HSD test. (**D**) Proliferation rate (doublings per day) of A549 (left) and H1299 cells (right) cultured with or without serine and either NMN (100 μM) or FK866 (10 μM) as indicated for 72 hr, n=3. p-Values were calculated using unpaired Student’s t-test, ***p<0.005, ****p<0.001. (**E**) Oxygen consumption rate (OCR) of cells cultured with or without serine for 24 hr, n=10–20. Rotenone and antimycin A were injected between the third and fourth OCR measurements to define non-mitochondrial OCR. Data shown are normalized to cell number. (**F**) Proliferation rate (doublings per day) of A549 and H1299 cells cultured in media with the indicated concentrations of serine for 72 hr. Media was replenished every day to avoid serine depletion in lower serine containing conditions, n=3. (**G**) Immunoblot assessing PHGDH protein levels in A549 or H1299 cells cultured in media with the indicated concentrations of serine for 24 hr. HSP90 expression is shown as a loading control. Data shown for all panels are means ± SD. Figure 3—figure supplement 1—source data 1.Original file for western blots in Figure 3—figure supplement 1G. Figure 3—figure supplement 1—source data 2.PDF file for western blots in Figure 3—figure supplement 1G indicating the relevant bands and treatments. Figure 3—figure supplement 1—source data 3.Excel file for the data in Figure 3—figure supplement 1B–F.

### Mitochondrial respiration governs the cell NAD+/NADH ratio and influences serine synthesis rate

**Figure 4.**
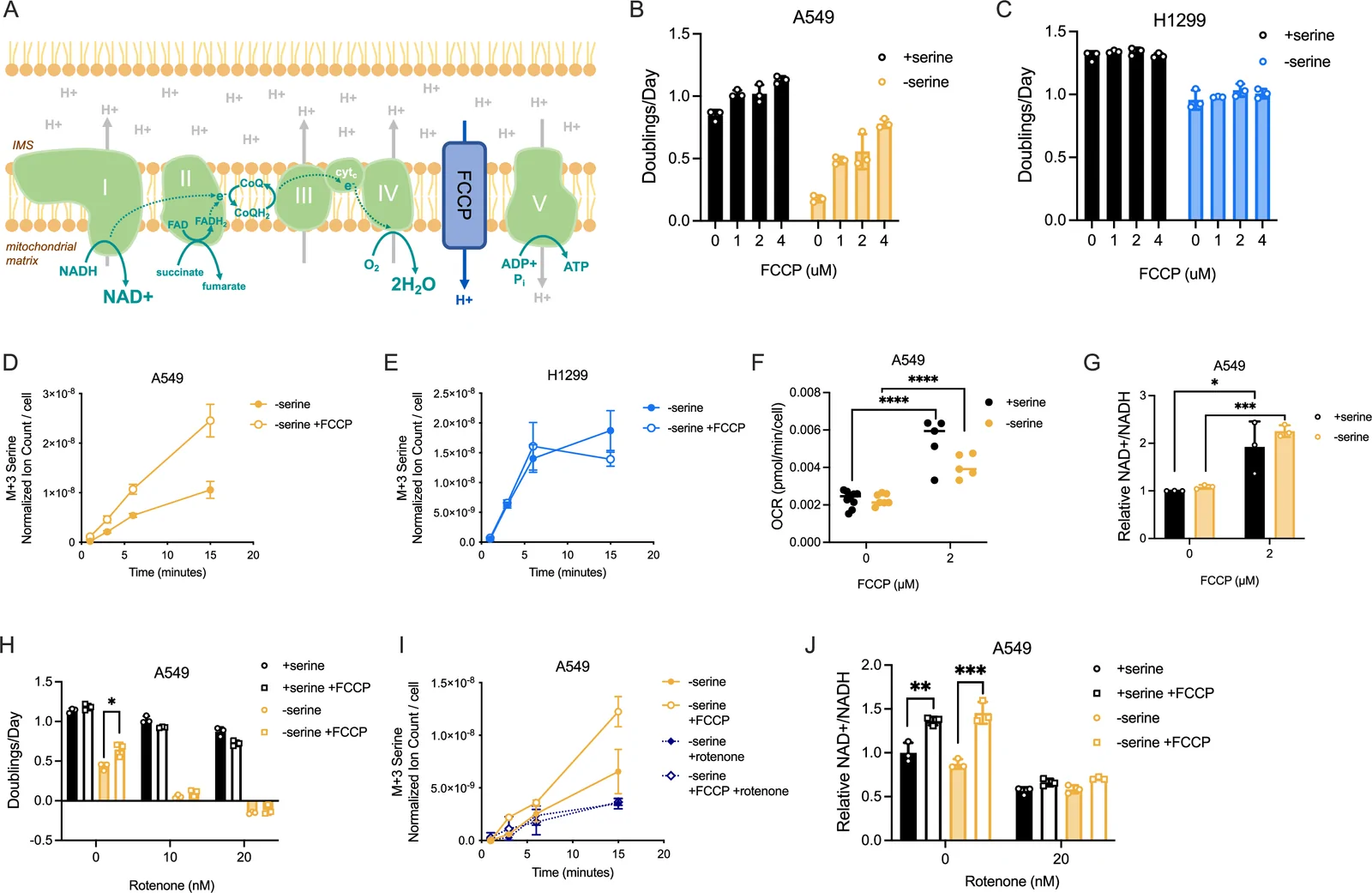
Mitochondrial respiration governs the endogenous cell NAD+/NADH ratio and influences serine synthesis. (**A**) Schematic depicting the mitochondrial electron transport chain (ETC) and the protonophore activity of FCCP that uncouples the ETC from ATP synthase activity. CoQ(H_2_) indicates the oxidized and reduced (H_2_) forms of coenzyme Q, cyt_c_ denotes cytochrome c. (**B,C**) Proliferation rate (doublings per day) of A549 (**B**) and H1299 cells (**C**) cultured with or without serine with indicated concentrations of FCCP for 72 hr, n=3. Increasing doses of FCCP statistically increased proliferation of A549 cells cultured without serine (p<0.001). p-Values were calculated by simple linear regression. (**D**) Serine labeled (M+3) from U-^13^C-glucose over time in A549 cells without serine and with or without 2 μM FCCP treatment for 24 hours prior to U-^13^C-glucose exposure. Serine levels are normalized to internal norvaline standard and cell number measured in indicated conditions, n=3. (**E**) Serine labeled (M+3) from U-^13^C-glucose over time in H1299 cells without serine, and with or without 2 μM FCCP treatment for 24 hr prior to U-^13^C-glucose exposure. Serine levels are normalized to internal norvaline standard and cell number measured in indicated conditions, n=3. (**F**) Oxygen consumption rate (OCR) of A549 cells cultured with or without serine with indicated FCCP treatment for 24 hr. Values are averages of three repeat measurements, n=5–9. p-Values were calculated by unpaired Student’s t-test, ****p<0.001. (**G**) Relative NAD+/NADH ratio of A549 cells cultured with or without serine with indicated FCCP treatment for 24 hr. NAD+/NADH ratios are normalized to the NAD+/NADH ratio in A549 cells cultured with serine, n=3. p-Values were calculated by unpaired Student’s t-test, *p<0.05, ***p<0.005. (**H**) Proliferation rate (doublings per day) of A549 cells cultured with or without serine, 2 μM FCCP, and indicated concentrations of rotenone for 72 hr, n=3. p-Values were calculated by unpaired Student’s t-test, *p<0.05. (**I**) Serine labeled (M+3) from U-^13^C-glucose over time in A549 cells cultured without serine and with or without 2 μM FCCP or 20 nM rotenone as indicated for 24 hr prior to U-^13^C-glucose exposure. Serine levels are normalized to internal norvaline standard and cell number measured in indicated conditions, n=3. (**J**) Relative NAD+/NADH ratio of A549 cells cultured with or without serine, 2 μM FCCP, or 20 nM rotenone as indicated. NAD+/NADH ratios are normalized to the NAD+/NADH ratio in A549 cells cultured with serine, n=3. p-Values were calculated by unpaired Student’s t-test, **p<0.01, ***p<0.005. Data shown for all panels are means ± SD. Figure 4—source data 1.Excel file for the data in Figure 4B–J.

To test whether increased mitochondrial respiration following serine depletion causes an increase in the cell NAD+/NADH ratio, we treated redox non-responder cells with the proton ionophore FCCP (trifluoromethoxy carbonylcyanide phenylhydrazone), which pharmacologically increases mitochondrial oxygen consumption by uncoupling respiration from ATP synthesis (McLaughlin and Dilger, 1980; Terada, 1990). Increasing respiration with FCCP elevates mitochondrial NAD+ regeneration and the cell NAD+/NADH ratio (Luengo et al., 2021; Figure 4A). Indeed, FCCP treatment improved proliferation of redox non-responder cells in serine-depleted conditions (Figure 4B, Figure 4—figure supplement 1A). In contrast, FCCP did not improve the proliferation of redox responder cells cultured without serine (Figure 4C, Figure 4—figure supplement 1B). Consistent with its impact on proliferation, FCCP treatment led to higher serine synthesis rates only in redox non-responder A549 cells while having no impact on the serine synthesis rates of redox responder H1299 cells (Figure 4D and E, Figure 4—figure supplement 2A-D). As expected, uncoupling mitochondrial respiration from ATP synthase with FCCP led to elevated oxygen consumption in serine-depleted A549 cells (Figure 4F). FCCP also increased oxygen consumption in serine-depleted H1299 cells (Figure 4—figure supplement 1C). Interestingly, FCCP raised the cell NAD+/NADH ratios of both A549 and H1299 cells following serine withdrawal despite only improving serine synthesis and proliferation in A549 cells (Figure 4G, Figure 4—figure supplement 1D). The lack of impact on serine synthesis by FCCP in H1299 cells suggests that NAD +availability may not further constrain serine synthesis in cells that already elevate mitochondrial respiration following environmental serine depletion. In this context, we considered whether another constraint such as PHGDH protein expression was limiting serine synthesis in redox responder cells treated with FCCP. Indeed, overexpressing PHGDH in serine-depleted redox responder H1299 and HCT116 cells fully restored proliferation to that observed in serine-replete conditions (Figure 4—figure supplement 1E,G). In contrast, proliferation of the redox non-responder cells A549 and Calu6 following serine withdrawal had the greatest improvement with simultaneous PHGDH overexpression and FCCP treatment (Figure 4—figure supplement 1F,G). This underscores that changes in both the cell NAD+/NADH ratio and relevant enzyme expression impact serine synthesis in serine-depleted environments. As an orthogonal approach to FCCP, we constitutively expressed the NADH oxidase from *Lactobacillus brevis* (*Lb*NOX) in both the cytoplasm and the mitochondria of A549 cells (Figure 4—figure supplement 3A). We reasoned that if expressing *Lb*NOX in either the cytoplasm or the mitochondria were to raise the cell NAD+/NADH ratio, proliferation of A549 cells upon serine depletion would be improved. To ensure *Lb*NOX activity was present in cells, we treated cells with rotenone and measured proliferation. As expected, we observed a proliferation improvement when *Lb*NOX was expressed in either the cytoplasm or the mitochondria (Figure 4—figure supplement 3B). However, A549 cyto*Lb*NOX and mito*Lb*NOX cells did not exhibit an elevated cell NAD+/NADH ratio in either serine-replete or serine-depleted conditions (Figure 4—figure supplement 3C), consistent with prior observations (Titov et al., 2016). There was also no change in proliferation upon serine withdrawal when *Lb*NOX was expressed in the cytoplasm or mitochondria (Figure 4—figure supplement 3D).

**Figure 4—figure supplement 1.**
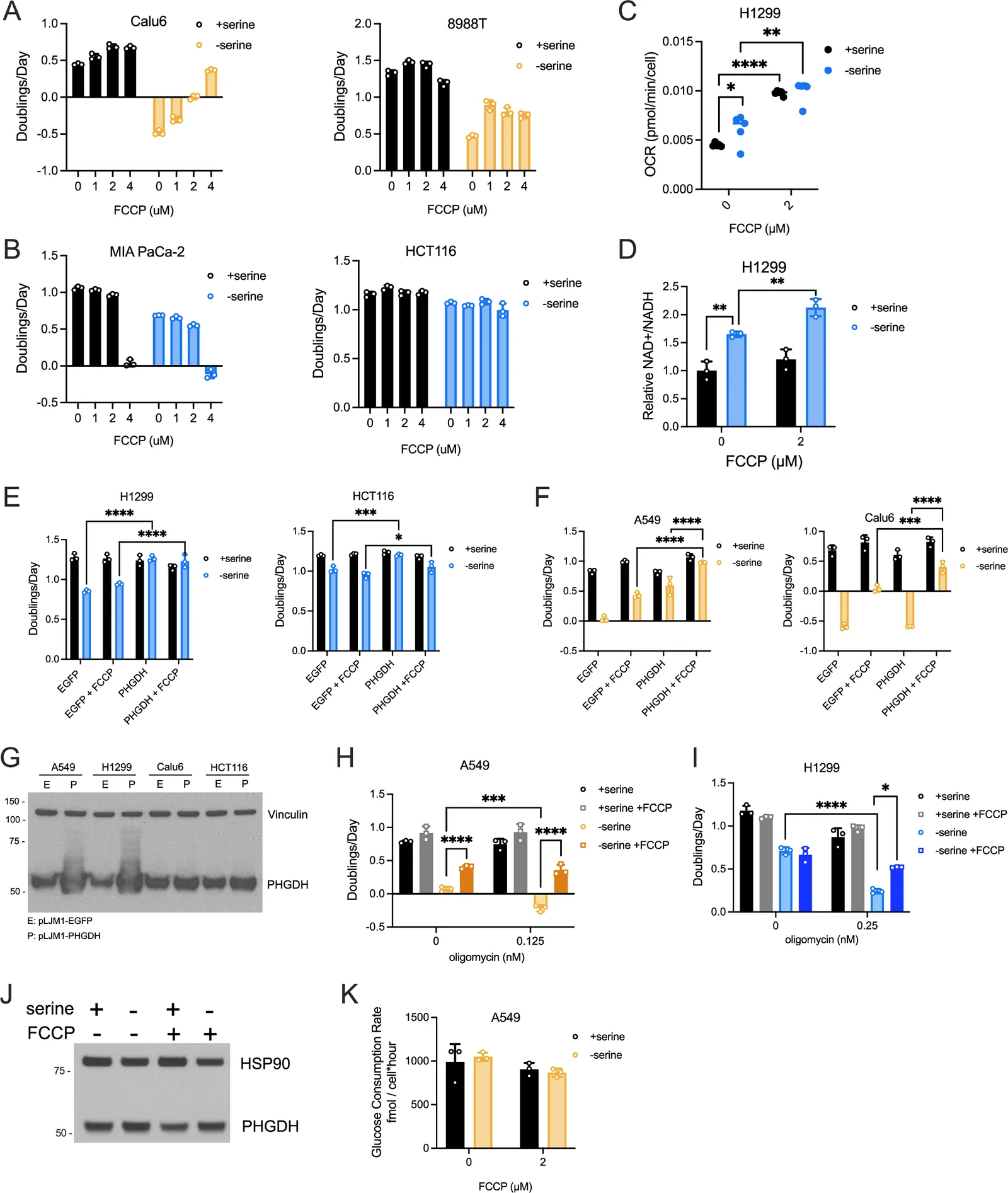
FCCP increases both serine synthesis and proliferation of redox non-responder cells in serine-depleted conditions. (**A**) Proliferation rate (doublings per day) of redox non-responder cells Calu6 and 8988T cultured with or without serine and the indicated concentration of FCCP for 72 hr, n=3. Increasing doses of FCCP statistically increased the proliferation of Calu6 cells cultured without serine, p<0.001. p-Values were calculated by simple linear regression. All doses of FCCP statistically increased the proliferation of 8988T cells cultured without serine but not in a dose-dependent manner, p<0.001. p-Values were calculated by unpaired Student’s t-test. (**B**) Proliferation rate (doublings per day) of redox responder cells MIA PaCa-2 and HCT116 cultured with or without serine and the indicated concentration of FCCP for 72 hr, n=3. (**C**) Mitochondrial oxygen consumption rate (OCR) of H1299 cells cultured with or without serine and the indicated concentration of FCCP for 24 hr, n=5. Values are averages of three repeat measurements. p-Values were calculated by unpaired Student’s t-test, *p<0.05, **p<0.01, ****p<0.001. (**D**) NAD+/NADH ratio measured in H1299 cells cultured with or without serine and the indicated concentration of FCCP for 24 hr, n=3. p-Values were calculated by unpaired Student’s t-test, **p<0.01. (**E**) Proliferation rate (doublings per day) of redox responder cells H1299 and HCT116 with or without PHGDH overexpression and cultured with or without serine and 2 μM FCCP, as indicated, for 72 hr, n=3. P-values were calculated by two-way ANOVA followed by post-hoc Tukey HSD test, *p<0.05, ***p<0.005, ****p<0.001. (**F**) Proliferation rate (doublings per day) of redox non-responder cells A549 and Calu6 with or without PHGDH overexpression and cultured with or without serine and 2 μM FCCP for 72 hr, n=3. p-Values were calculated by two-way ANOVA followed by post-hoc Tukey HSD test, ***p<0.005, ****p<0.001. (**G**) Immunoblot assessing PHGDH protein levels in the indicated cell lines without (pLJM1-EGFP) or with (pLJM1-PHGDH) exogenous PHGDH expression. Vinculin expression is shown as a loading control. (**H,I**) Proliferation rate (doublings per day) of A549 cells (**H**) and H1299 cells (**I**) cultured with or without serine, with or without 2 μM FCCP, or the indicated concentration of oligomycin for 72 hr, n=3. p-Values were calculated by unpaired Student’s t-test, *p<0.05, ***p<0.005, ****p<0.001. (**J**) Immunoblot assessing PHGDH protein levels in A549 cells cultured with or without serine and with or without 2 μM FCCP for 24 hr. HSP90 expression is shown as a loading control. (**K**) Glucose consumption rates of A549 cells cultured with or without serine and 2 μM FCCP for 72 hr, n=3. Data shown for all panels are means ± SD. Figure 4—figure supplement 1—source data 1.Original file for western blots in Figure 4—figure supplement 1G. Figure 4—figure supplement 1—source data 2.PDF file for western blots in Figure 4—figure supplement 1G, indicating the relevant bands and treatments. Figure 4—figure supplement 1—source data 3.Original file for western blots in Figure 4—figure supplement 1J. Figure 4—figure supplement 1—source data 4.PDF file for western blots 1315 in Figure 4—figure supplement 1J, indicating the relevant bands and treatments. Figure 4—figure supplement 1—source data 5.Excel file for the data in Figure 4—figure supplement 1A–F, H, I, and K.

**Figure 4—figure supplement 2.**
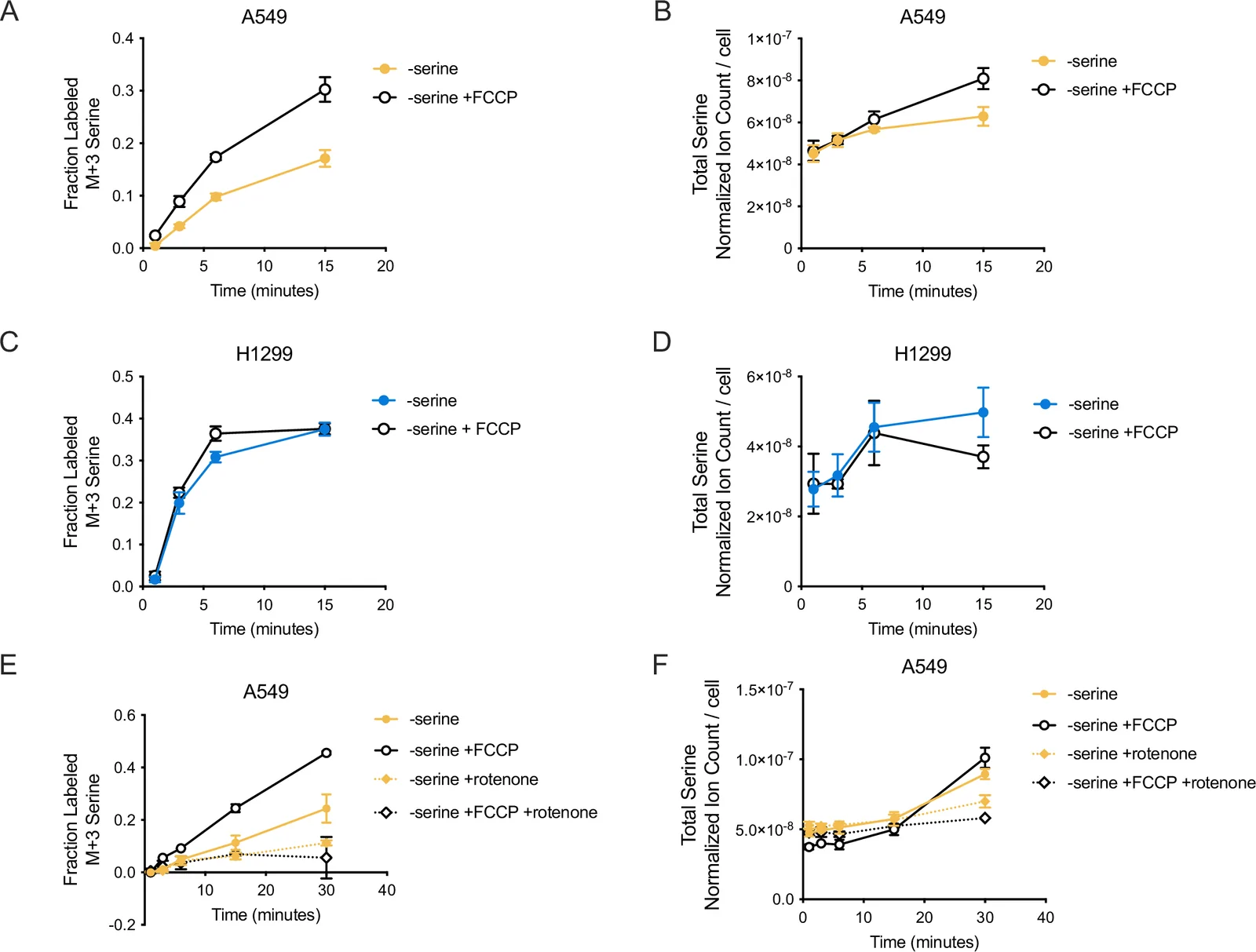
Kinetic tracing of labeled carbon from U-^13^C-glucose into serine following serine withdrawal and FCCP treatment. (**A**) Fraction of total serine labeled (M+3 serine) from U-^13^C-glucose over time in A549 cells cultured without serine, and with or without 2 μM FCCP, for 24 hr prior to U-^13^C-glucose exposure as indicated, n=3. (**B**) Total serine measured in A549 cells cultured without serine and with or without 2 μM FCCP for each time point shown in (**A**), n=3. (**C**) Fraction of total serine labeled (M+3 serine) from U-^13^C-glucose over time in H1299 cells cultured without serine and with or without 2 μM FCCP for 24 hr prior to U-^13^C-glucose exposure as indicated, n=3. (**D**) Total serine measured in H1299 cells cultured without serine and with or without 2 μM FCCP for each time point shown in (**C**), n=3. (**E**) Fraction of total serine labeled (M+3 serine) from U-^13^C-glucose over time in A549 cells cultured without serine and with or without 2 μM FCCP and 20 nM rotenone for 24 hr prior to U-^13^C-glucose exposure as indicated, n=3. (**F**) Total serine measured in A549 cells cultured without serine and with or without 2 μM FCCP and 20 nM rotenone for each time point shown in (**E**), n=3. All serine measurements were normalized to internal norvaline standard and cell number. Data shown for all panels are means ± SD. Figure 4—figure supplement 2—source data 1.Excel file for the data in Figure 4—figure supplement 2A–F.

**Figure 4—figure supplement 3.**
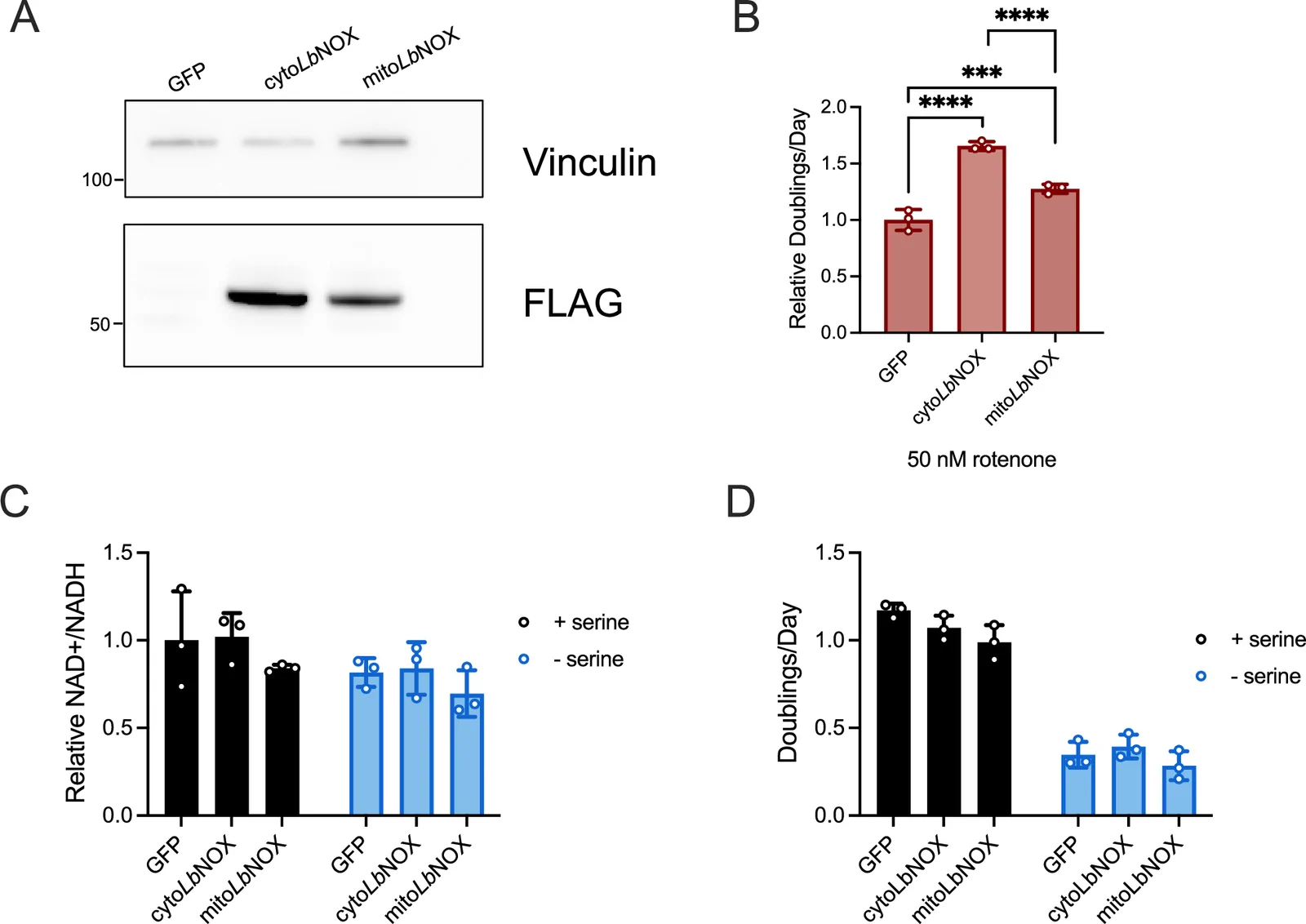
Cytosolic or mitochondrial *Lb*NOX expression does not change the cell NAD+/NADH ratio or proliferation of A549 cells cultured without serine. (**A**) Immunoblot for expression of FLAG-tagged cyto*Lb*NOX or mito*Lb*NOX as indicated. GFP-expressing cells were used as a control. Vinculin expression is shown as a loading control. (**B**) Relative proliferation rate (doublings per day) of A549 cells expressing either cyto*Lb*NOX or mito*Lb*NOX and treated with 50 nM rotenone for 72 hr, n=3. p-Values were calculated using one-way ANOVA followed by post-hoc Tukey HSD test, ***p<0.005, ****p<0.001. (**C**) Relative NAD+/NADH ratios of cells expressing either GFP, cyto*Lb*NOX, or mito*Lb*NOX and cultured with or without serine for 24 hr. NAD+/NADH ratios were normalized to the GFP control cells cultured with serine, n=3. (**D**) Proliferation rate (doublings per day) of cells expressing either GFP, cyto*Lb*NOX, or mito*Lb*NOX and cultured with or without serine for 72 hr, n=3. Data shown for all panels are means ± SD. Figure 4—figure supplement 3—source data 1.Original file for western blots in Figure 4—figure supplement 3A, exposure 1. Figure 4—figure supplement 3—source data 2.Original file for western blots in Figure 4—figure supplement 3A, exposure 2. Figure 4—figure supplement 3—source data 3.PDF file for western blots in Figure 4—figure supplement 3A, exposure 1, indicating the relevant bands and treatments. Figure 4—figure supplement 3—source data 4.PDF file for western blots in Figure 4—figure supplement 3A, exposure 2, indicating the relevant bands and treatments. Figure 4—figure supplement 3—source data 5.Excel file for the data 1365 in Figure 4—figure supplement 3B–D.

**Figure 4—figure supplement 4.**
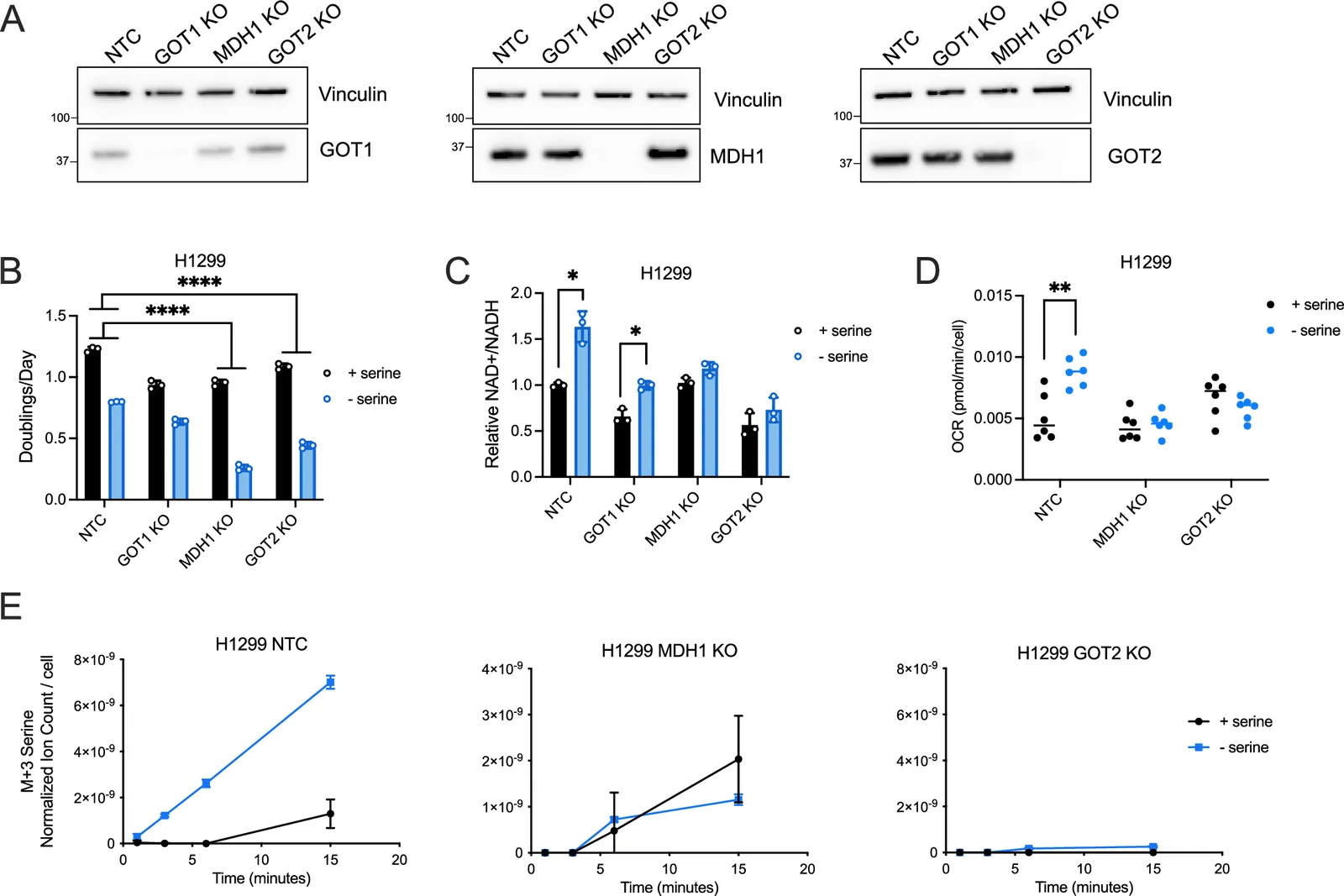
MDH1 and GOT2 support the increase in serine synthesis observed in H1299 cells following serine withdrawal. (**A**) Immunoblots for GOT1 (left), MDH1 (middle), and GOT2 (right) in control cells (NTC, non-targeting control) or cells with knockout (KO) of GOT1, MDH1, or GOT2 as indicated. Vinculin expression is shown as a loading control. (**B**) Proliferation rate (doublings per day) of the indicated cells from (**A**) cultured with or without serine for 72 hr, n=3. p-Values were calculated by nested ANOVA to compare sensitivity to serine depletion between cell lines, ****p<0.001. (**C**) Relative NAD+/NADH ratios of the indicated cells cultured with or without serine for 24 hr, n=3. NAD+/NADH ratios were normalized to H1299 NTC cells cultured with serine. p-Values were calculated using unpaired Student's t-test, *p<0.05. (**D**) Oxygen consumption rate (OCR) of the indicated cells cultured with or without serine for 24 hr, n=6. p-Values were calculated by unpaired Student's t-test, **p<0.01. (**E**) Serine labeled from U-^13^C-glucose (M+3) measured over time from the indicated cells cultured with or without serine for 24 hr prior to U-^13^C-glucose exposure, n=3. Serine levels were normalized to internal standard norvaline and cell number. Data shown for all panels are means ± SD. Figure 4—figure supplement 4—source data 1.Original file for western blots in Figure 4—figure supplement 4A probing for GOT1, GOT2, and the corresponding Vinculin loading control. Figure 4—figure supplement 4—source data 2.Original file for western blots in Figure 4—figure supplement 4A probing for MDH1 and the corresponding Vinculin loading control, exposure. Figure 4—figure supplement 4—source data 3.Original file for western blots 1390 in Figure 4—figure supplement 4A probing for MDH1 and the corresponding Vinculin loading control, exposure 2. Figure 4—figure supplement 4—source data 4.PDF file for western blots in Figure 4—figure supplement 4A probing for GOT1, GOT2, and the corresponding Vinculin loading control, including the raw membrane and molecular weight ladder, indicating the relevant bands and treatments. Figure 4—figure supplement 4—source data 5.PDF file for western blots in Figure 4—figure supplement 4A probing for MDH1 and the corresponding Vinculin loading control, including the raw membrane and molecular weight ladder, indicating the relevant bands and treatments, exposure 1. Figure 4—figure supplement 4—source data 6.PDF file for western blots in Figure 4—figure supplement 4A probing for MDH1 and the corresponding Vinculin loading control, including the raw membrane and molecular weight ladder, indicating the relevant bands and treatments, exposure 2. Figure 4—figure supplement 4—source data 7.Excel file for the data in Figure 4—figure supplement 4B–E.

Because FCCP treatment led to a robust improvement in proliferation of redox non-responder cells cultured without serine, we aimed to test whether FCCP improved serine synthesis and proliferation of redox non-responder cells through oxidizing the NAD+/NADH ratio. We treated cells with the mitochondrial complex I inhibitor rotenone in conjunction with FCCP. Rotenone treatment ablated the ability of FCCP to improve both proliferation and serine synthesis and blocked the ability of FCCP to increase the NAD+/NADH ratio (Figure 4H–J, Figure 4—figure supplement 2E,F). In contrast, treating cells with oligomycin, an ATP synthase inhibitor, did not affect the ability of FCCP to improve proliferation (Figure 4—figure supplement 1H,I). This supports the conclusion that FCCP increases serine synthesis and proliferation in serine-depleted environments by increasing the NAD+/NADH ratio via mitochondrial complex I-mediated NAD+ regeneration. Moreover, FCCP treatment neither increased PHGDH protein expression nor glucose consumption, indicating that improved proliferation and serine synthesis is not caused by changes in enzyme expression or carbon availability for serine synthesis (Figure 4—figure supplement 1J,K). Together, these data argue that mitochondrial respiration, either endogenously increased in response to serine deprivation, or as a result of FCCP treatment, can govern the cell NAD+/NADH ratio and directly influence the serine synthesis rate in response to serine withdrawal.

### The malate aspartate shuttle enzymes MDH1 and GOT2 support elevated mitochondrial respiration in redox responder H1299 cells in serine-depleted environments

The malate aspartate shuttle (MAS) transports reducing equivalents between the cytoplasm and the mitochondria and can be important for de novo serine synthesis (Broeks et al., 2023). Thus, we hypothesized that in serine-deplete H1299 cells, the MAS allows increases in mitochondrial respiration to support cytosolic serine synthesis. To test this, we used CRISPR/Cas9 in H1299 cells to generate isogenic cell lines where either GOT1, MDH1, or GOT2 was deleted (Figure 4—figure supplement 4A). Using these knock-out cell lines, we measured proliferation in serine-depleted conditions and found that MDH1 and GOT2 knock-out cell lines were more sensitive to serine deprivation compared to non-targeting controls (NTC; Figure 4—figure supplement 4B). We then measured the cell NAD+/NADH ratio in knock-out cells cultured without serine and observed that unlike H1299 NTC and GOT1 KO cells, H1299 MDH1 and GOT2 KO cells did not exhibit elevated NAD+/NADH ratios upon serine depletion (Figure 4—figure supplement 4C). Consistently, unlike H1299 NTC cells, H1299 MDH1 and GOT2 KO cells did not demonstrate elevated mitochondrial respiration upon serine depletion (Figure 4—figure supplement 4D). This corresponded with H1299 MDH1 and GOT2 KO cells having blunted serine synthesis rates compared to H1299 NTC cells (Figure 4—figure supplement 4E). Together, this suggests that MDH1 and GOT2 activity support the process by which mitochondrial NAD+ regeneration is transmitted to the cytoplasm to support serine synthesis.

### The NAD+/NADH ratio influences oxidative citrate synthesis in either serine- or lipid-depleted environments

**Figure 5.**
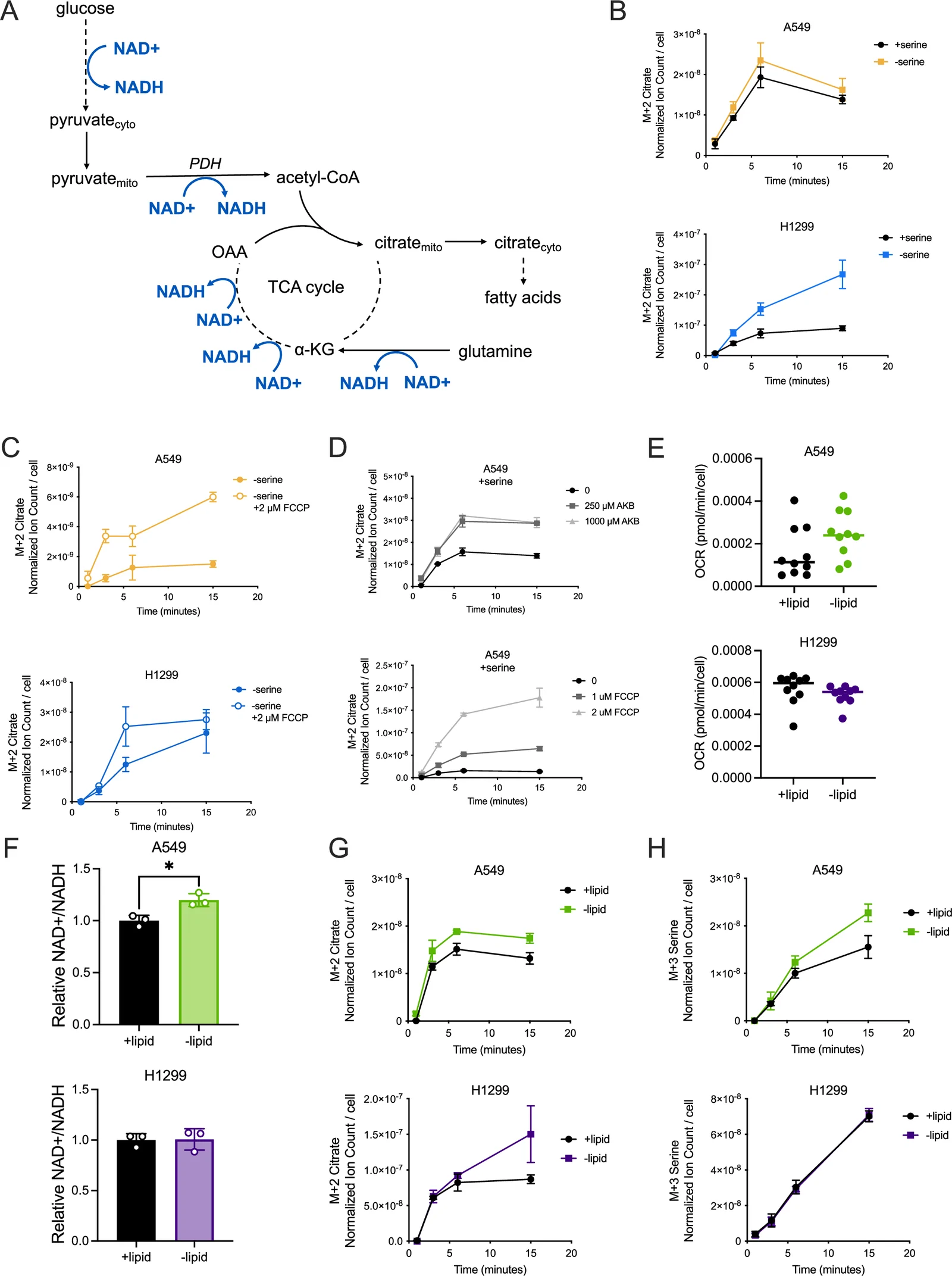
Lipid depletion enhances cell-specific elevation in mitochondrial respiration and the NAD+/NADH ratio, influencing oxidative citrate and serine synthesis. (**A**) Schematic depicting glucose and glutamine oxidative routes for synthesizing citrate, highlighting NAD+ requiring oxidation reactions. (**B**) Citrate labeled (M+2) from U-^13^C-glucose over time in A549 and H1299 cells cultured with or without serine for 24 hr prior to U-^13^C-glucose exposure. Citrate levels are normalized to internal norvaline standard and cell number measured in indicated conditions, n=3. (**C**) Citrate labeled (M+2) from U-^13^C-glucose over time in A549 and H1299 cells cultured without serine and with or without 2 μM FCCP for 24 hr prior to U-^13^C-glucose exposure. Citrate levels are normalized to internal norvaline standard and cell number measured in indicated conditions, n=3. (**D**) Citrate labeled (M+2) from U-^13^C-glucose over time in A549 cells cultured with serine and with indicated treatment conditions for 24 hr prior to U-^13^C-glucose exposure. Citrate levels are normalized to internal norvaline standard and cell number measured in indicated conditions, n=3. (**E**) Oxygen consumption rate (OCR) in A549 and H1299 cells cultured with or without lipids for 24 hr. Values are averages of three repeat measurements, n=10. Green indicates lipid redox responder cells. Purple indicates lipid redox non-responder cells. (**F**) Relative NAD+/NADH ratios of A549 and H1299 cells cultured with or without lipids for 24 hr. NAD+/NADH ratios are normalized to the NAD+/NADH ratio of the corresponding cell type cultured with lipids, n=3. p-Values were calculated by unpaired Student’s t-test, *p<0.05. (**G**) Citrate labeled (M+2) from U-^13^C-glucose over time in A549 and H1299 cells cultured with or without lipids for 24 hr prior to U-^13^C-glucose exposure. Citrate levels are normalized to internal norvaline standard and cell number measured in indicated conditions, n=3. (**H**) Serine labeled (M+3) from U-^13^C-glucose over time in A549 and H1299 cells cultured with or without lipids for 24 hr prior to U-^13^C-glucose exposure. Serine levels are normalized to internal norvaline standard and cell number measured in indicated conditions, n=3. Data shown for all panels are means ± SD. Figure 5—source data 1.Excel file for the data in Figure 5B–H.

Oxidative biosynthetic reactions other than serine synthesis can also be constrained by the NAD+/NADH ratio. For example, cancer cells deprived of environmental lipids increase oxidative citrate production, and we have previously found that citrate synthesis, either through glucose oxidation or glutamine oxidation, is limited by NAD+ availability (Li et al., 2022; Figure 5A, Figure 5—figure supplement 1A). Thus, we sought to uncover whether the increase in the cell NAD+/NADH ratio by mitochondrial respiration in response to serine withdrawal specifically supports greater serine synthesis or also leads to greater oxidative citrate production. To answer this question, we first assessed labeled citrate production using kinetic U-^13^C-glucose tracing in cells that showed increased NAD+/NADH ratios following serine deprivation. We found that the redox responder cells largely showed elevated M+2 citrate production despite no change in environmental lipid availability (Figure 5B; Figure 5—figure supplement 1B-E). In contrast, cells with unaltered NAD+/NADH ratios upon serine depletion did not exhibit higher M+2 citrate production (Figure 5B; Figure 5—figure supplement 1). Consistently, redox responder H1299 cells, but not redox non-responder A549 cells, displayed evidence of elevated citrate production from glutamine either through oxidative decarboxylation or reductive carboxylation, both of which require oxidation reactions that can be limited by electron acceptor availability (Hosios and Vander Heiden, 2018; Li et al., 2022; Figure 5—figure supplement 1I,J). To confirm that elevated M+2 citrate synthesis was driven by an increase in the NAD+/NADH ratio, we treated A549 cells depleted of serine with FCCP and examined glucose-derived citrate synthesis. Indeed, FCCP treatment led to a fourfold increase in M+2 citrate from U-^13^C-glucose in A549 cells depleted of serine, whereas M+2 citrate synthesis was less affected by FCCP in H1299 cells (Figure 5C). The elevated M+2 citrate production in FCCP-treated A549 cells was dependent on mitochondrial complex I, as rotenone prevented the elevated citrate production associated with FCCP treatment (Figure 5—figure supplement 2A-C). To confirm that higher M+2 citrate production following FCCP treatment was related to the NAD+/NADH ratio independent of serine deprivation, we treated A549 cells with increasing doses of AKB and FCCP in serine-replete conditions and found that increasing the NAD+/NADH ratio with either AKB or FCCP led to elevated M+2 citrate synthesis (Figure 5D). This argues that changes to the NAD+/NADH ratio regardless of the nutrient environment can impact citrate synthesis.

**Figure 5—figure supplement 1.**
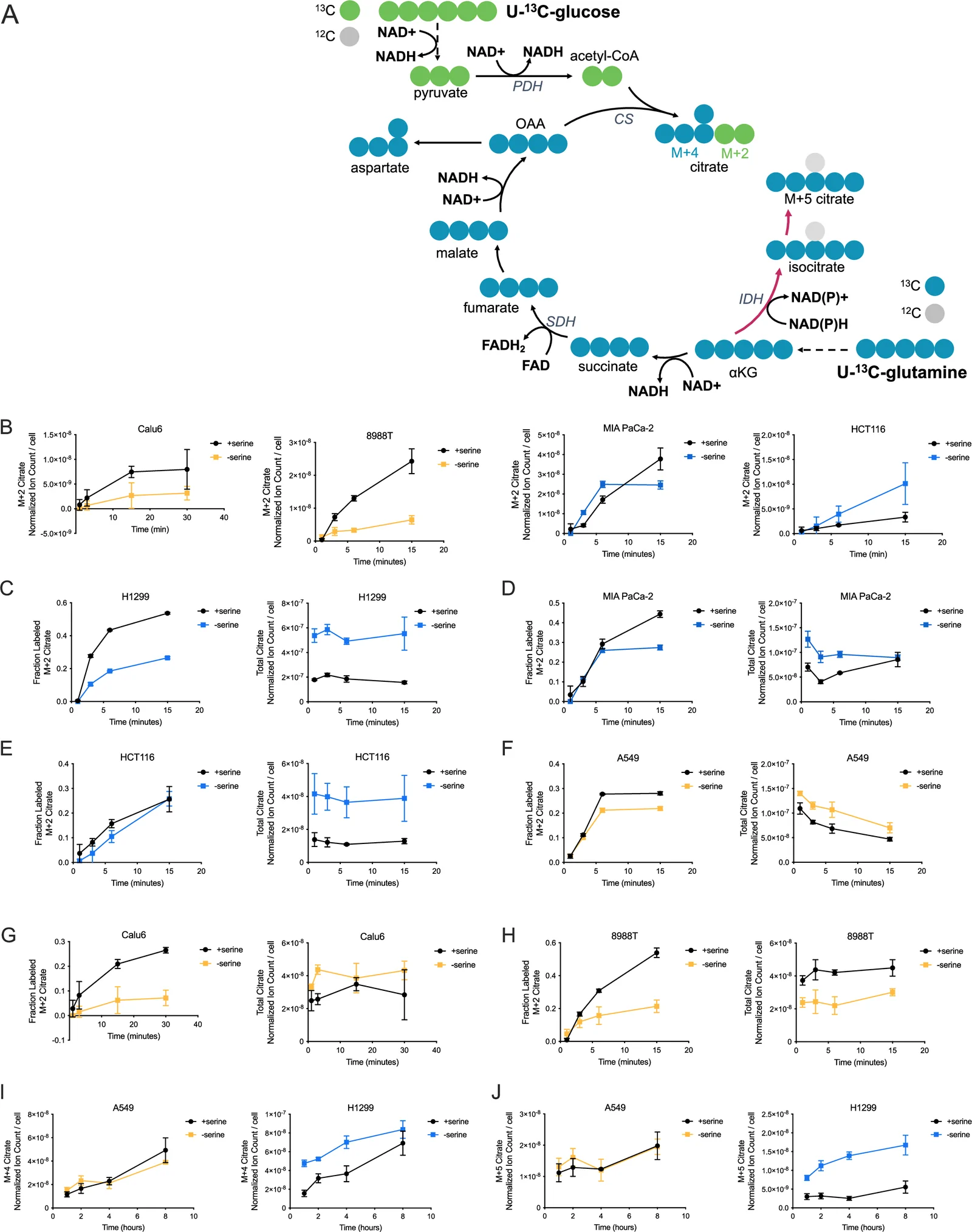
Kinetic tracing of labeled carbon from U-^13^C-glucose and U-^13^C-glutamine into citrate in cells cultured with or without serine. (**A**) Schematic depicting isotopically labeled carbon contribution to citrate from U-^13^C-glucose and U-^13^C-glutamine. PDH, pyruvate dehydrogenase; CS, citrate synthase; IDH, isocitrate dehydrogenase; SDH, succinate dehydrogenase. (**B**) Citrate labeled (M+2) from U-^13^C-glucose over time in Calu6, 8988T, MIA PaCa-2, and HCT116 cells cultured with or without serine for 24 hr prior to U-^13^C-glucose exposure, n=3. (**C-H**) Left of each figure panel: Fraction of citrate labeled (M+2) from U-^13^C-glucose over time in indicated cells cultured with or without serine for 24 hr, n=3. Right of each figure panel: Total citrate levels in the indicated cells cultured with or without serine for 24 hr for each time point shown in left panel, n=3. (**I**) Citrate labeled (M+4) from U-^13^C-glutamine over time in A549 and H1299 cells cultured with or without serine for 24 hr, n=3. (J) Citrate labeled (M+5) from U-^13^C-glutamine over time in A549 and H1299 cells cultured with or without serine for 24 hr, n=3. All citrate measurements were normalized to internal norvaline standard and cell number measured in indicated conditions. Data shown for all panels are means ± SD. Figure 5—figure supplement 1—source data 1.Excel file for the data in Figure 5—figure supplement 1B–J.

**Figure 5—figure supplement 2.**
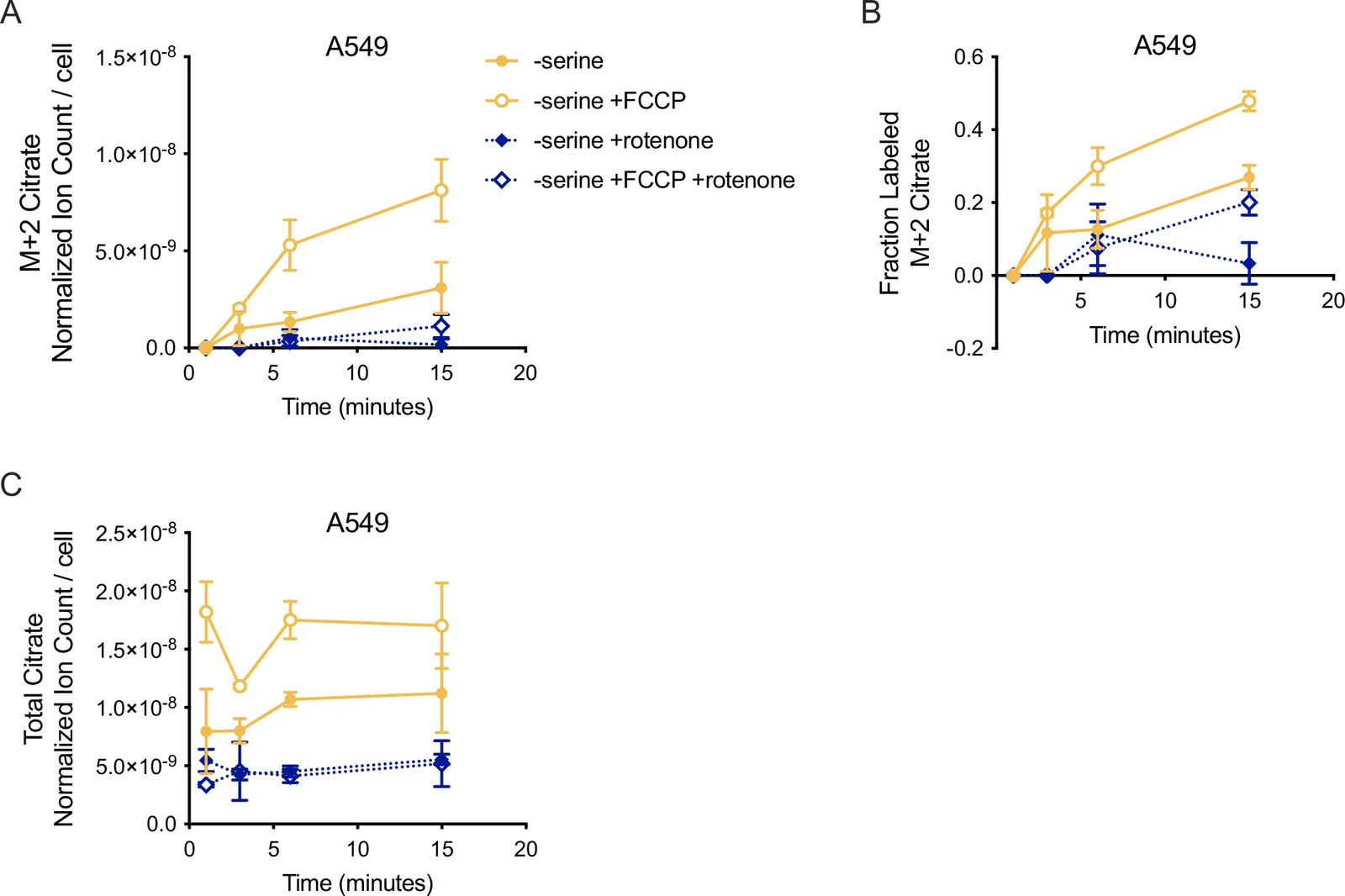
Kinetic tracing of labeled carbon from U-^13^C-glucose into citrate in cells cultured without serine and in the presence or absence of FCCP and/or rotenone. (**A**) Fraction of citrate labeled (M+2) from U-^13^C-glucose over time in A549 cells cultured without serine for 24 hr, and with or without the addition of 2 μM FCCP or 20 nM rotenone as indicated, n=3. (**B**) Fraction of citrate labeled (M+2) from U-^13^C-glucose over time in A549 cells cultured without serine for 24 hr, and with or without the addition of 2 μM FCCP or 20 nM rotenone as indicated, n=3. (**C**) Total citrate levels in A549 cells cultured without serine for 24 hr, and with or without the addition of 2 μM FCCP or 20 nM rotenone as indicated, n=3. All citrate measurements were normalized to internal 1467 norvaline standard and cell number. Data shown for all panels are means ± SD. Figure 5—figure supplement 2—source data 1.Excel file for the data in Figure 5—figure supplement 2A–C.

**Figure 5—figure supplement 3.**
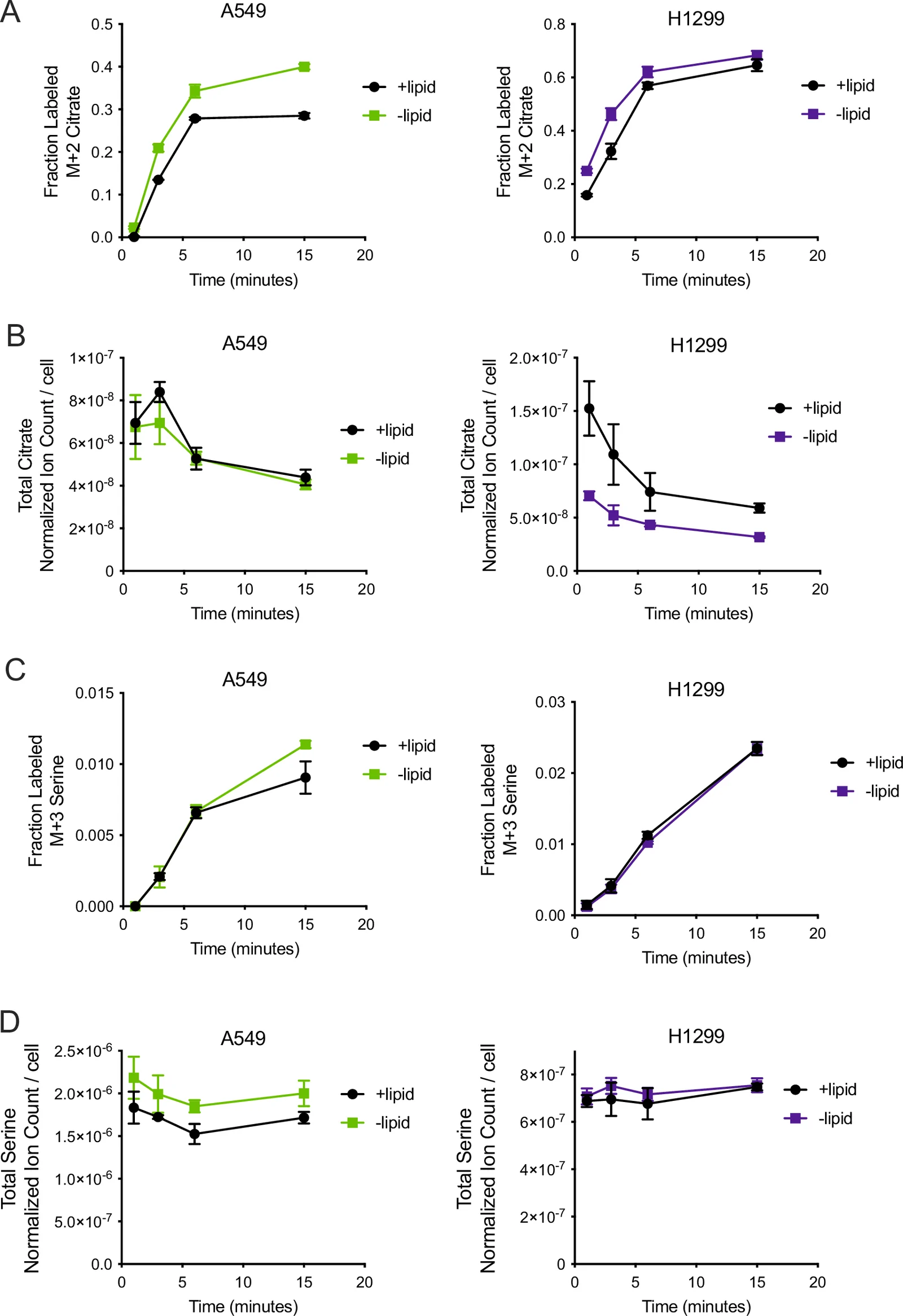
Kinetic tracing of labeled carbon from U-^13^C-glucose into citrate in cells cultured in the presence or absence of lipids. (**A**) Fraction of citrate labeled (M+2) from U-^13^C-glucose over time in A549 and H1299 cells cultured with or without lipids for 24 hr as indicated, n=3. (**B**) Total citrate levels in A549 and H1299 cells cultured with or without lipids for 24 hr for each time point shown in (**A**), n=3. (**C**) Fraction of serine labeled (M+3) from U-^13^C-glucose over time in A549 and H1299 cells cultured with or without lipids for 24 hr as indicated, n=3. (**D**) Total serine levels in A549 and H1299 cells cultured with or without lipids for 24 hr for each time point shown in (**C**), n=3. All citrate and serine measurements were normalized to internal norvaline standard and cell number. Data shown for all panels are means ± SD. Figure 5—figure supplement 3—source data 1.Excel file for the data in Figure 5—figure supplement 3A–D.

We next asked whether increasing mitochondrial respiration in response to low extracellular serine was specific to serine deprivation or represented a general response to any nutrient deprivation that increased NAD+ demand, such as lipid deprivation (Li et al., 2022). To examine this, we depleted A549 and H1299 cells of extracellular lipids by culturing cells in media with lipid-depleted serum (Hosios et al., 2018) and compared OCRs to those of cells cultured in media with lipid-containing serum. Surprisingly, we find that A549 cells, which do not increase mitochondrial respiration in response to serine depletion, showed a trend towards a mildly elevated OCR upon lipid depletion (Figure 5E). On the other hand, H1299 cells did not increase oxygen consumption in lipid-depleted conditions (Figure 5E). We next measured the NAD+/NADH ratio of A549 and H1299 cells depleted of environmental lipids. Consistent with the changes in oxygen consumption, A549 cells displayed an increased NAD+/NADH ratio while H1299 cells did not alter the NAD+/NADH ratio upon lipid deprivation (Figure 5F). Interestingly, both serine and citrate produced via glucose oxidation were elevated in lipid-depleted A549 cells in comparison to H1299 cells, which exhibited unchanged serine synthesis in response to environmental lipid depletion (Figure 5G–H, Figure 5—figure supplement 3). These findings suggest that the NAD+/NADH ratio can be a determinant of both oxidative citrate and serine synthesis, regardless of environmental demand for the nutrient. Additionally, the different metabolic responses to lipid and serine deprivation between cancer cell types suggest that different nutrient environments impact mitochondrial respiration through distinct processes to influence the NAD+/NADH ratio.

### Lipid depletion can enhance proliferation in serine-deprived conditions

**Figure 6.**
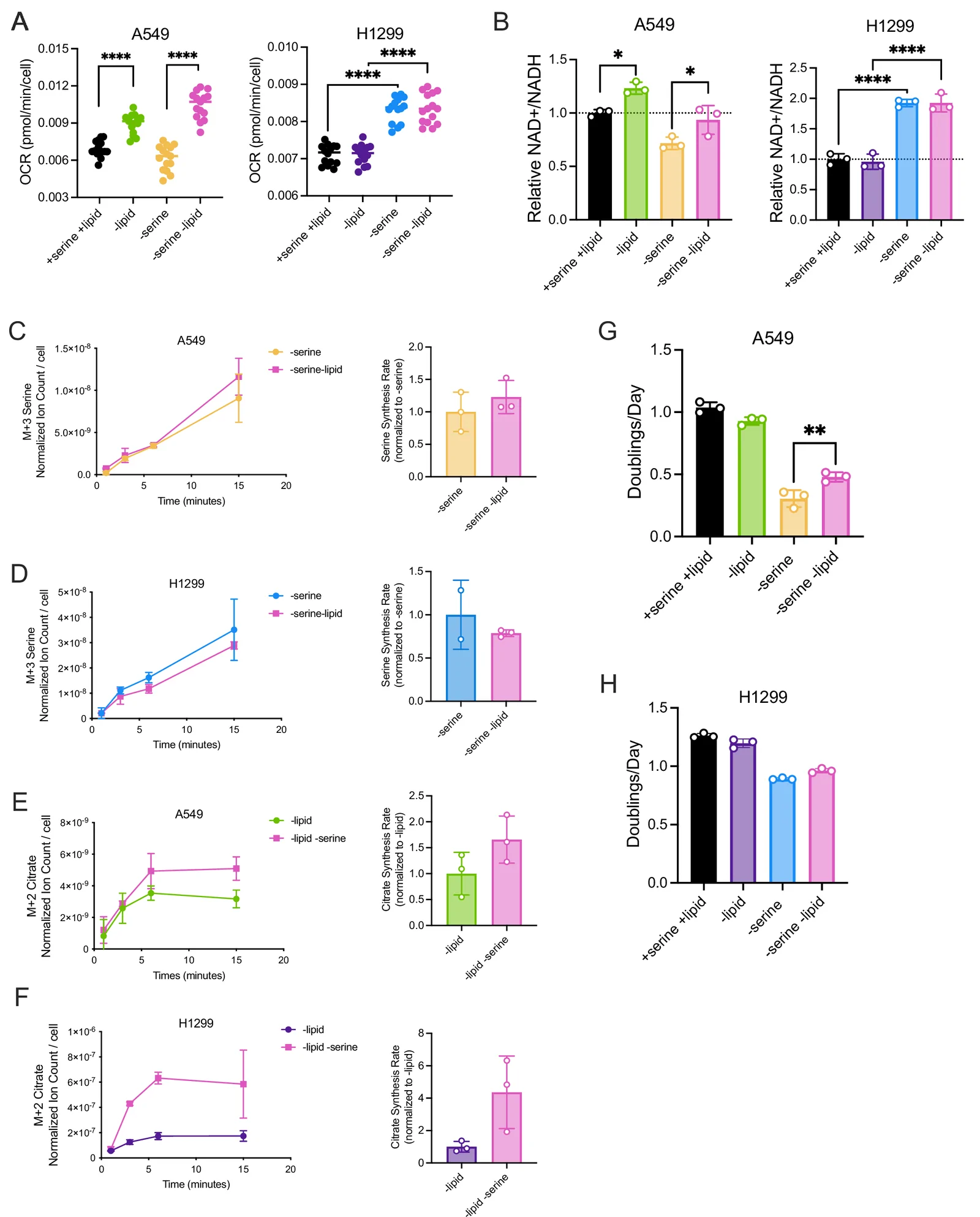
Lipid depletion increases mitochondrial respiration and the NAD+/NADH ratio in a cell-specific manner to influence serine synthesis and proliferation in serine-depleted conditions. (**A**) Oxygen consumption rate (OCR) of A549 and H1299 cells cultured with or without serine or lipids for 24 hr. Values are the average of three repeat measurements, n=14–15. p-Values were calculated by one-way ANOVA followed by post-hoc Tukey HSD test, ****p<0.001. (**B**) Relative NAD+/NADH ratios of A549 and H1299 cells cultured with or without serine or lipids for 24 hr. NAD+/NADH ratio is normalized to the NAD+/NADH ratio in corresponding cells cultured with serine and lipids, n=3. p-Values were calculated by one-way ANOVA followed by post-hoc Tukey HSD test, *p<0.05, ****p<0.001. (**C, D**) Left: Serine labeled (M+3) from U-^13^C-glucose over time in A549 cells (**C**) and H1299 cells (**D**) cultured without serine and with or without lipids for 24 hr prior to U-^13^C-glucose exposure. Right: Serine synthesis rates of A549 cells (**C**) and H1299 cells (**D**) cultured in indicated conditions. Serine synthesis rates are calculated from labeled serine (M+3) after 1 and 15 min of U-^13^C-glucose exposure and normalized to the serine synthesis rates of the corresponding cell line cultured without serine and with lipids. Serine levels are normalized to internal norvaline standard and cell number measured in indicated conditions, n=3. Single data point (H1299, -serine+lipid, t=15 min) removed due to detection error with no signal. (**E,F**) Left: Citrate labeled (M+2) from U-^13^C-glucose over time in A549 cells (**E**) and H1299 cells (**F**) cultured without lipids and with or without serine for 24 hr prior to U-^13^C-glucose exposure. Right: Citrate synthesis rates of A549 cells (**E**) and H1299 cells (**F**) cultured in indicated conditions. Citrate synthesis rates are calculated from labeled citrate (M+2) after 1 and 15 min of U-^13^C-glucose exposure and normalized to the citrate synthesis rates of the corresponding cell line cultured without lipids and with serine. Citrate levels are normalized to internal norvaline standard and cell number measured in indicated conditions, n=3. (**G**) Proliferation rate (doublings per day) of A549 cells cultured with or without serine or lipids for 72 hr, n=3. p-Values were calculated by one-way ANOVA followed by post-hoc Tukey HSD test, **p<0.01 (**H**) Proliferation rate (doublings per day) of H1299 cells cultured with or without serine or lipids for 72 hr, n=3. Data shown for all panels are means ± SD. Figure 6—source data 1.Excel file for the data in Figure 6A–H.

**Figure 7.**
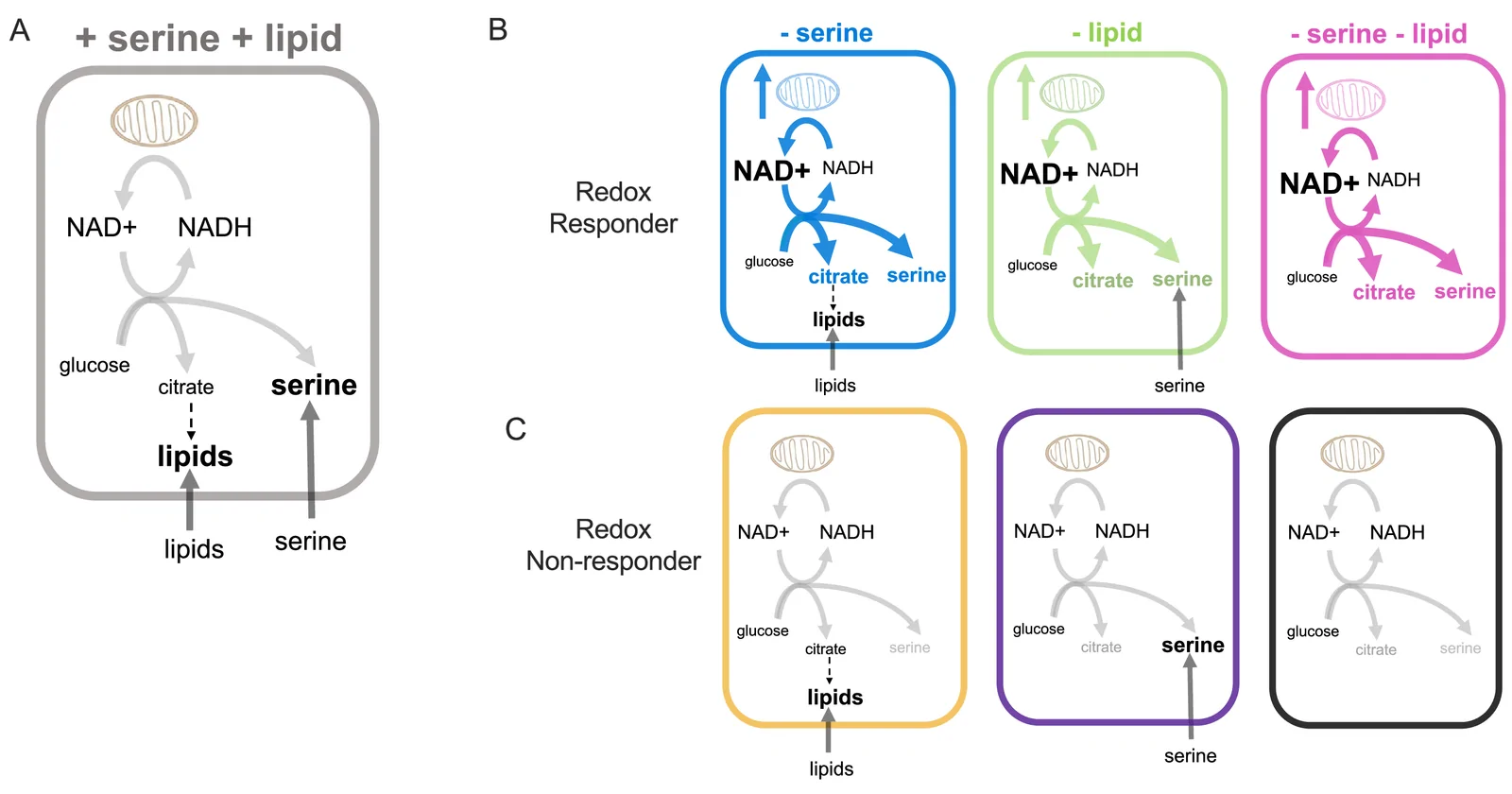
The cell NAD+/NADH ratio is regulated in a cancer cell-specific manner by changes to mitochondrial respiration in response to nutrient availability, impacting oxidative biosynthetic reactions. (**A**) In serine and lipid-replete conditions, cells can acquire environmental serine and lipids to support proliferation. Cells can also oxidize glucose to produce serine, as well as citrate for lipid synthesis. The oxidation reactions involved can be limited by NAD+ availability, which is impacted by mitochondrial respiration. (**B**) In nutrient-depleted conditions, the ability to support oxidative reactions is dependent on the mitochondrial response to the nutrient depletion. For redox responders, environmental depletion of serine or lipids increases the cell NAD+/NADH ratio to support both oxidative citrate and serine synthesis. This ability to increase respiration enables proliferation in conditions lacking serine. (**C**) In redox non-responder cells, neither mitochondrial respiration nor the NAD+/NADH ratio increases following nutrient depletion. This limits oxidative synthesis of serine (and citrate), subsequently limiting proliferation in environments that lack serine.

Our finding that both serine and oxidative citrate synthesis can be impacted by levels of a different nutrient than that produced by each pathway raised the possibility that the NAD+/NADH ratio coordinates biomass synthesis in response to the availability of multiple oxidized nutrients. Specifically, we hypothesized that changes to the NAD+/NADH ratio caused by one oxidized nutrient depletion could influence the synthesis of, and in turn the dependency on, another oxidized nutrient for proliferation. To begin testing this, we first examined how mitochondrial respiration and the NAD+/NADH ratio were impacted by dual lipid and serine depletion compared to single nutrient depletions in both A549 and H1299 cells. We find that dual depletion of serine and lipids in A549 cells led to elevated oxygen consumption that is higher than that observed with serine depletion alone (Figure 6A). Similarly, dual depletion of serine and lipids in H1299 cells led to elevated oxygen consumption compared to that observed with lipid depletion alone (Figure 6A). The resulting OCR corresponded with the cell NAD+/NADH ratio in each condition, which was collectively higher upon dual serine and lipid starvation than either serine depletion in A549 cells or lipid depletion in H1299 cells (Figure 6B). We then measured the serine and citrate synthesis rates of cells depleted of both extracellular serine and lipids via kinetic U-^13^C-glucose tracing. Consistent with the elevation of the NAD+/NADH ratio after lipid deprivation, A549 cells depleted of both environmental serine and lipids trended toward elevated serine synthesis rates when compared to serine deprivation alone, although it was not statistically significant (Figure 6C). Moreover, lipid depletion appeared to lead to a greater fraction of total serine derived from glucose in serine-depleted A549 cells (Figure 6—figure supplement 1A). In contrast, lipid starvation did not alter serine synthesis in serine-deprived H1299 cells (Figure 6D, Figure 6—figure supplement 1A,B). Interestingly, in both A549 and H1299 cells, dual serine and lipid deprivation led to a trend toward increased citrate production compared to lipid deprivation alone despite only H1299 cells exhibiting a more oxidized NAD+/NADH upon serine depletion (Figure 6E and F; Figure 6—figure supplement 1C,D). This underscores the multifactorial regulation of glucose oxidation to citrate (Holness and Sugden, 2003; Novelli and Lipmann, 1950; Patel and Korotchkina, 2006; Srere, 1974). We next asked how cancer cell proliferation was impacted by dual serine and lipid depletion compared to single nutrient depletions. Strikingly, proliferation of serine-depleted A549 cells was improved with depletion of extracellular lipids (Figure 6G), consistent with the elevation in mitochondrial respiration, NAD+/NADH ratio, and a trend toward greater serine synthesis in A549 cells upon dual serine and lipid depletion. Unlike serine depletion, lipid depletion does not cause a major proliferation defect in either A549 or H1299 cells, and dual serine and lipid deprivation did not greatly alter the proliferation of H1299 cells compared to serine depletion alone (Figure 6H). This is consistent with lipid deprivation causing no major changes in oxygen consumption, NAD+/NADH ratio, and serine synthesis in H1299 cells. These data argue that the cell NAD+/NADH ratio is modulated in response to multiple nutrient availabilities in a cancer cell-specific fashion. The changes in NAD+/NADH ratio in turn may influence the capacity for serine and citrate synthesis, impacting the proliferation rate of cancer cells in nutrient environments with differing demands for oxidative biomass synthesis. Taken together, we propose a model where environmental nutrient availability can impact mitochondrial respiration based on the specific cancer. Because mitochondrial respiration is a major pathway that regenerates NAD^+^, changes to mitochondrial respiration can alter the cell NAD+/NADH ratio, influencing the activity of major NAD^+^-requiring metabolic reactions such as serine synthesis and citrate synthesis that can be important for proliferation. We further propose that changes to the cell NAD+/NADH ratio can impact all oxidative biosynthetic reactions if the enzyme machinery is present, but that specificity for how the cell NAD+/NADH ratio changes is dependent on both cell-intrinsic and cell-extrinsic factors (Figure 7).

**Figure 6—figure supplement 1.**
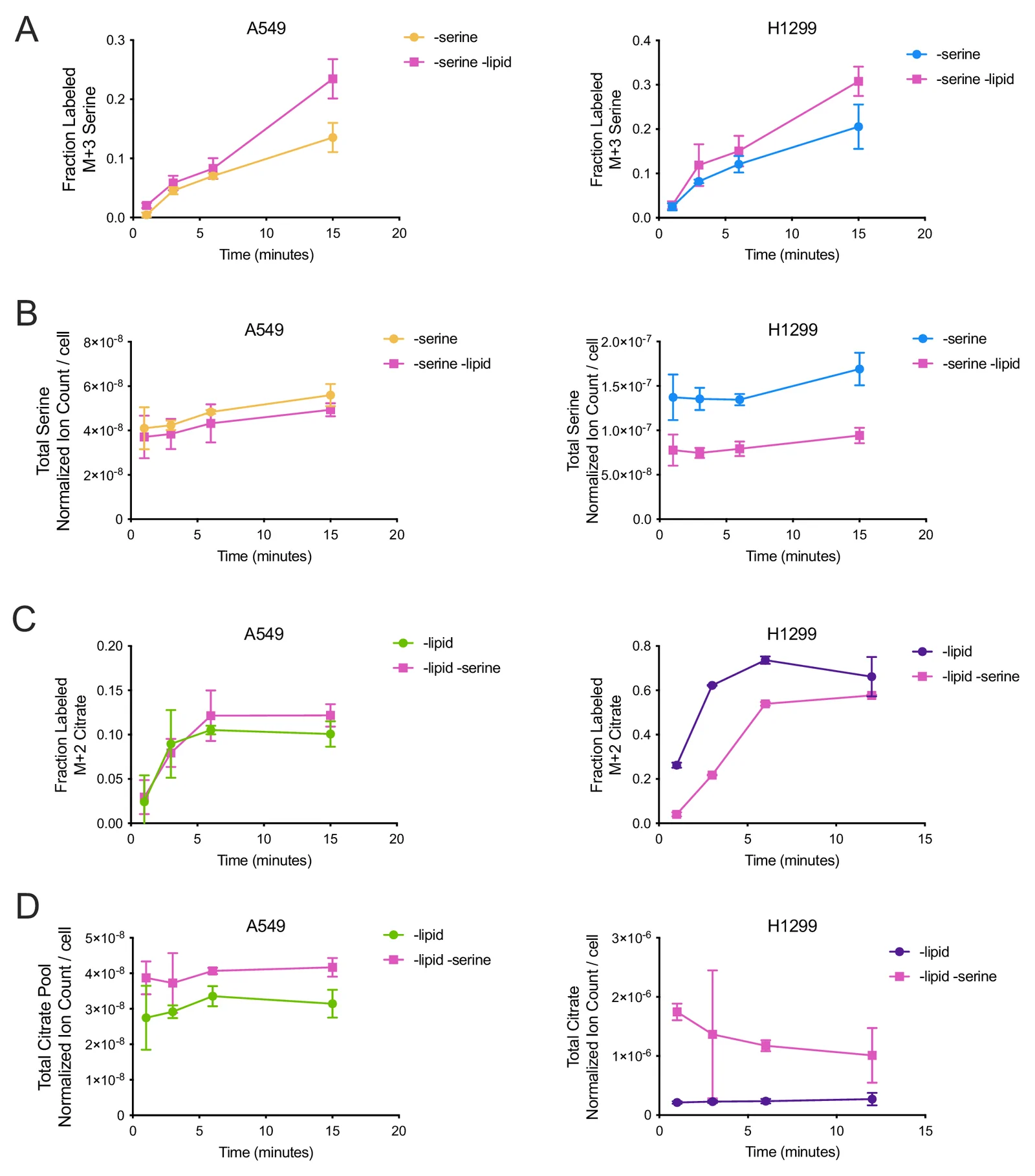
Kinetic tracing of labeled carbon from U-^13^C-glucose into serine and citrate in cells cultured in the absence of serine, lipids, or both serine and lipids. (**A**) Fraction of serine labeled (M+3) from U-^13^C-glucose over time in A549 and H1299 cells cultured without serine, and with or without lipids for 24 hr as indicated, n=3. (**B**) Total serine levels in A549 and H1299 cells cultured without serine, and with or without lipids for 24 hr for each time point shown in (**A**), n=3. (**C**) Fraction of citrate labeled (M+2) from U-^13^C-glucose over time in A549 and H1299 cells cultured without lipids, and with or without serine for 24 hr, as indicated, n=3. (**D**) Total citrate levels in A549 and H1299 cells cultured without lipids, and with or without serine for 24 hr for each time point shown in (**C**), n=3. All citrate and serine measurements were normalized to internal norvaline standard and cell number. Data shown for all panels are means ± SD. Figure 6—figure supplement 1—source data 1.Excel file for the data in Figure 6—figure supplement 1A–D.

## Discussion

Proliferating cancer cells must acquire sufficient biomass and rely on contributions from both environmental nutrients and de novo synthesis pathways, but what determines which cells can adapt to different nutrient environments that impose constraints on salvage and synthesis is not known. This study highlights that differential cell responses to changes in the NAD+/NADH ratio enable biomass synthesis and determine whether cells can adapt to proliferate in different nutrient conditions. Importantly, this provides one explanation for why single nutrient levels in tissues may not predict the ability of auxotrophy for that nutrient to determine cancer proliferation in a tissue (Abbott et al., 2026). It also suggests why cancer proliferation in different nutrient environments is controlled by a complex relationship between cell-intrinsic and cell-extrinsic factors. These factors may be coordinated in part through processes that set the NAD+/NADH ratio in cells.

Most prior work to determine what shapes the metabolic dependencies of cell proliferation has focused on factors that govern biosynthetic enzyme expression and activity. For example, genomic and transcriptional analyses of cancer discovered that increased PHGDH expression promotes tumor growth (DeNicola et al., 2015; Locasale et al., 2011; Newman and Maddocks, 2017; Possemato et al., 2011) and provides a proliferative advantage in serine-depleted tumor microenvironments (Ngo et al., 2020; Sullivan et al., 2019b). Our findings show that enzyme expression alone cannot fully explain nutrient dependencies, as PHGDH expression can be discordant with sensitivity to serine depletion. Rather, both the NAD+/NADH ratio and enzyme expression play integral roles in determining cancer proliferation in specific nutrient environments. These data are consistent with biosynthetic gene expression and single nutrient levels being unable to predict the tumor tissue environments where cancer cells can form metastases (Abbott et al., 2026).

Extensive work has highlighted the importance of cell NAD+/NADH ratio in influencing oxidized biomass production, nutrient utilization, and nutrient dependencies (Baksh et al., 2020; Bao et al., 2016; Birsoy et al., 2015; Broeks et al., 2023; Christensen et al., 2014; Diehl et al., 2019; Garcia-Bermudez et al., 2018; Gibson and Blass, 1976; Krall et al., 2021; Li et al., 2022; Sullivan et al., 2015; Wu et al., 2024). Yet, the processes that govern the endogenous NAD+/NADH ratio upon shifts in nutrient availability have been largely uncharacterized. We find that regulation of mitochondrial respiration is a major mechanism that dictates the NAD+/NADH ratio in response to environmental nutrient fluctuations. The mitochondrial respiration response to withdrawal of specific nutrients appears to vary across different cancer cells. Some cancer cells increased mitochondrial respiration to serine depletion, while others had minimal responses. We found that in serine redox responder cancer cells, the MAS enzymes MDH1 and GOT2 were required for the elevation in mitochondrial respiration and the NAD+/NADH ratio in response to serine depletion. Thus, differences in the activity of MAS, which can vary greatly across cancer cells (Wang et al., 2022), may influence the capacity for cancer cells to increase mitochondrial respiration and NAD+ regeneration to support oxidative biosynthetic pathways. Interestingly, serine is an allosteric activator of the glycolytic enzyme pyruvate kinase, which converts phosphoenolpyruvate to pyruvate and generates ATP (Chaneton et al., 2012). Thus, decreased environmental serine availability, in addition to differences in pyruvate kinase activity, may yield lower glycolytic ATP, resulting in greater mitochondrial respiration in serine redox responder cancer cells. Additionally, it has been recently shown that differences in mtDNA in non-small cell lung cancer cells can lead to defective mitochondrial respiration and imbalanced NAD+/NADH ratios, suppressing serine synthesis and tumor growth (Cararo Lopes et al., 2025). These findings suggest some reasons why respiration may be differentially responsive to serine withdrawal. However, lipid depletion yielded mitochondrial respiration changes in cancer cells that did not respond to serine withdrawal, arguing against respiration defects explaining non-response. No clear common genetic factor or tissue of origin was found to align with those cells that do or do not have mitochondrial and redox responses to serine and/or lipid depletion. Further work is needed to understand the mechanisms that enable increased mitochondrial respiration in response to deprivation of different nutrients. One possibility is that differences in ATP consumption following nutrient deprivation contribute to the response. Stimulating ATP consumption can increase mitochondrial respiration and the NAD+/NADH ratio (Bertholet et al., 2019; Buttgereit and Brand, 1995; Fisher-Wellman and Neufer, 2012; Luengo et al., 2021; Nobes et al., 1989; Vander Heiden et al., 1999). ATP synthase activity can restrain complex I activity to different extents across cancers, modulating the endogenous NAD+/NADH ratio and sensitivity to serine or lipid depletion. While the regulation of complex I, ATP synthesis, and mitochondrial coupling efficiency is complicated and incompletely understood (Alavian et al., 2014; Gao et al., 2018; Lapuente-Brun et al., 2013; Luengo et al., 2021; Qiu et al., 2014; Wang et al., 2022; Yao et al., 2019; Zhao et al., 2019), our findings suggest an important connection between the regulation of mitochondrial bioenergetics and specific metabolic vulnerabilities of cancer growth, underscoring an important relationship between environmental nutrient levels and mitochondrial respiration to influence the cell NAD+/NADH ratio.

Efforts to understand the processes that shape cancer cell metabolic dependencies have largely focused on how cancer cells respond to single nutrient changes. However, findings from dual serine and lipid depletion experiments suggest that single nutrient availabilities will be unable to explain cancer metabolic dependencies in some contexts. For example, depleting A549 cells of both extracellular serine and lipids paradoxically improved proliferation relative to serine deprivation alone, consistent with an elevated NAD+/NADH ratio in response to lipid deprivation that can support greater serine synthesis. This suggests that the NAD+/NADH ratio can serve as a metabolic regulatory node that orchestrates biomass synthesis and integrates changes to multiple oxidized biomass components in the environment, providing a framework for understanding why cancer nutrient dependencies vary across tissue microenvironments (Abbott et al., 2026). Because oxidizing the cell redox state can increase flux through multiple pathways, the impact of cell redox state on biosynthesis may also explain why some cells lose enzyme expression in pathways like serine synthesis, as this could redirect limited resources toward biomass components that can otherwise be scavenged from the environment (Delage et al., 2010; Pavlova and Thompson, 2016; Rabinovich et al., 2015). Better understanding the mechanisms cells use to alter respiration and adjust the NAD+/NADH ratio in response to available nutrients could inform the complex interplay between cell-intrinsic and cell-extrinsic factors that determine cancer metabolic dependencies. This is particularly important to consider when targeting metabolism for cancer treatment. Many newer therapies targeting metabolism have not been successful in part because of metabolic plasticity to nutrient shifts (Amoedo et al., 2017; Fendt et al., 2020; Xiao et al., 2023). Co-targeting mitochondrial function limits metabolic adaptations and may also help predict the tissue nutrient conditions that result in pathway dependencies for specific cancers. Thus, better understanding how the cell NAD+/NADH ratio responds to nutrient levels in different cancers could improve selection of patients for cancer therapies that impact metabolism.

## Materials and methods

### Cell culture experiments

Cell lines were maintained in Dulbecco’s modified Eagle’s medium (DMEM) without sodium pyruvate (Corning 50–013-PC) supplemented with sodium bicarbonate (Sigma S6014) and 10% heat-inactivated FBS. All cells were cultured at 37 °C with 5% CO_2_, tested for mycoplasma contamination regularly, and confirmed negative before experimentation. Cell line information is listed in Supplementary file 1.

### Proliferation assays

Cells were plated in six-well plates in 2 ml of DMEM with 10% heat-inactivated FBS at an initial seeding density of 40,000 cells. Cells were permitted to settle overnight and then quantified using a sulforhodamine B (SRB; Sigma-Aldrich 230162) colorimetric assay to measure the starting cell absorbance at the start of the experiment as previously described (Vichai and Kirtikara, 2006). For treatment conditions, cells were washed three times with 1 ml of 1 X PBS, and 2 ml of treatment media were added to each well. All treatment media were made with 10% dialyzed FBS. Serine-free medium was made by adding a mix of amino acids (Sigma-Aldrich) at DMEM concentrations lacking serine and glycine to DMEM with low glucose, without sodium pyruvate, and amino acids (US Biological D9800-13-50L). Glucose and glycine were supplemented for a final concentration of 25 mM and 0.4 mM, respectively. Three days after the initial treatment, cells were quantified using the SRB assay. All SRB measurements are normalized to a blank to correct for background signal. Proliferation rates were calculated with the following formula:

$$\displaystyle {Doublings\ per\ day} = \frac{log_2\left({{final\ SRB\ absorbance}}\,/\,{{SRB\ absorbance\ day\ 0}}\right)} {{(total\ \#\ of\ treatment\ days)}}$$

### Cell number and viability measurements

Cells were seeded at an initial density of 2000 cells per well in a 96-well plate in 200 μl of treatment media. After allowing cells to settle for 1–2 hr, cell number was monitored over time using imaging with the Incucyte Live-Cell Analysis System (Sartorius). Doublings per day were calculated between hour 0 of monitoring and hour 72 of monitoring using the following formula:

$$\displaystyle {Doublings\ per\ day} = \frac{log_2\left(cell\ number\ at\ hour\ 72 \,/\, cell\ number\ at\ hour\ 0\right)} {{(3\ days\ of\ treatment)}}$$

For viability, cells were seeded at an initial density of 2000 cells per well in a 96-well plate in 200 μl of treatment media with 20 nM Sytox Green (Invitrogen S7020). After 72 hr, the number of live and dead cells present was monitored using imaging with the Incucyte Live-Cell Analysis System (Sartorius).

### NAD+/NADH measurements

NAD+/NADH measurements were performed using the NAD/NADH-Glo Assay (Promega G9072) with a modified version of manufacturer instructions as previously reported (Sullivan et al., 2015). Cells were plated at an initial seeding density of 20,000 cells (A549, H1299, HCT116) or 40,000 cells (Calu6, MDA-MB-231). Cells were permitted to settle overnight. The next day, cells were washed once with 1 X PBS and then incubated with 2 ml of treatment media for the indicated times prior to preparation of cell extracts. For extraction, cells were washed once in ice-cold 1 X PBS and extracted in 100 μl ice-cold 1% dodecyltrimethylammonium bromide (DTAB) in 0.2 N NaOH diluted 1:1 with 1 X PBS. Each sample was flash-frozen in liquid nitrogen and immediately stored at –80 °C. To measure NADH, 10 µl of sample was moved to PCR tubes, diluted with 10 µl of DTAB, and incubated at 75 °C for 30  min, where basic conditions selectively degrade NAD+. To measure NAD+, 10 µl of the samples was moved to PCR tubes containing 30 µl DTAB and 20 µl 0.4  N HCl and incubated at 60 °C for 15 min, where acidic conditions selectively degrade NADH. Samples were then allowed to equilibrate to room temperature and quenched by neutralizing with 20 µl 0.25 M Tris in 0.2 N HCl (for NADH) or 20 µl 0.5 M Tris base (for NAD+). Manufacturer instructions were followed thereafter to measure NAD+/NADH using a luminometer (Tecan Infinite M200Pro). A standard curve with representative samples was done with each assay to confirm that NAD+ and NADH measurements were in the linear range of detection.

### Glucose consumption and lactate secretion measurements

Cells were plated at a density of 40,000 cells per well in a six-well plate. The next day, cells were washed three times with 1 X PBS before 2 ml of treatment media were added to each well. In parallel, 2 ml of treatment media were added to plates without cells. An initial cell number before beginning treatment was quantified via Cellometer (Nexcelom Bioscience). 72 hr later, media was collected from exponentially proliferating cells with a parallel collection of media from wells without cells, which we used to measure initial media concentrations to take into consideration evaporative changes. Cell number was quantified via Cellometer along with the volume of media on cells to consider evaporative changes to metabolite concentrations that may have occurred over 72 hr in culture. Glucose concentrations from media samples were measured on a YSI-2900 Biochemistry Analyzer (Yellow Spring Instruments). Every assay included a glucose (Sigma Aldrich G8270) standard curve (0, 2.5, 5, 10, 20, and 40 mM glucose) and lactate (Sigma Aldrich 71716) standard curve (0, 1.25, 2.5, 5, 10, and 20 mM sodium lactate), made in both control and treatment media. To calculate consumption rate per cell for a given nutrient, fmol of glucose or lactate over time were plotted relative to the area under the curve (fmol / cells · time) of an exponential function fit to the number of cells at the start of the assay and after 72 hr of treatment, as previously described (Hosios et al., 2016).

### Oxygen consumption measurements

An Agilent Seahorse Bioscience Extracellular Flux Analyzer (XFe96) was used to measure OCR. Cells were plated at 20,000–40,000 cells per well in a Seahorse Bioscience 96-well plate in 80 μl of DMEM without pyruvate supplemented with 10% heat-inactivated FBS. Cells were not plated on the perimeter of the plate to avoid edge effect. The following day, cells were washed twice with 180 μl of treatment media supplemented with 10% dialyzed FBS before incubating cells with 180 μl of treatment media for the indicated time prior to OCR data acquisition. For 24 hr treatment periods, treatment media was replaced with 180 μl of fresh treatment media two hours before OCR data acquisition. The day before OCR data acquisition, the XFe96 cartridge was hydrated by submerging the sensor cartridge in 200 μl of sterile water per well of the utility plate. Parafilm was wrapped around the perimeter of the cartridge to avoid evaporation. The XFe96 cartridge was then incubated in a 37 °C non-CO_2_ incubator overnight. The next day, at least 2 hr before data acquisition, the water in the utility plate was replaced with 200 μl of the Agilent XF Calibrant per well and the sensory cartridge was submerged and returned to the 37 °C non-CO_2_ incubator until OCR was ready to be measured. After OCR data acquisition, six to eight wells per treatment condition were collected for cell number quantification (Cellometer) to normalize OCR values. Basal OCR was calculated by subtracting residual OCR, the OCR remaining following the addition of rotenone and antimycin A for a final concentration of 2 μM.

### Kinetic U-^13^C-glucose isotope tracing experiments

Cells were seeded at 150,000 cells per well in a six-well plate in 2 ml of DMEM without sodium pyruvate supplemented with 10% heat-inactivated FBS. The following day, cells were washed once with 1 ml of 1 X PBS and then incubated in 2 ml of treatment media supplemented with 10% dialyzed FBS for the indicated treatment time and when metabolic steady state is reached. Two hours before the start of the kinetic isotope tracing, media on cells was exchanged with fresh treatment media with 10 mM unlabeled glucose. To measure serine labeling over time in serine-depleted conditions, cells and media were extracted together. Before the initiation of the kinetic U-^13^C-glucose isotope tracing, serine-deplete cells were washed three times with 1 X PBS and then cultured with 400 μl of tracing medium containing 10 mM of U-^13^C-glucose (Cambridge Isotope Laboratories, CLM-481–0) for the rapid time points. Tracing media was equivalent to treatment media except with 10 mM of U-^13^C-glucose. Following incubation, 1.6 ml of extraction buffer containing ice cold 100% HPLC-grade methanol (Sigma Aldrich, 646377) with norvaline (Sigma Aldrich, N7627) was added onto cells with tracing media for a final sample volume of 2 ml (80% extraction buffer and 20% tracing media) and a final concentration of 1  μg per 400  μl of norvaline. 1.6 ml of the lysate was placed into a fresh Eppendorf tube, vortexed at 4 °C at maximum speed for 10 min, and then spun down at 4 °C at maximum speed for 10 min. The supernatant was collected and dried under nitrogen gas to prepare for metabolic analysis. To measure serine and citrate production in serine replete conditions, only intracellular metabolites were quantified. Before the initiation of kinetic U-^13^C-glucose isotope tracing, serine-replete cells were washed three times with 1 X PBS and then cultured with 1 ml of tracing medium containing 10 mM of U-^13^C-glucose (Cambridge Isotope Laboratories, CLM-481–0) for the rapid time points. Tracing media was equivalent to treatment media except with 10 mM of U-^13^C-glucose. Following incubation, cells were rapidly washed twice with ice cold blood bank saline. To extract, 500 μl of extraction buffer containing 80% ice cold HPLC-grade methanol and 20% LC-grade water spiked with 1  μg per 400  μl norvaline were added onto cells. Cells were then lysed as previously described above.

### Kinetic U-^13^C-glutamine isotope tracing experiments

Cells were seeded at 150,000 cells per well in a six-well plate in 2 ml of DMEM without sodium pyruvate supplemented with 10% heat-inactivated FBS. The following day, cells were washed once with 1 ml of 1 X PBS and then incubated in 2 ml of treatment media supplemented with 10% dialyzed FBS for the indicated treatment time and when metabolic steady state is reached. Before the initiation of kinetic U-^13^C-glutamine tracing, cells were washed three times with 1 X PBS and then cultured in 2 ml of medium containing 2 mM U-^13^C-glutamine (Cambridge Isotope Laboratories, CLM-1822-H-0.5) for the indicated treatment conditions. Following incubation, cells were rapidly washed twice with ice cold blood bank saline. To extract, 500 μl of extraction buffer containing 80% ice-cold HPLC-grade methanol and 20% LC-grade water spiked with 1  μg per 400  μl norvaline were added onto cells. Cell lysates were then placed into a fresh Eppendorf tube, vortexed at 4 °C at maximum speed for 10 min, and then spun down at 4 °C at maximum speed for 10 min. The supernatant was collected and dried under nitrogen gas to prepare for metabolic analysis.

### Gas chromatography-mass spectrometry (GC-MS) polar metabolite measurements

Dried samples were derivatized by adding 16  μl of methoxamine reagent (Thermo Fisher, TS-45950) and incubated for an hour at 37 °C followed by the addition of 20  μl of *N-tert*-butyldimethylsilyl-*N*-methyltrifluoroacetamide with 1% *tert*-butyldimethylchlorosilane (Sigma-Aldrich, 375934). Samples were then incubated for 2 hr at 60 °C. Afterwards, samples were centrifuged at maximum speed for 10 min, and 20 μl of supernatant was used for analysis. Following derivatization, samples were analyzed using a DB-35MS column (30  m × 0.25  mm i.d. × 0.25  μm, Agilent J&W Scientific) in an Agilent 7890 gas chromatograph coupled to an Agilent 5975 C mass spectrometer (GC–MS). All ion counts were normalized to internal standard norvaline and cell number measured via Cellometer.

### Immunoblotting

Cells were washed with ice-cold 1 X PBS and lysed in cold RIPA buffer containing Halt Protease and Phosphatase Inhibitor Cocktail (Thermo Scientific, 78442). Lysates were clarified by rocking samples for thirty minutes at 4 °C and then spun at maximum speed for ten minutes for collection of supernatants. Protein concentration was calculated using the BCA Protein Assay (Pierce, 23225) with BSA as a standard. Lysates were resolved by SDS-PAGE using NuPAGE 4–12% Bis-Tris Protein Gels (Thermo Fisher Scientific) and run at 100 V. Proteins were transferred onto nitrocellulose membranes via wet transfer at 100 V. Membranes were blocked in 5% BSA in 1 x TBST before incubating membranes with primary antibodies at 4 °C overnight. The next day, membranes were washed three times with 1 x TBST at room temperature and then incubated in secondary antibodies for one hour at room temperature. The primary antibodies used were PHGDH (Sigma Aldrich, HPA021241 [1:1000]), PSAT1 (Abcam, ab96136 [1:1000]), PSPH (Abcam, ab96414 [1:1000]), HSP90 (Cell Signaling Technology, 4874 [1:1000]), Vinculin (E1E9V) XP (Cell Signaling Technology, 13901 [1:1000]), GOT1 (Cell Signaling Technology, 34423 S [1:1000]), MDH1 (MDHC H-6, Santa Cruz Biotechnology, sc-166879 [1:1000]), GOT2 (Cell Signaling Technology, 71692 S [1:1000]), FLAG (Cell Signaling Technology, 14793 [1:1000]). The secondary antibodies used were anti-rabbit IgG horseradish peroxidase-linked antibody (1:4000 dilution; Cell Signaling Technologies, 7074 S) or anti-mouse IgG horseradish peroxidase-linked antibody (1:4000, 7076 S). Protein expression was quantified by using ImageJ analysis.

### Genetic manipulation of enzyme expression in cells

Cell lines overexpressing either EGFP or human PHGDH were generated via lentiviral infection. pLJM1-EGFP was obtained from Addgene (Addgene 19319). pLJM1-PHGDH was constructed using pLJM1-Empty (Addgene 91980) as a backbone. pLJM1-Empty was digested using NheI and EcoRI and gel purified. Human PHGDH cDNA was amplified from pLHCX-PHGDH (PMID: 31598584) with the following oligonucleotide primers, where the capitalized sequence denotes PHGDH homology:

PHGDH F: agtgaaccgtcagatccggctagcgccaccATGGCTTTTGCAAATCTGCG

PHGDH R: tactgccatttgtctcgaggtcgagaattcTTAGAAGTGGAACTGGAAGGCT

PHGDH insert was gradient PCR amplified using Phusion High-Fidelity DNA polymerase (NEB M0530) from 60 to 70°C and gel purified. Digested pLJM1-Empty and amplified PHGDH insert were assembled via Gibson Assembly, and the resulting plasmid was transformed and validated by Sanger sequencing. Lentiviral production was done by transfecting constructs into LentiX293T cells with Mirus Transit293T (Mirus Bio MIR2700) following the manufacturer’s protocol. Briefly, 800,000 cells were plated per well in a six-well plate in DMEM media with 10% heat-inactivated FBS. The next day, cells were transfected with 1.6 μg vector (either pLJM1-EGFP or pLJM1-PHGDH), 800 ng of pMDLg (Addgene 12251) packaging plasmid, 400 ng of pMD2.G (Addgene 12259) envelope plasmid, and 400 ng of pRSV-REV (Addgene 12253) in OptiMEM (Thermo Fisher Scientific 31985070). DNA transfection mix was incubated with cells for 48 hr before harvesting virus and filtering through a 0.45 μm polyethersulfone membrane. Sub-confluent cells were infected with 1 ml of virus-containing media in each well of a six-well plate with polybrene (8 μg/ml, EMD Millipore TR-1003-G). 24 hr after infection, selection with 1 μg/ml puromycin (Sigma Aldrich P7255) began. All experiments with generated cell lines expressing PHGDH were conducted on polyclonal cell populations.

cyto*Lb*NOX-FLAG cDNA and mito*Lb*NOX-FLAG cDNA were PCR amplified from pUC57-*Lb*NOX or pUC57-mito*Lb*NOX, respectively. pUC57-*Lb*NOX (Addgene 75285) and pUC57-mito*Lb*NOX (Addgene 74448) were a gift from Vamsi Mootha. The PCR products were subsequently cloned into pLJM1 using Gibson Assembly (NEB E2611S). pLJM1-EGFP was a gift from David Sabatini (Addgene 19319). Cells were then transduced with lentivirus containing pLJM1-*Lb*NOX-FLAG, pLJM1-mito*Lb*NOX-FLAG, or pLJM1-EGFP. The infected cells were selected in 1 μg/ml puromycin (Sigma Aldrich P7255). All experiments with generated cell lines involving *Lb*NOX were conducted on a polyclonal cell population.

GOT1, GOT2, and MDH1 Knockout H1299 cells were generated by using lentiCRISPRv2 (Addgene 52961). LentiCRISPRv2 was a gift from Feng Zhang. Guide RNAs (gRNAs) for each gene of interest were designed by CRISPick (https://portals.broadinstitute.org/gppx/crispick/public) and cloned into lentiCRISPRv2. The guide sequences used are as follows:

NTC: GTGTAGTTCGACCATTCGTG
GOT1: GGAGGTGTGCAATCTTTGGG
GOT2: CGGACGCGGGTCCACTCCCG
MDH1: CGTCCAGGACACCCATCATG

Cell lines were generated by transducing cells with viral particles containing lentiCRISPRv2 followed by selection with 1  µg/ml puromycin (Sigma Aldrich P7255). Single cells were plated into individual wells of 96-well plates in RPMI medium to obtain single clone knockout cells. Following expansion, immunoblotting was used to confirm complete knockout.

### Statistics and reproducibility

All statistical tests using experimental data were performed with Prism 10 software. No statistical method was used to predetermine sample sizes. Sample sizes were chosen based on pilot experiments using three or more technical replicates. The experiments were not randomized, and the investigators were not blinded during experiments nor data analysis.

## Data Availability

All data used to generate figures are provided with the source data.

## Additional files

Supplementary file 1. Genetic, tissue, and source information for the studied cancer cell lines.

MDAR checklist

## Appendix 1

**Appendix 1—key resources table:**

Reagent type (species) or resource	Designation	Source or reference	Identifiers	Additional information
Cell line (*Homo sapiens*)	Lung adenocarcinoma	ATCC	Calu-6 RRID:CVCL_0236	
Cell line (*Homo sapiens*)	Lung adenocarcinoma	ATCC	A549 RRID:CVCL_0023	
Cell line (*Homo sapiens*)	Pancreatic ductal adenocarcinoma	DSMZ	PaTu 8988T RRID:CVCL_1847	
Cell line (*Homo sapiens*)	Pancreatic ductal adenocarcinoma	ATCC	MIA PaCa-2 RRID:CVCL_0428	
Cell line (*Homo sapiens*)	Lung adenocarcinoma	ATCC	H1299 RRID:CVCL_0060	
Cell line (*Homo sapiens*)	Colon carcinoma	ATCC	HCT116 RRID:CVCL_0291	
Cell line (*Homo sapiens*)	Malignant melanoma	ATCC	A375 RRID:CVCL_IW10	
Cell line (*Homo sapiens*)	Breast adenocarcinoma	ATCC	MDA-MB-231 RRID:CVCL_IW10	
Cell line (*Homo sapiens*)	Breast adenocarcinoma	ATCC	MCF7 RRID:CVCL_0031	
Chemical compound	Sulforhodamine B (SRB)	Sigma Aldrich	230162	0.57% in 1% acetic acid
Chemical compound	Trichloroacetic acid	Sigma Aldrich	T9159	10%
Chemical compound	DMEM low glucose, without amino acids and pyruvate	US Biological	D9800-13-50L	
Chemical compound	Sytox Green	Invitrogen	S7020	20 nM
Commercial Assay	NAD/NADH-Glo Assay	Promega	G9072	
Chemical compound	dodecyltrimethylammonium bromide (DTAB)	Sigma Aldrich	D8638	1% in 0.2 N NaOH diluted 1:1 with 1 X Phosphate Buffered Saline
Chemical compound	Sodium alpha-ketobutyrate	Sigma Aldrich	K0875	
Chemical compound, drug	rotenone	Sigma Aldrich	R8875	
Chemical compound, drug	Antimycin A	Sigma Aldrich	A8674	
Chemical compound, drug	FCCP	Sigma Aldrich	C2920	
Chemical compound, drug	Oligomycin A	Sigma Aldrich	75351	
Chemical compound, drug	FK866	MedChem Express	HY-50876	
Chemical compound	Nicotinamide mononucleotide	Sigma Aldrich	N3501	
Chemical compound	Uniformly labelled ^13^C-glucose (U-^13^C-glucose)	Cambridge Isotope Laboratories	CLM-481–0	10 mM
Chemical compound	Uniformly labelled ^13^C-glutamine (U-^13^C-glutamine)	Cambridge Isotope Laboratories	CLM-1822-H-0.5	2 mM
Chemical compound	HPLC-grade methanol	Sigma Aldrich	646377	
Chemical compound	L-Norvaline	Sigma Aldrich	N7627	1 μg per 400 μl extraction buffer
Chemical compound	Methoxamine	Thermo Fisher	TS-45950	
Chemical compound	N-tert-butyldimethylsilyl-N-methyltrifluoroacetamide with 1% tert-butyldimethylchlorosilane	Sigma Aldrich	375934	
Other	RIPA Buffer	Boston BioProducts	BP-115–250	Protein lysis buffer for western blot analysis; see Methods: Immunoblotting
Other	Halt Protease and Phosphatase Inhibitor Cocktail	Thermo Scientific	78442	Protease and phosphatase inhibitor added to the lysis buffer; see Methods: Immunoblotting
Commercial Kit	Pierce BCA Protein Assay	Thermo Scientific	23225	
Other	NuPAGE LDS Sample Buffer (4 X)	Invitrogen	NP0007	Sample loading buffer for SDS-PAGE; see Methods: Immunoblotting
Antibody	Anti-PHGDH (rabbit polyclonal)	Sigma Aldrich	HPA021241 RRID:AB_1855299	1:1000
Antibody	Anti-PSAT1 (rabbit polyclonal)	Abcam	ab96136 RRID:AB_10697763	1:1000
Antibody	Anti-PSPH (rabbit polyclonal)	Abcam	ab96414 RRID:AB_10678573	1:1000
Antibody	Anti-HSP90 (rabbit polyclonal)	Cell Signaling Technology	4874 RRID:AB_2121214	1:1000
Antibody	Anti-Vinculin (rabbit monoclonal)	Cell Signaling Technology	13901 RRID:AB_2728768	1:1000
Antibody	Anti-GOT1 (rabbit monoclonal)	Cell Signaling Technology	34423 S RRID:AB_2799052	1:1000
Antibody	Anti-MDHC (mouse monoclonal)	Santa Cruz Biotechnology	sc-166879 RRID:AB_10609257	1:1000
Antibody	Anti-GOT2 (rabbit monoclonal)	Cell Signaling Technology	71692 S RRID:AB_3662816	1:1000
Antibody	Anti-FLAG (rabbit monoclonal)	Cell Signaling Technology	14793 RRID:AB_2572291	1:1000
Antibody	Anti-rabbit IgG horseradish peroxidase-linked antibody (goat)	Cell Signaling Technology	7074 S RRID:AB_2099233	1:4000
Antibody	Anti-mouse IgG horseradish peroxidase-linked antibody (horse)	Cell Signaling Technology	7076 S RRID:AB_330924	1:4000
Recombinant DNA reagent	pLJM1-EGFP (plasmid)	Addgene	19319 RRID:Addgene_19319	
Recombinant DNA reagent	pLHCX-PHGDH (plasmid)	PMID 31598584		Used to amplify human PHGDH cDNA
Sequence-based reagent	PHGDH F	This paper	PCR primer	agtgaaccgt Cagatccggct Agcgccacc ATGGCTTTT GCAAATCTG CG
Sequence-based reagent	PHGDH R	This paper	PCR primer	Tactgccattt Gtctcgaggtc gagaattcTT AGAAGTGG AACTGGAAGGCT
Other	Phusion High-Fidelity DNA polymerase	NEB	M0530	High-fidelity DNA polymerase used for PCR amplification of PHGDH cDNA; see Methods: Genetic Manipulation of Enzyme Expression in Cells
Other	Mirus Transit293T	Mirus Bio	MIR2700	Transfection reagent used for lentiviral production in LentiX293T cells; see Methods: Genetic Manipulation of Enzyme Expression in Cells
Recombinant DNA reagent	pUC57-LbNOX (plasmid)	Addgene	75285 RRID:Addgene_75285	
Recombinant DNA reagent	pUC57-mitoLbNOX (plasmid)	Addgene	74448 RRID:Addgene_74448	
Commercial kit	Gibson Assembly Master Mix	NEB	E2611S	
Recombinant DNA reagent	lentiCRISPRv2 (plasmid)	Addgene	52961 RRID:Addgene_52961	
Sequence-based reagent	NTC gRNA	This paper		GTGTAGTT CGACCATTCGTG
Sequence-based reagent	GOT1 gRNA	This paper		GGAGGTG TGCAATCTTTGGG
Sequence-based reagent	GOT2 gRNA	This paper		CGGACGCG GGTCCACTCCCG
Sequence-based reagent	MDH1 gRNA	This paper		CGTCCAG GACACCCATCATG

## References

[bib1] Abbott KL, Subudhi S, Ferreira R, Gültekin Y, Steinbuch SC, Munim MB, Ramesh DL, Honeder SE, Kumar AS, Wu M, Hansen JA, Shevzov-Zebrun A, Rashan EH, Eghbalian KM, Sivanand S, Barbeau AM, Riedmayr LM, Duquette M, Ali A, Henning N, Trojan SE, Waite M, Kunchok T, Nakano MA, Gourgue F, Ferraro GB, Do BT, Spanoudaki V, Sánchez-Rivera FJ, Jin X, Church GM, Jain RK, Vander Heiden MG (2026). Nutrient requirements of organ-specific metastasis in breast cancer. Nature.

[bib2] Alavian KN, Beutner G, Lazrove E, Sacchetti S, Park HA, Licznerski P, Li H, Nabili P, Hockensmith K, Graham M, Porter GA, Jonas EA (2014). An uncoupling channel within the c-subunit ring of the F1FO ATP synthase is the mitochondrial permeability transition pore. PNAS.

[bib3] Amoedo ND, Obre E, Rossignol R (2017). Drug discovery strategies in the field of tumor energy metabolism: limitations by metabolic flexibility and metabolic resistance to chemotherapy. Biochimica et Biophysica Acta. Bioenergetics.

[bib4] Apiz Saab JJ, Dzierozynski LN, Jonker PB, AminiTabrizi R, Shah H, Menjivar RE, Scott AJ, Nwosu ZC, Zhu Z, Chen RN, Oh M, Sheehan C, Wahl DR, Pasca di Magliano M, Lyssiotis CA, Macleod KF, Weber CR, Muir A (2023). Pancreatic tumors exhibit myeloid-driven amino acid stress and upregulate arginine biosynthesis. eLife.

[bib5] Baksh SC, Todorova PK, Gur-Cohen S, Hurwitz B, Ge Y, Novak JSS, Tierney MT, Dela Cruz-Racelis J, Fuchs E, Finley LWS (2020). Extracellular serine controls epidermal stem cell fate and tumour initiation. Nature Cell Biology.

[bib6] Banh RS, Biancur DE, Yamamoto K, Sohn ASW, Walters B, Kuljanin M, Gikandi A, Wang H, Mancias JD, Schneider RJ, Pacold ME, Kimmelman AC (2020). Neurons release serine to support mRNA translation in pancreatic cancer. Cell.

[bib7] Bao XR, Ong SE, Goldberger O, Peng J, Sharma R, Thompson DA, Vafai SB, Cox AG, Marutani E, Ichinose F, Goessling W, Regev A, Carr SA, Clish CB, Mootha VK (2016). Mitochondrial dysfunction remodels one-carbon metabolism in human cells. eLife.

[bib8] Bertholet AM, Chouchani ET, Kazak L, Angelin A, Fedorenko A, Long JZ, Vidoni S, Garrity R, Cho J, Terada N, Wallace DC, Spiegelman BM, Kirichok Y (2019). H^+^ transport is an integral function of the mitochondrial ADP/ATP carrier. Nature.

[bib9] Birsoy K, Wang T, Chen WW, Freinkman E, Abu-Remaileh M, Sabatini DM (2015). An essential role of the mitochondrial electron transport chain in cell proliferation is to enable aspartate synthesis. Cell.

[bib10] Broeks MH, Meijer NWF, Westland D, Bosma M, Gerrits J, German HM, Ciapaite J, van Karnebeek CDM, Wanders RJA, Zwartkruis FJT, Verhoeven-Duif NM, Jans JJM (2023). The malate-aspartate shuttle is important for de novo serine biosynthesis. Cell Reports.

[bib11] Buttgereit F, Brand MD (1995). A hierarchy of ATP-consuming processes in mammalian cells. The Biochemical Journal.

[bib12] Cararo Lopes E, Shi F, Sawant A, Ibrahim M, Gomez-Jenkins M, Hu Z, Manchiraju P, Bhatt V, Wang W, Hinrichs CS, Wallace DC, Su X, Rabinowitz JD, Chan CS, Guo JY, Ganesan S, Lattime EC, White E (2025). Respiration defects limit serine synthesis required for lung cancer growth and survival. Nature Communications.

[bib13] Chaneton B, Hillmann P, Zheng L, Martin ACL, Maddocks ODK, Chokkathukalam A, Coyle JE, Jankevics A, Holding FP, Vousden KH, Frezza C, O’Reilly M, Gottlieb E (2012). Serine is a natural ligand and allosteric activator of pyruvate kinase M2. Nature.

[bib14] Christensen CE, Karlsson M, Winther JR, Jensen PR, Lerche MH (2014). Non-invasive in-cell determination of free cytosolic [NAD+]/[NADH] ratios using hyperpolarized glucose show large variations in metabolic phenotypes. The Journal of Biological Chemistry.

[bib15] Cognet G, Muir A (2024). Identifying metabolic limitations in the tumor microenvironment. Science Advances.

[bib16] DeBerardinis RJ, Lum JJ, Hatzivassiliou G, Thompson CB (2008). The biology of cancer: metabolic reprogramming fuels cell growth and proliferation. Cell Metabolism.

[bib17] Delage B, Fennell DA, Nicholson L, McNeish I, Lemoine NR, Crook T, Szlosarek PW (2010). Arginine deprivation and argininosuccinate synthetase expression in the treatment of cancer. International Journal of Cancer.

[bib18] DeNicola GM, Chen PH, Mullarky E, Sudderth JA, Hu Z, Wu D, Tang H, Xie Y, Asara JM, Huffman KE, Wistuba II, Minna JD, DeBerardinis RJ, Cantley LC (2015). NRF2 regulates serine biosynthesis in non-small cell lung cancer. Nature Genetics.

[bib19] Diehl FF, Lewis CA, Fiske BP, Vander Heiden MG (2019). Cellular redox state constrains serine synthesis and nucleotide production to impact cell proliferation. Nature Metabolism.

[bib20] Fantin VR, St-Pierre J, Leder P (2006). Attenuation of LDH-A expression uncovers a link between glycolysis, mitochondrial physiology, and tumor maintenance. Cancer Cell.

[bib21] Faubert B, Solmonson A, DeBerardinis RJ (2020). Metabolic reprogramming and cancer progression. Science.

[bib22] Fendt S-M, Frezza C, Erez A (2020). Targeting metabolic plasticity and flexibility dynamics for cancer therapy. Cancer Discovery.

[bib23] Ferraro GB, Ali A, Luengo A, Kodack DP, Deik A, Abbott KL, Bezwada D, Blanc L, Prideaux B, Jin X, Posada JM, Chen J, Chin CR, Amoozgar Z, Ferreira R, Chen IX, Naxerova K, Ng C, Westermark AM, Duquette M, Roberge S, Lindeman NI, Lyssiotis CA, Nielsen J, Housman DE, Duda DG, Brachtel E, Golub TR, Cantley LC, Asara JM, Davidson SM, Fukumura D, Dartois VA, Clish CB, Jain RK, Vander Heiden MG (2021). Fatty acid synthesis is required for breast cancer brain metastasis. Nature Cancer.

[bib24] Fisher-Wellman KH, Neufer PD (2012). Linking mitochondrial bioenergetics to insulin resistance via redox biology. Trends in Endocrinology & Metabolism.

[bib25] Gao X, Lee K, Reid MA, Sanderson SM, Qiu C, Li S, Liu J, Locasale JW (2018). Serine availability influences mitochondrial dynamics and function through lipid metabolism. Cell Reports.

[bib26] Garcia-Bermudez J, Baudrier L, La K, Zhu XG, Fidelin J, Sviderskiy VO, Papagiannakopoulos T, Molina H, Snuderl M, Lewis CA, Possemato RL, Birsoy K (2018). Aspartate is a limiting metabolite for cancer cell proliferation under hypoxia and in tumours. Nature Cell Biology.

[bib27] Gibson G, Blass JP (1976). A relation between (NAD+)/(NADH) potentials and glucose utilization in rat brain slices. The Journal of Biological Chemistry.

[bib28] Gravel S-P, Hulea L, Toban N, Birman E, Blouin M-J, Zakikhani M, Zhao Y, Topisirovic I, St-Pierre J, Pollak M (2014). Serine deprivation enhances antineoplastic activity of biguanides. Cancer Research.

[bib29] Gullino PM, Clark SH, Grantham FH (1964). The interstitial fluid of solid tumors. Cancer Research.

[bib30] Hanahan D, Weinberg RA (2011). Hallmarks of cancer: the next generation. Cell.

[bib31] Handy DE, Loscalzo J (2012). Redox regulation of mitochondrial function. Antioxidants & Redox Signaling.

[bib32] Holness MJ, Sugden MC (2003). Regulation of pyruvate dehydrogenase complex activity by reversible phosphorylation. Biochemical Society Transactions.

[bib33] Hosios AM, Hecht VC, Danai LV, Johnson MO, Rathmell JC, Steinhauser ML, Manalis SR, Vander Heiden MG (2016). Amino acids rather than glucose account for the majority of cell mass in proliferating mammalian cells. Developmental Cell.

[bib34] Hosios AM, Li Z, Lien EC, Heiden MVG (2018). Preparation of lipid-stripped serum for the study of lipid metabolism in cell culture. Bio-protocol.

[bib35] Hosios AM, Vander Heiden MG (2018). The redox requirements of proliferating mammalian cells. The Journal of Biological Chemistry.

[bib36] Krall AS, Mullen PJ, Surjono F, Momcilovic M, Schmid EW, Halbrook CJ, Thambundit A, Mittelman SD, Lyssiotis CA, Shackelford DB, Knott SRV, Christofk HR (2021). Asparagine couples mitochondrial respiration to ATF4 activity and tumor growth. Cell Metabolism.

[bib37] Labuschagne CF, van den Broek NJF, Mackay GM, Vousden KH, Maddocks ODK (2014). Serine, but not glycine, supports one-carbon metabolism and proliferation of cancer cells. Cell Reports.

[bib38] Lapuente-Brun E, Moreno-Loshuertos R, Acín-Pérez R, Latorre-Pellicer A, Colás C, Balsa E, Perales-Clemente E, Quirós PM, Calvo E, Rodríguez-Hernández MA, Navas P, Cruz R, Carracedo Á, López-Otín C, Pérez-Martos A, Fernández-Silva P, Fernández-Vizarra E, Enríquez JA (2013). Supercomplex assembly determines electron flux in the mitochondrial electron transport chain. Science.

[bib39] Li Z, Ji BW, Dixit PD, Tchourine K, Lien EC, Hosios AM, Abbott KL, Rutter JC, Westermark AM, Gorodetsky EF, Sullivan LB, Vander Heiden MG, Vitkup D (2022). Cancer cells depend on environmental lipids for proliferation when electron acceptors are limited. Nature Metabolism.

[bib40] Locasale JW, Grassian AR, Melman T, Lyssiotis CA, Mattaini KR, Bass AJ, Heffron G, Metallo CM, Muranen T, Sharfi H, Sasaki AT, Anastasiou D, Mullarky E, Vokes NI, Sasaki M, Beroukhim R, Stephanopoulos G, Ligon AH, Meyerson M, Richardson AL, Chin L, Wagner G, Asara JM, Brugge JS, Cantley LC, Vander Heiden MG (2011). Phosphoglycerate dehydrogenase diverts glycolytic flux and contributes to oncogenesis. Nature Genetics.

[bib41] Luengo A, Li Z, Gui DY, Sullivan LB, Zagorulya M, Do BT, Ferreira R, Naamati A, Ali A, Lewis CA, Thomas CJ, Spranger S, Matheson NJ, Vander Heiden MG (2021). Increased demand for NAD^+^ relative to ATP drives aerobic glycolysis. Molecular Cell.

[bib42] Lyssiotis CA, Kimmelman AC (2017). Metabolic interactions in the tumor microenvironment. Trends in Cell Biology.

[bib43] Maddocks ODK, Berkers CR, Mason SM, Zheng L, Blyth K, Gottlieb E, Vousden KH (2013). Serine starvation induces stress and p53-dependent metabolic remodelling in cancer cells. Nature.

[bib44] Maddocks ODK, Athineos D, Cheung EC, Lee P, Zhang T, van den Broek NJF, Mackay GM, Labuschagne CF, Gay D, Kruiswijk F, Blagih J, Vincent DF, Campbell KJ, Ceteci F, Sansom OJ, Blyth K, Vousden KH (2017). Modulating the therapeutic response of tumours to dietary serine and glycine starvation. Nature.

[bib45] McLaughlin SG, Dilger JP (1980). Transport of protons across membranes by weak acids. Physiological Reviews.

[bib46] Murphy JP, Giacomantonio MA, Paulo JA, Everley RA, Kennedy BE, Pathak GP, Clements DR, Kim Y, Dai C, Sharif T, Gygi SP, Gujar S (2018). The NAD^+^ salvage pathway supports PHGDH-driven serine biosynthesis. Cell Reports.

[bib47] Newman AC, Maddocks ODK (2017). One-carbon metabolism in cancer. British Journal of Cancer.

[bib48] Ngo B, Kim E, Osorio-Vasquez V, Doll S, Bustraan S, Liang RJ, Luengo A, Davidson SM, Ali A, Ferraro GB, Fischer GM, Eskandari R, Kang DS, Ni J, Plasger A, Rajasekhar VK, Kastenhuber ER, Bacha S, Sriram RK, Stein BD, Bakhoum SF, Snuderl M, Cotzia P, Healey JH, Mainolfi N, Suri V, Friedman A, Manfredi M, Sabatini DM, Jones DR, Yu M, Zhao JJ, Jain RK, Keshari KR, Davies MA, Vander Heiden MG, Hernando E, Mann M, Cantley LC, Pacold ME (2020). Limited environmental serine and glycine confer brain metastasis sensitivity to PHGDH inhibition. Cancer Discovery.

[bib49] Nobes CD, Lakin-Thomas PL, Brand MD (1989). The contribution of ATP turnover by the Na+/K+-ATPase to the rate of respiration of hepatocytes. Biochimica et Biophysica Acta - Bioenergetics.

[bib50] Novelli GD, Lipmann F (1950). The catalytic function of coenzyme a in citric acid synthesis. Journal of Biological Chemistry.

[bib51] Patel MS, Korotchkina LG (2006). Regulation of the pyruvate dehydrogenase complex. Biochemical Society Transactions.

[bib52] Pavlova NN, Thompson CB (2016). The emerging hallmarks of cancer metabolism. Cell Metabolism.

[bib53] Possemato R, Marks KM, Shaul YD, Pacold ME, Kim D, Birsoy K, Sethumadhavan S, Woo H, Jang HG, Jha AK, Chen WW, Barrett FG, Stransky N, Tsun Z, Cowley GS, Barretina J, Kalaany J, Kalaany NY, Hsu K, Ottina AM, Yuan B, Garrayway LA, Root DE, Mino-Kenudson M, Brachtel EF, Driggers EM, Sabatini DM (2011). Functional genomics reveals serine synthesis is essential in PHGDH-amplified breast cancer. Nature.

[bib54] Qiu F, Chen YR, Liu X, Chu CY, Shen LJ, Xu J, Gaur S, Forman HJ, Zhang H, Zheng S, Yen Y, Huang J, Kung HJ, Ann DK (2014). Arginine starvation impairs mitochondrial respiratory function in ASS1-deficient breast cancer cells. Science Signaling.

[bib55] Rabinovich S, Adler L, Yizhak K, Sarver A, Silberman A, Agron S, Stettner N, Sun Q, Brandis A, Helbling D, Korman S, Itzkovitz S, Dimmock D, Ulitsky I, Nagamani SCS, Ruppin E, Erez A (2015). Diversion of aspartate in ASS1-deficient tumours fosters de novo pyrimidine synthesis. Nature.

[bib56] Sciacovelli M, Dugourd A, Jimenez LV, Yang M, Nikitopoulou E, Costa ASH, Tronci L, Caraffini V, Rodrigues P, Schmidt C, Ryan DG, Young T, Zecchini VR, Rossi SH, Massie C, Lohoff C, Masid M, Hatzimanikatis V, Kuppe C, Von Kriegsheim A, Kramann R, Gnanapragasam V, Warren AY, Stewart GD, Erez A, Vanharanta S, Saez-Rodriguez J, Frezza C (2022). Dynamic partitioning of branched-chain amino acids-derived nitrogen supports renal cancer progression. Nature Communications.

[bib57] Sivanand S, Gultekin Y, Winter PS, Vermeulen SY, Tchourine KM, Abbott KL, Danai LV, Gourgue F, Do BT, Crowder K, Kunchok T, Lau AN, Darnell AM, Jefferson A, Morita S, Duda DG, Aguirre AJ, Wolpin BM, Henning N, Spanoudaki V, Maiorino L, Irvine DJ, Yilmaz OH, Lewis CA, Vitkup D, Shalek AK, Vander Heiden MG (2024). Cancer tissue of origin constrains the growth and metabolism of metastases. Nature Metabolism.

[bib58] Srere PA (1974). Controls of citrate synthase activity. Life Sciences.

[bib59] Sullivan LB, Gui DY, Hosios AM, Bush LN, Freinkman E, Vander Heiden MG (2015). Supporting aspartate biosynthesis is an essential function of respiration in proliferating cells. Cell.

[bib60] Sullivan MR, Danai LV, Lewis CA, Chan SH, Gui DY, Kunchok T, Dennstedt EA, Vander Heiden MG, Muir A (2019a). Quantification of microenvironmental metabolites in murine cancers reveals determinants of tumor nutrient availability. eLife.

[bib61] Sullivan MR, Mattaini KR, Dennstedt EA, Nguyen AA, Sivanand S, Reilly MF, Meeth K, Muir A, Darnell AM, Bosenberg MW, Lewis CA, Vander Heiden MG (2019b). Increased serine synthesis provides an advantage for tumors arising in tissues where serine levels are limiting. Cell Metabolism.

[bib62] Terada H (1990). Uncouplers of oxidative phosphorylation. Environmental Health Perspectives.

[bib63] Titov DV, Cracan V, Goodman RP, Peng J, Grabarek Z, Mootha VK (2016). Complementation of mitochondrial electron transport chain by manipulation of the NAD+/NADH ratio. Science.

[bib64] Vander Heiden MG, Chandel NS, Schumacker PT, Thompson CB (1999). Bcl-xL prevents cell death following growth factor withdrawal by facilitating mitochondrial ATP/ADP exchange. Molecular Cell.

[bib65] Vander Heiden MG, DeBerardinis RJ (2017). Understanding the Intersections between metabolism and cancer biology. Cell.

[bib66] Vichai V, Kirtikara K (2006). Sulforhodamine B colorimetric assay for cytotoxicity screening. Nature Protocols.

[bib67] Wang Y, Stancliffe E, Fowle-Grider R, Wang R, Wang C, Schwaiger-Haber M, Shriver LP, Patti GJ (2022). Saturation of the mitochondrial NADH shuttles drives aerobic glycolysis in proliferating cells. Molecular Cell.

[bib68] Wu Z, Bezwada D, Cai F, Harris RC, Ko B, Sondhi V, Pan C, Vu HS, Nguyen PT, Faubert B, Cai L, Chen H, Martin-Sandoval M, Do D, Gu W, Zhang Y, Zhang Y, Brooks B, Kelekar S, Zacharias LG, Oaxaca KC, Patricio JS, Mathews TP, Garcia-Bermudez J, Ni M, DeBerardinis RJ (2024). Electron transport chain inhibition increases cellular dependence on purine transport and salvage. Cell Metabolism.

[bib69] Xiao Y, Yu TJ, Xu Y, Ding R, Wang YP, Jiang YZ, Shao ZM (2023). Emerging therapies in cancer metabolism. Cell Metabolism.

[bib70] Yao CH, Wang R, Wang Y, Kung CP, Weber JD, Patti GJ (2019). Mitochondrial fusion supports increased oxidative phosphorylation during cell proliferation. eLife.

[bib71] Zhao R-Z, Jiang S, Zhang L, Yu Z-B (2019). Mitochondrial electron transport chain, ROS generation and uncoupling (Review). International Journal of Molecular Medicine.

