# Supplementary material for: Cancer cells differentially modulate mitochondrial respiration to alter redox state and enable biomass synthesis in nutrient-limited environments
Source: eLife. 2026 Nov 4;14:RP107123. doi: 10.7554/eLife.107123 (PMC13734678; doi:10.7554/eLife.107123)
Supplement: Supplementary file 3 [file elife-107123-fig1-figsupp1-data2.zip › Fig1-figsup1-sourcedata2.pdf]

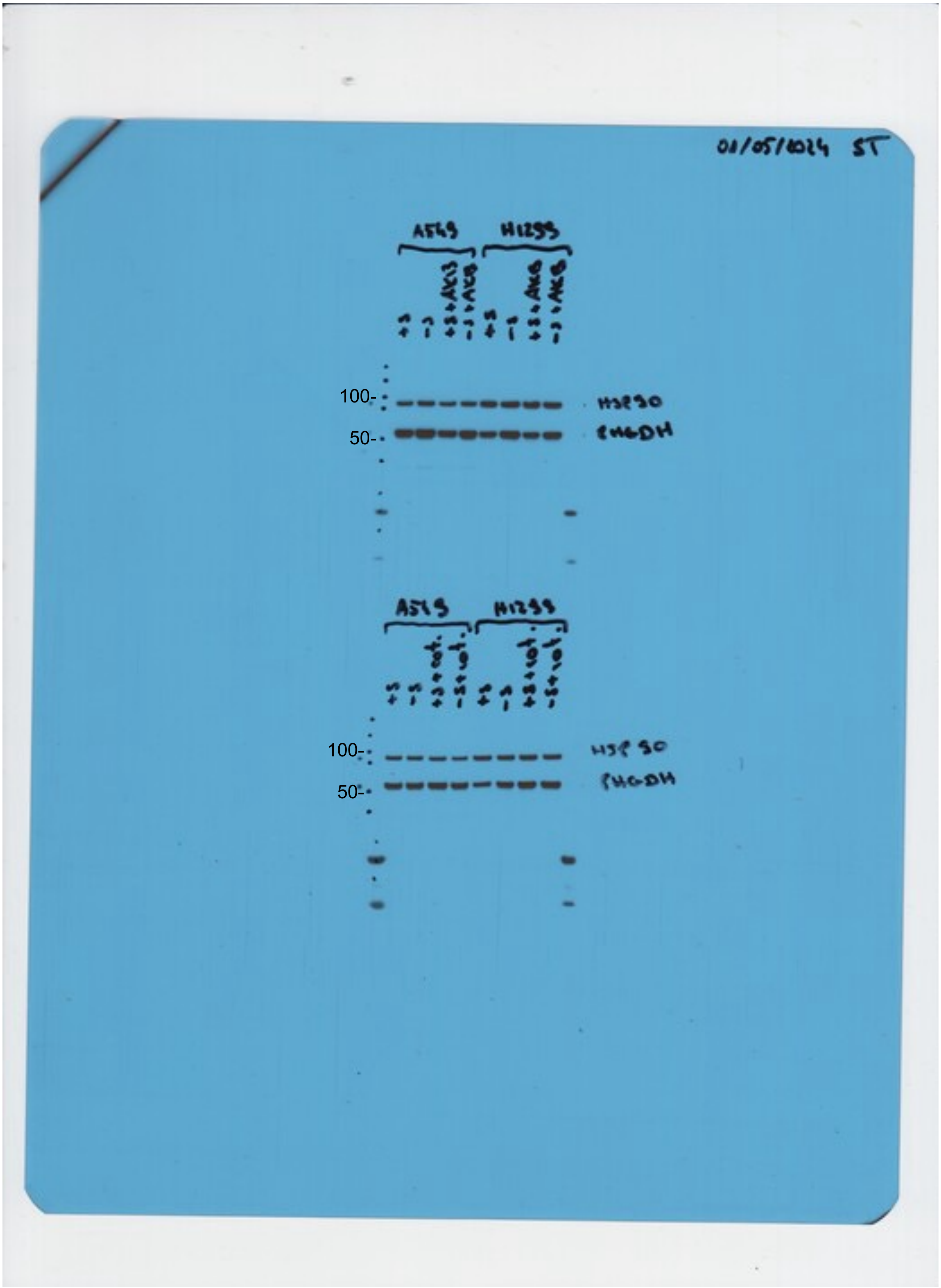


**Figure 1-figure supplement 1, Source Data 2.** Original films corresponding to Figure 1-figure supplement 1 panel A (top blot, first four lanes) and panel B (bottom blot, first four lanes). +s = serine replete; -s = serine deplete; + AKB = 1 mM AKB treated; + rot = 20 nM rotenone treated
