# Supplementary material for: Cancer cells differentially modulate mitochondrial respiration to alter redox state and enable biomass synthesis in nutrient-limited environments
Source: eLife. 2026 Nov 4;14:RP107123. doi: 10.7554/eLife.107123 (PMC13734678; doi:10.7554/eLife.107123)
Supplement: Supplementary file 10 [file elife-107123-fig2-figsupp1-data5.zip › Fig2-figsup1 sourcedata5.pdf]

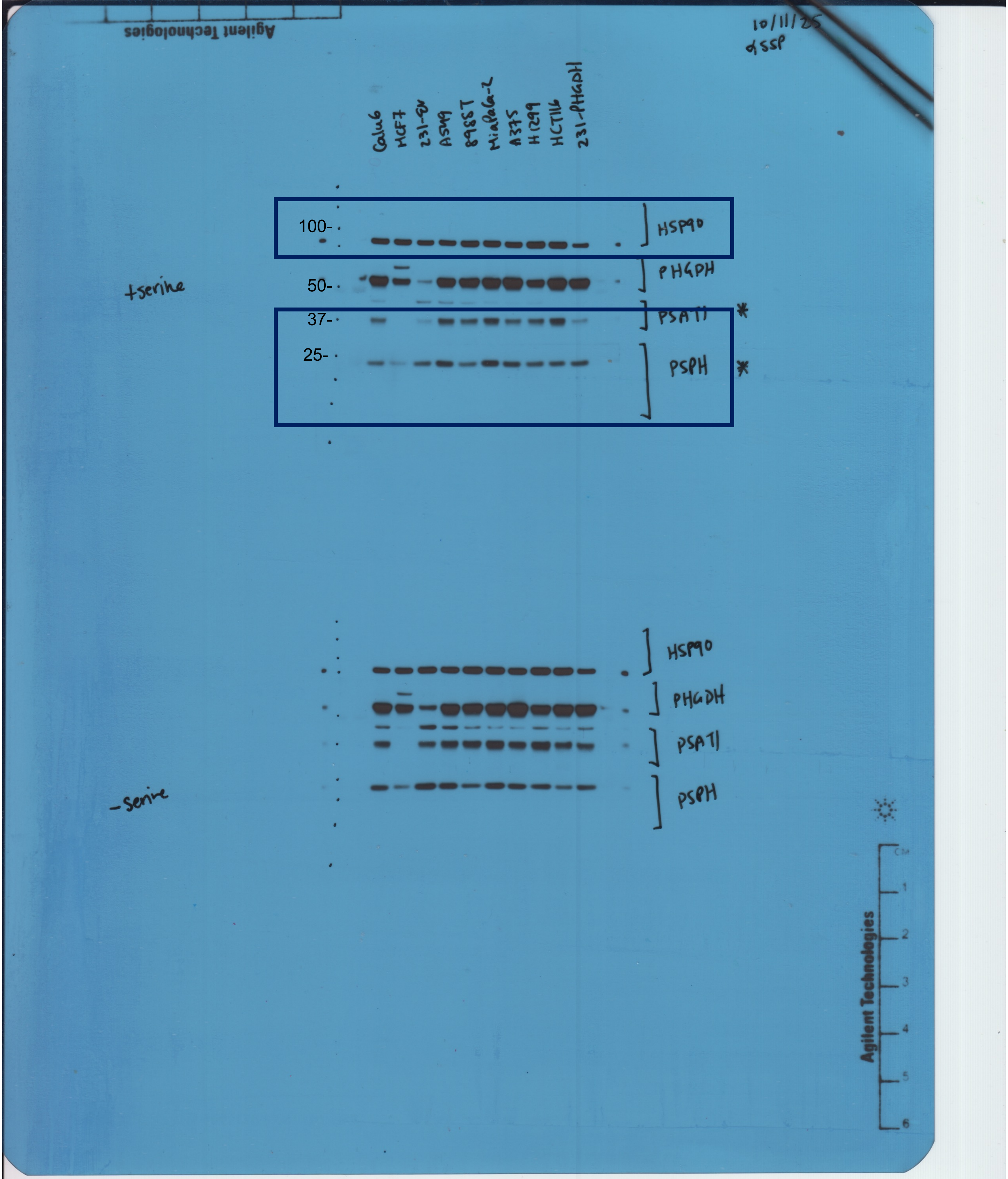

Figure 2-figure supplement 1, Source Data 5. Original film corresponding to Figure 2-figure supplement 1 panel B displaying HSP90, PSAT1, and PSPH protein expression in cells cultured with serine as indicated with dark blue box. Original film is the same film displayed in Figure 2-figure supplement 1 source data 4 and 6 after a different length of exposure.
