# Supplementary material for: Cancer cells differentially modulate mitochondrial respiration to alter redox state and enable biomass synthesis in nutrient-limited environments
Source: eLife. 2026 Nov 4;14:RP107123. doi: 10.7554/eLife.107123 (PMC13734678; doi:10.7554/eLife.107123)
Supplement: Supplementary file 11 [file elife-107123-fig2-figsupp1-data6.zip › Fig2-figsup1 sourcedata6.pdf]

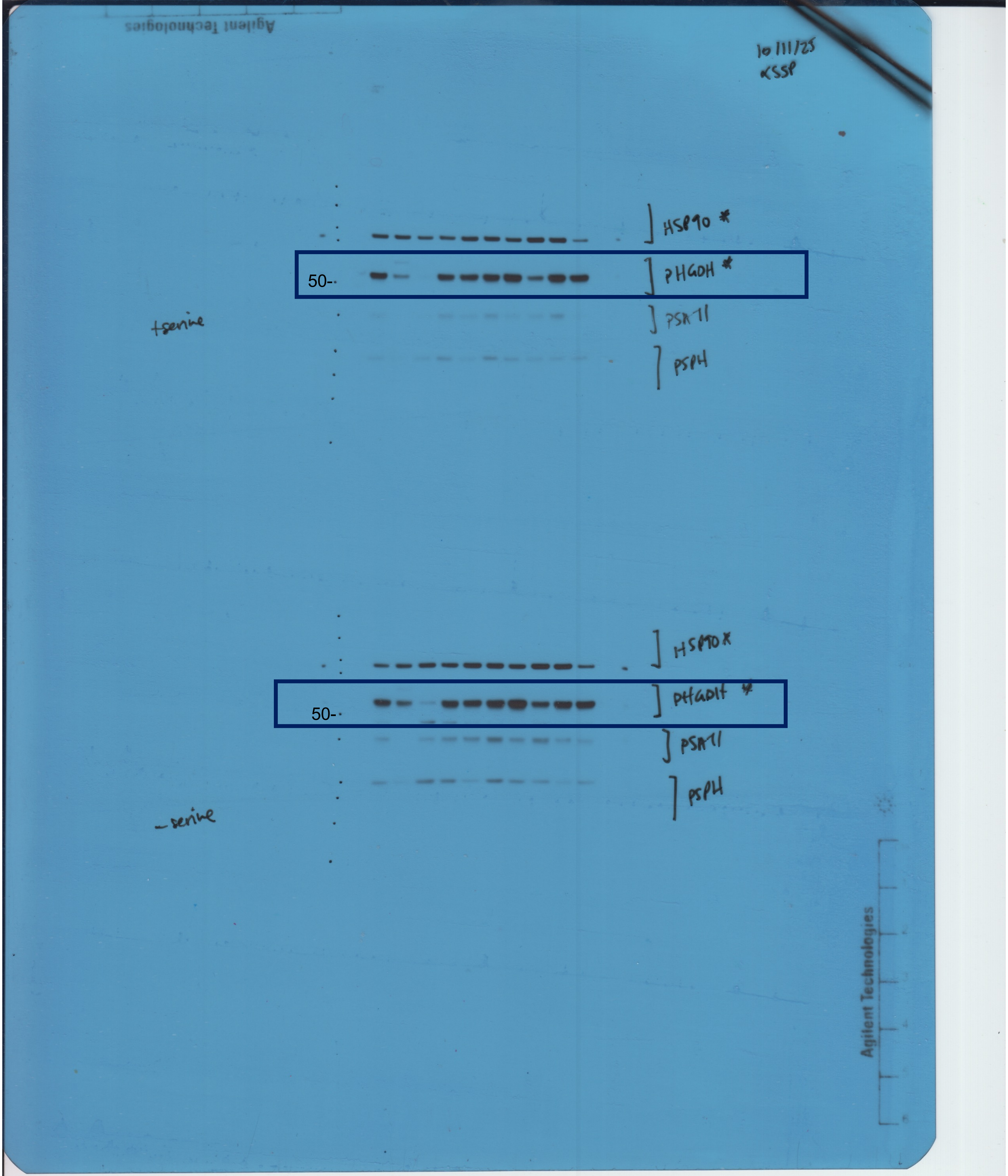

Figure 2-figure supplement 1, Source Data 6. Original film corresponding to Figure 2-figure supplement 1 panel B displaying PHGDH in cultured with serine (above) and without serine (below), as indicated by dark blue box. Original film is the same film displayed in Figure 2-figure supplement 1 source data 4 and 5 after a different length of exposure.
