# Supplementary material for: Cancer cells differentially modulate mitochondrial respiration to alter redox state and enable biomass synthesis in nutrient-limited environments
Source: eLife. 2026 Nov 4;14:RP107123. doi: 10.7554/eLife.107123 (PMC13734678; doi:10.7554/eLife.107123)
Supplement: Supplementary file 15 [file elife-107123-fig3-figsupp1-data2.zip › Fig3-figsup1 sourcedata2.pdf]

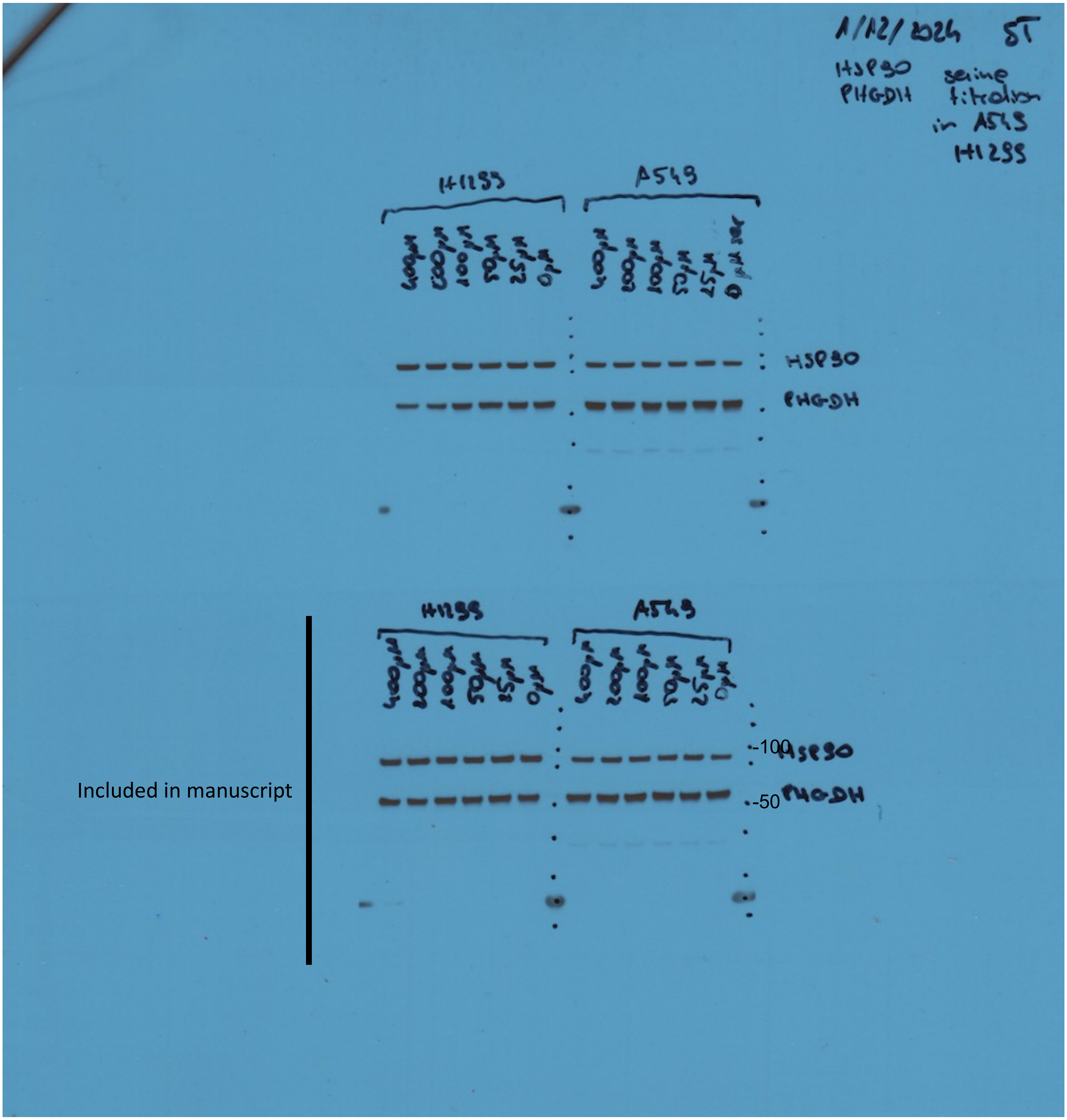

Figure 3-figure supplement 1, Source Data 2. Original film corresponding to Figure 3-figure supplement 1 panel G. The immunoblot used in the manuscript is a mirrored image of the original film to maintain consistent labeling with panel F.
