# Supplementary material for: Cancer cells differentially modulate mitochondrial respiration to alter redox state and enable biomass synthesis in nutrient-limited environments
Source: eLife. 2026 Nov 4;14:RP107123. doi: 10.7554/eLife.107123 (PMC13734678; doi:10.7554/eLife.107123)
Supplement: Supplementary file 19 [file elife-107123-fig4-figsupp1-data2.zip › Fig4-figsup1 sourcedata2.pdf]

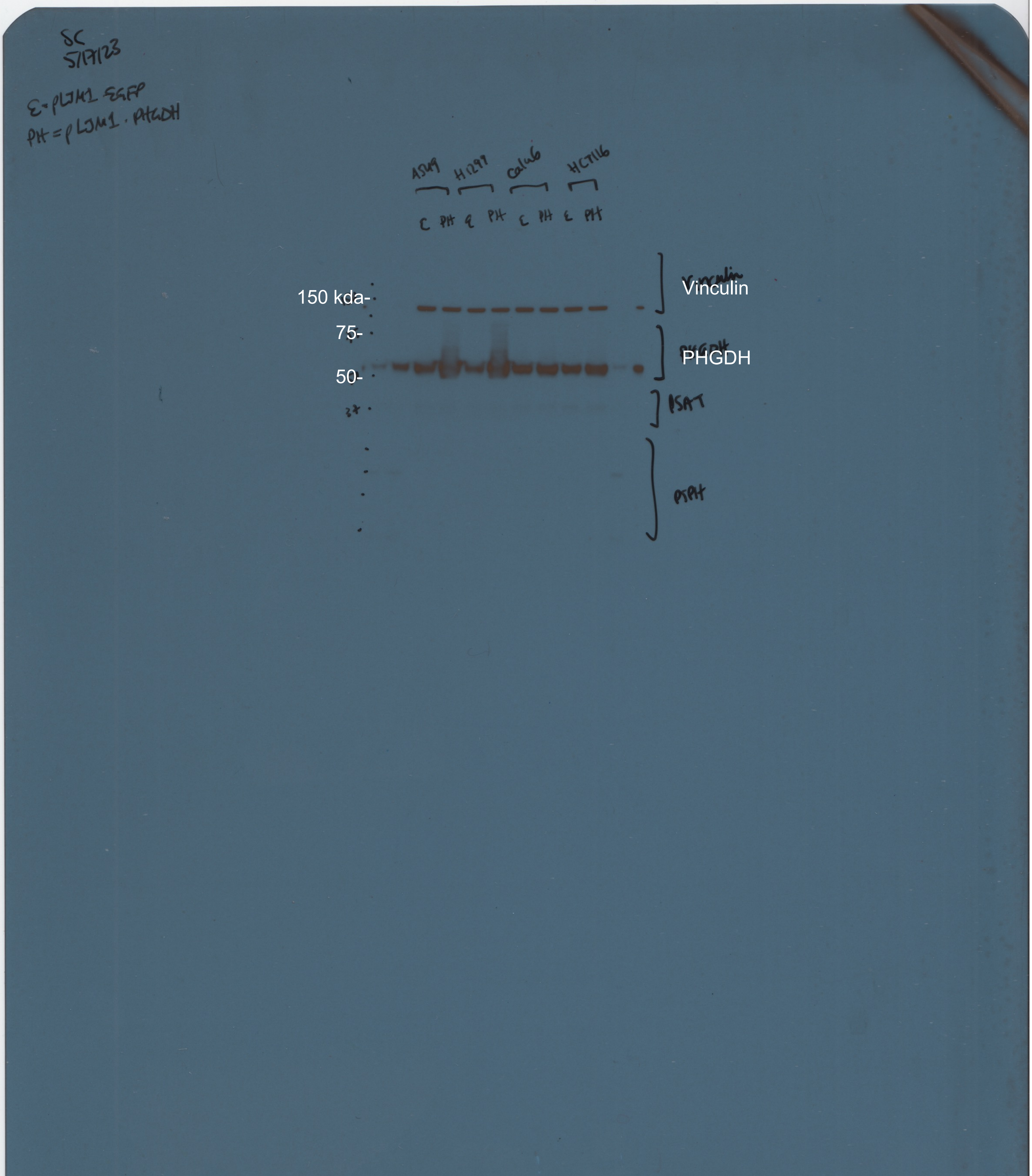

Figure 4-figure supplement 1g, Source Data 2. Original films corresponding to Figure 4-figure supplemental 1 Panel G. Each lane corresponds to the indicated cell line as labeled on the original film. First and last lanes were loaded with mixed samples to minimize risk of smiling and are not shown in the manuscript. Top bands correspond to Vinculin loading control. Bottom bands correspond to PHGDH protein.
