# Supplementary material for: Cancer cells differentially modulate mitochondrial respiration to alter redox state and enable biomass synthesis in nutrient-limited environments
Source: eLife. 2026 Nov 4;14:RP107123. doi: 10.7554/eLife.107123 (PMC13734678; doi:10.7554/eLife.107123)
Supplement: Supplementary file 21 [file elife-107123-fig4-figsupp1-data4.zip › Fig4-figsup1 sourcedata4.pdf]

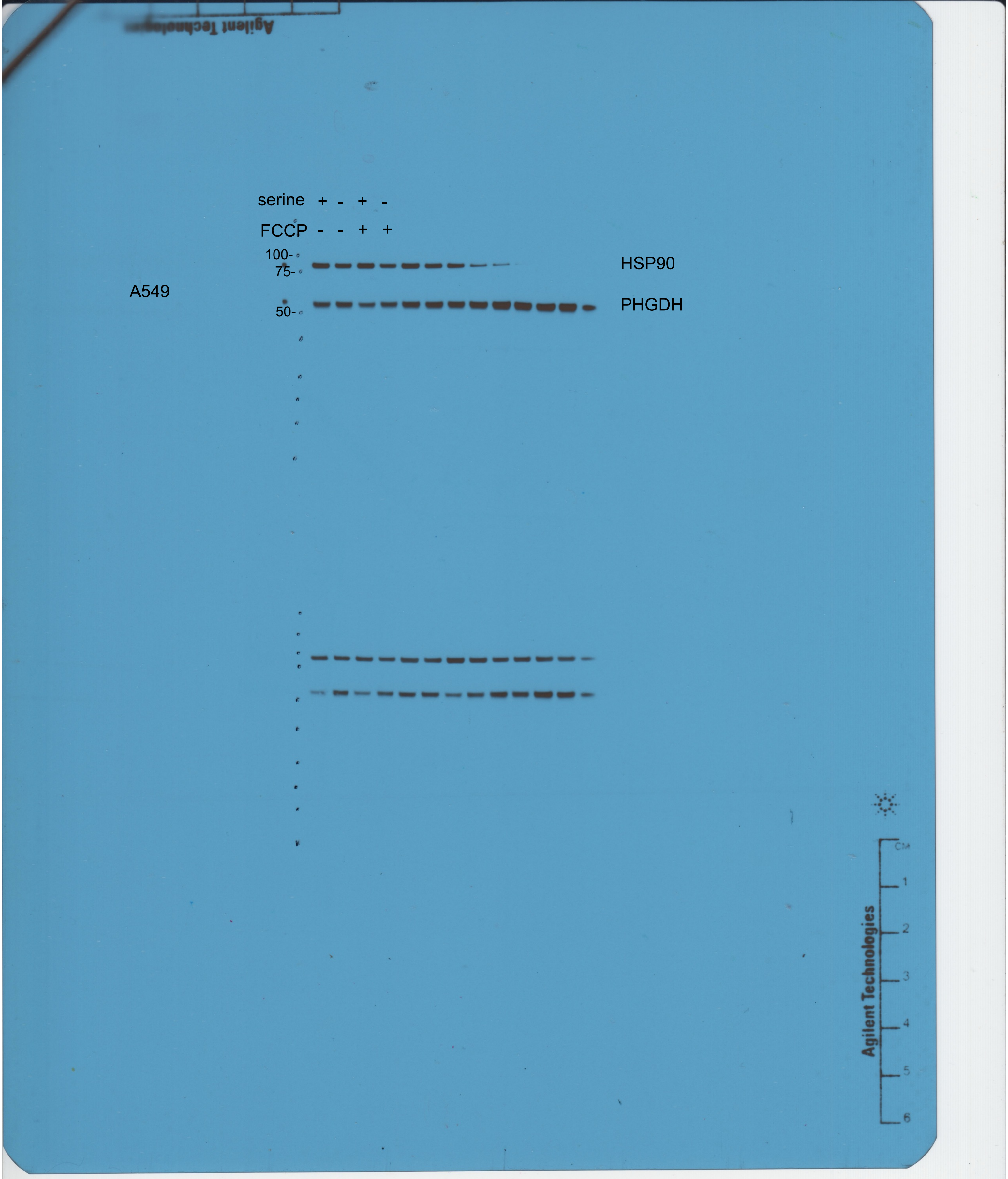

Figure 4-figure supplement 1, Source Data 4. Original films corresponding to Figure 4-figure supplement 1 panel J. The four lanes labeled are included in the manuscript and represent A549 cells treated with or without serine or FCCP for 24 hours as indicated.
