# Supplementary material for: Cancer cells differentially modulate mitochondrial respiration to alter redox state and enable biomass synthesis in nutrient-limited environments
Source: eLife. 2026 Nov 4;14:RP107123. doi: 10.7554/eLife.107123 (PMC13734678; doi:10.7554/eLife.107123)
Supplement: Supplementary file 26 [file elife-107123-fig4-figsupp3-data3.zip › Fig4-figsup3 sourcedata3.pdf]

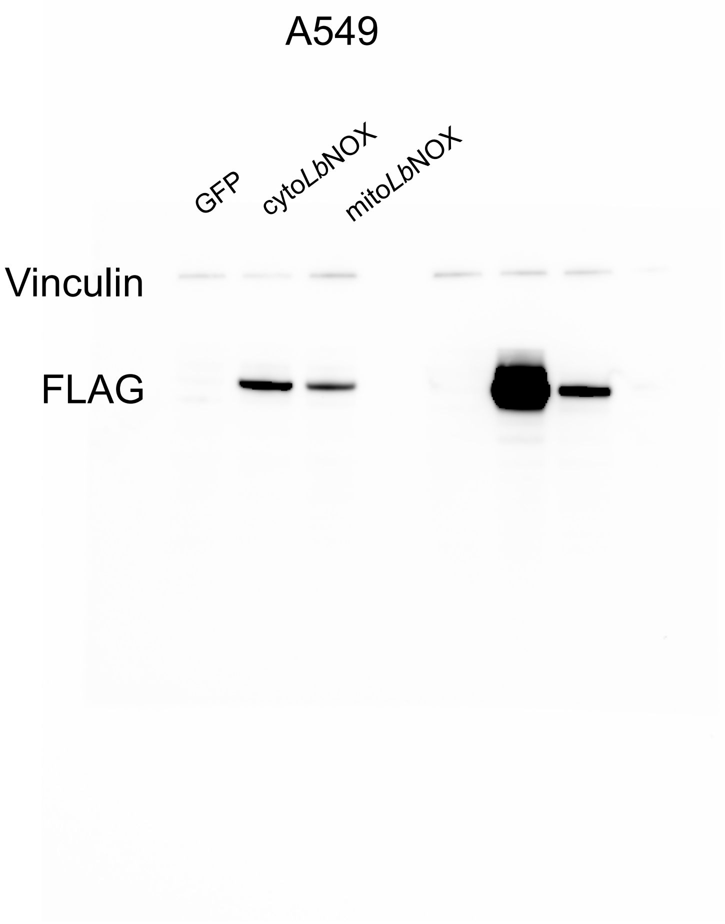

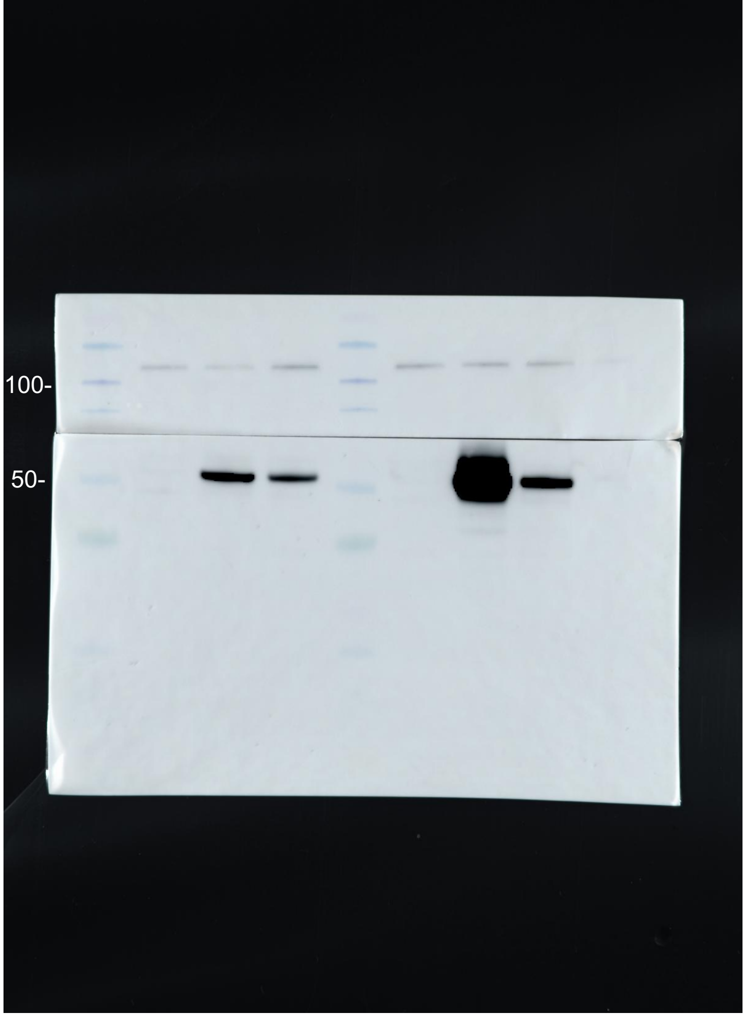

Figure 4-figure supplement 3, Source Data 3. Original membrane corresponding to Figure 4-figure supplement 3 panel A FLAG without Precision Plus Protein Kaleidoscope ladder (left) and with Precision Plus Protein Kaleidoscope Ladder (right). Manuscript includes the first three lanes that are labeled.
