# Supplementary material for: Cancer cells differentially modulate mitochondrial respiration to alter redox state and enable biomass synthesis in nutrient-limited environments
Source: eLife. 2026 Nov 4;14:RP107123. doi: 10.7554/eLife.107123 (PMC13734678; doi:10.7554/eLife.107123)
Supplement: Supplementary file 32 [file elife-107123-fig4-figsupp4-data4.zip › Fig4-figsup4 sourcedata4.pdf]

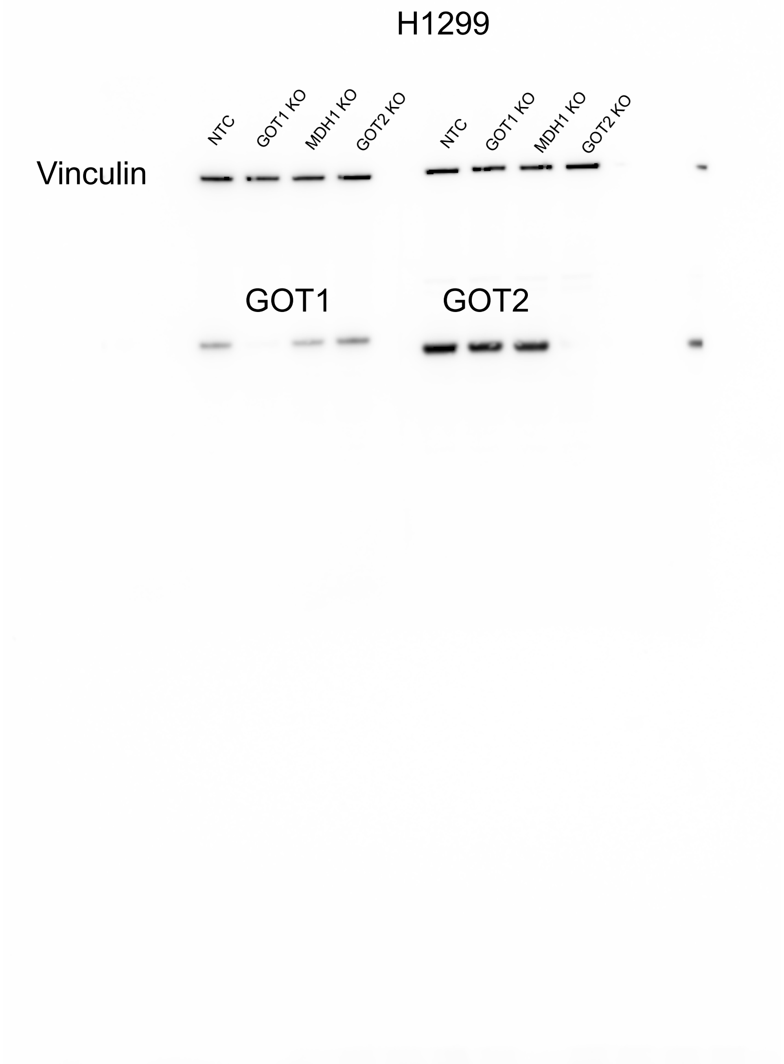

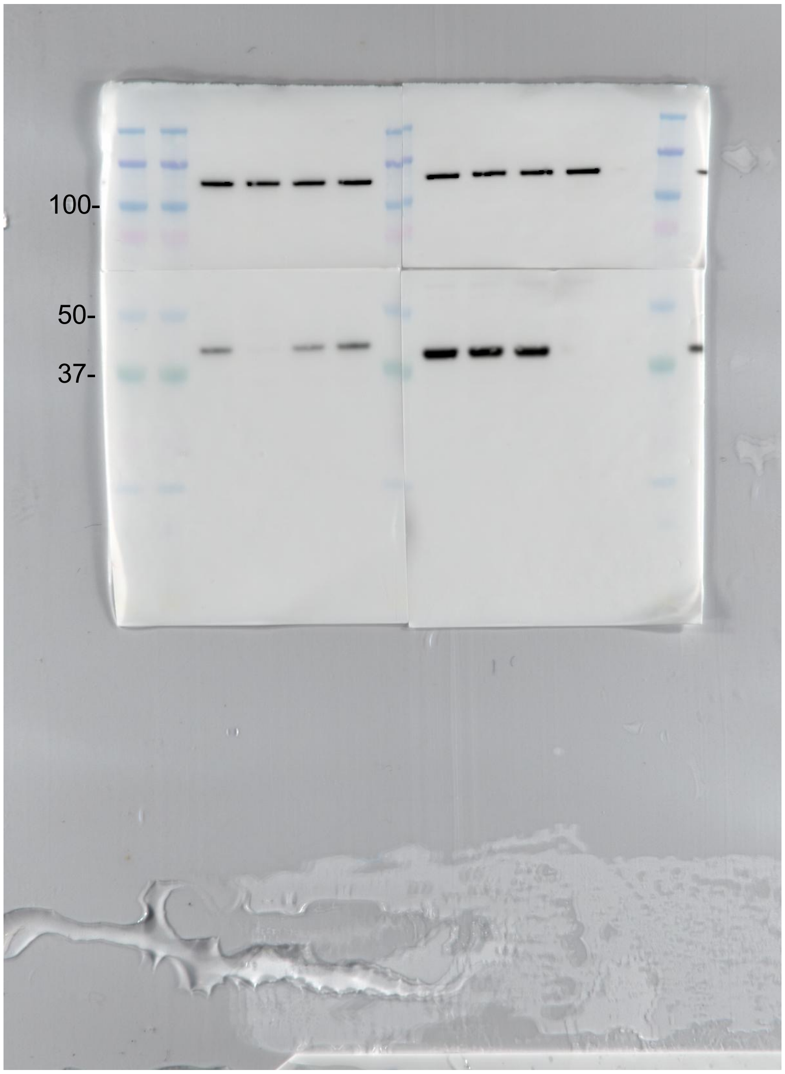

Figure 4-figure supplement 4, Source Data 4. Original membrane corresponding to Figure 4-figure supplement 4 panel A displaying protein expression of GOT1, GOT2, and the associated Vinculin loading control without Precision Plus Protein Kaleidoscope ladder (left) and with Precision Plus Protein Kaleidoscope Ladder (right).
