# Supplementary material for: Cancer cells differentially modulate mitochondrial respiration to alter redox state and enable biomass synthesis in nutrient-limited environments
Source: eLife. 2026 Nov 4;14:RP107123. doi: 10.7554/eLife.107123 (PMC13734678; doi:10.7554/eLife.107123)
Supplement: Supplementary file 33 [file elife-107123-fig4-figsupp4-data5.zip › Fig4-figsup4 sourcedata5.pdf]

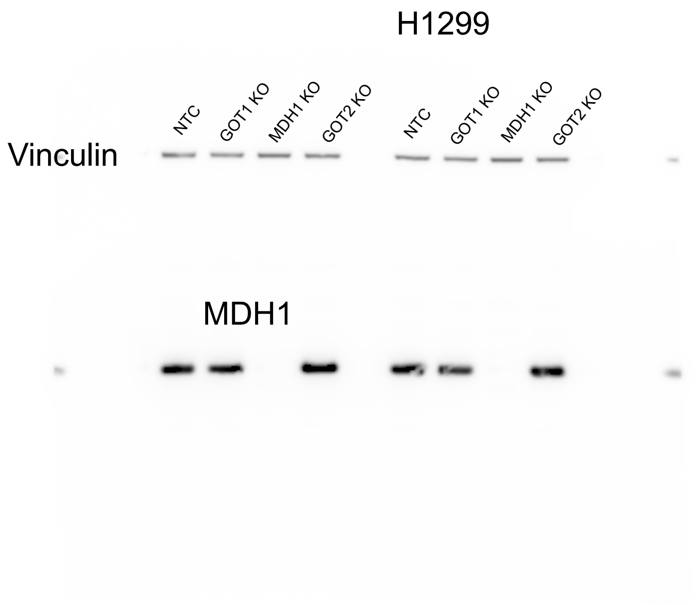

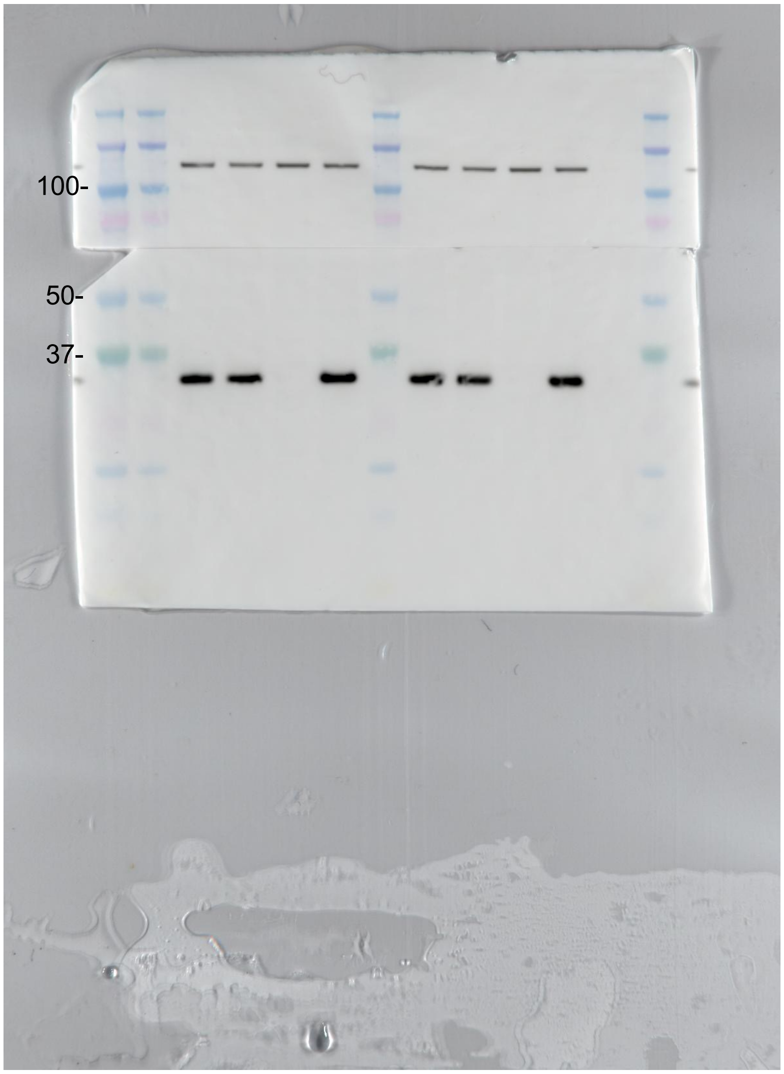

Figure 4-figure supplement 4, Source Data 5. Original membrane corresponding to Figure 4-figure supplement 4 panel A displaying MDH1 protein expression without Precision Plus Protein Kaleidoscope ladder (left) and with Precision Plus Protein Kaleidoscope Ladder (right). Bands displaying MDH1 are included in the manuscript. See Figure 4-figure supplement 4 Source Data 6 for the associated bands corresponding to the Vinculin loading control.
