# Supplementary material for: Cancer cells differentially modulate mitochondrial respiration to alter redox state and enable biomass synthesis in nutrient-limited environments
Source: eLife. 2026 Nov 4;14:RP107123. doi: 10.7554/eLife.107123 (PMC13734678; doi:10.7554/eLife.107123)
Supplement: Supplementary file 34 [file elife-107123-fig4-figsupp4-data6.zip › Fig4-figsup4 sourcedata6.pdf]

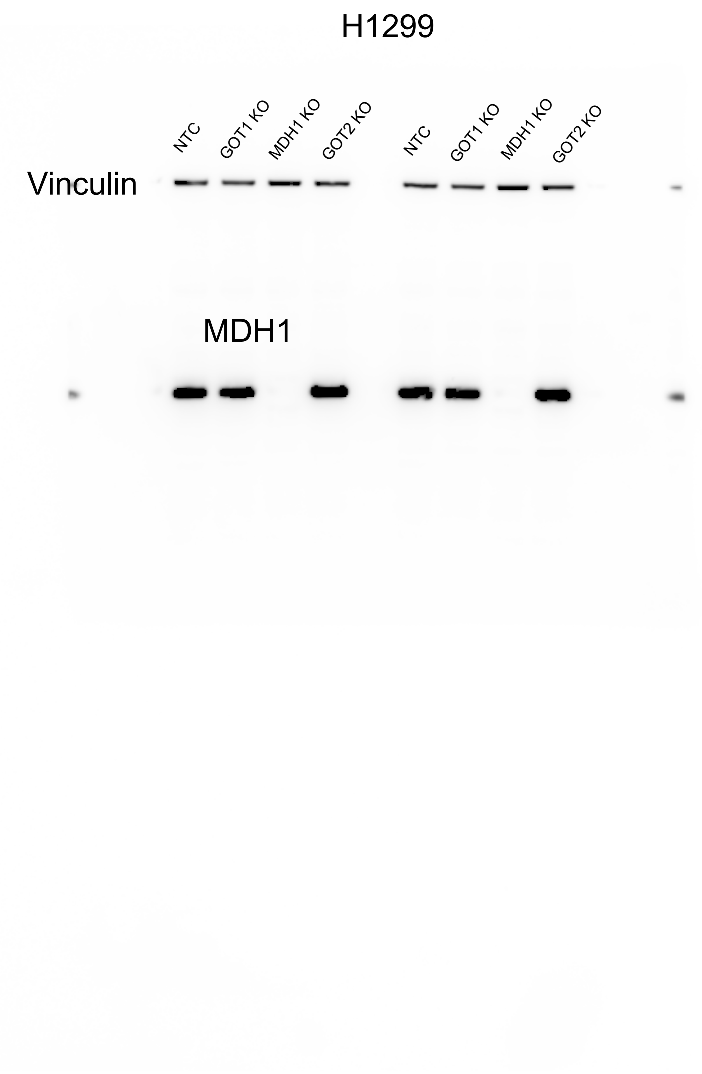

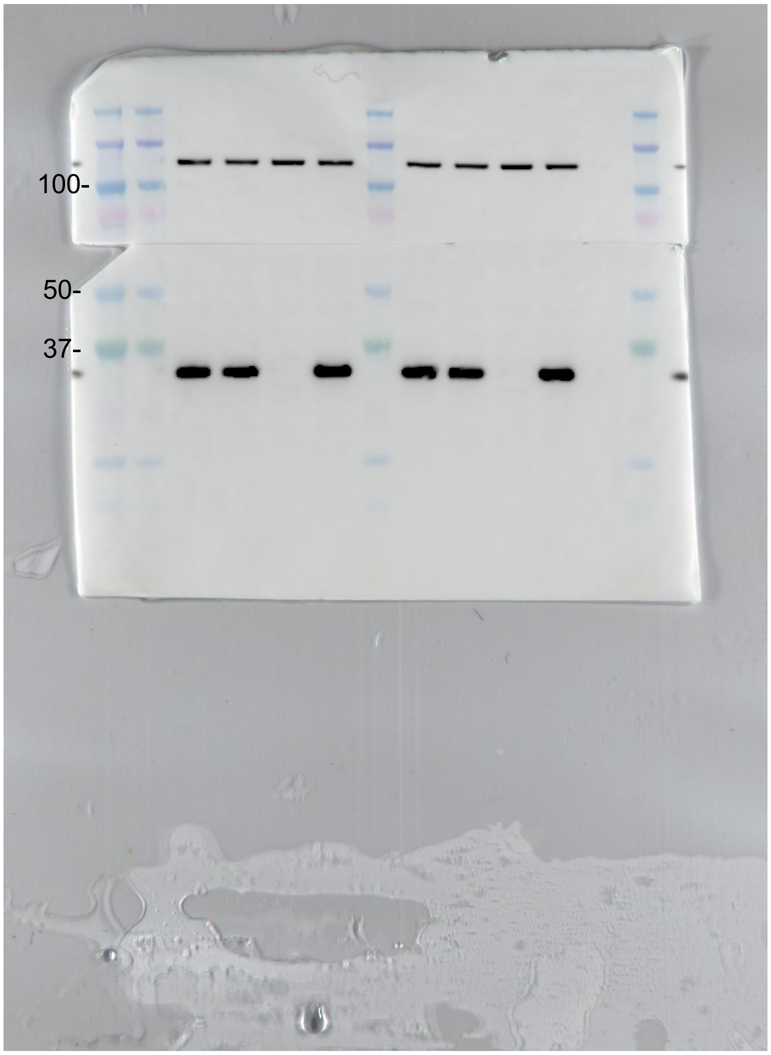

Figure 4-figure supplement 4, Source Data 6. Original membrane corresponding to Figure 4-figure supplement 4 panel A displaying Vinculin protein expression corresponding to immunoblot displaying MDH1 protein expression without Precision Plus Protein Kaleidoscope ladder (left) and with Precision Plus Protein Kaleidoscope Ladder (right). Bands displaying Vinculin are included in the manuscript. See Figure 4-figure supplement 4 Source Data 5 for the associated bands corresponding to MDH1 protein expression.
