# Supplementary material for: Cancer cells differentially modulate mitochondrial respiration to alter redox state and enable biomass synthesis in nutrient-limited environments
Source: eLife. 2026 Nov 4;14:RP107123. doi: 10.7554/eLife.107123 (PMC13734678; doi:10.7554/eLife.107123)
Supplement: Supplementary file 43 [file elife-107123-supp1.docx]

**Supplementary File 1**

| **Cell Line** | **Genetic Mutations** | **Cancer Type** | **Source** |
| --- | --- | --- | --- |
| Calu6 | Kras Q61K  TP53 R196* | Lung Adenocarcinoma | pleural effusion |
| A549 | Kras G12S  Stk11 Q37* | Lung Adenocarcinoma | lung |
| PaTu 8988T | Kras G12V  TP53 R282W  Smad4 del  EP300 del | Pancreatic ductal adenocarcinoma | liver metastasis |
| MIA PaCa-2 | Kras G12C  TP53 R248W  CDKN2A del | Pancreatic ductal adenocarcinoma | pancreas |
| H1299 | Nras Q61K  TP53 del | Lung Adenocarcinoma | lymph node metastasis |
| HCT116 | Kras G13D  BRCA2 I2675Dfs  CDKN2A mut  EP300 mut  PIK3CA H1047R  PPMID L450*  TGFBR2 K128Sfs | Colon Carcinoma | colon |
| A375 | BRAF V600E  CDKNA E61*  TERTc.1-146C>T | Melanoma | skin |
| MDA-MB-231 | BRAF G464V  Kras G13D  TP53 R280K  CDKN2A/B del  TERTc.1-146C>T | Triple Negative Breast Adenocarcinoma | pleural effusion |
| MCF7 | PIK3CA E545K  GATA3 D336Gfs  CDKN2A del | ER+ HER2- Breast Carcinoma | pleural effusion |
