# Supplementary material for: Cancer cells differentially modulate mitochondrial respiration to alter redox state and enable biomass synthesis in nutrient-limited environments
Source: eLife. 2026 Nov 4;14:RP107123. doi: 10.7554/eLife.107123 (PMC13734678; doi:10.7554/eLife.107123)
Supplement: Peer review history

# Editor report: eLife Assessment

**DOI:** 10.7554/eLife.107123.3.sa0

**Authors:** Mari L Gantner

**Affiliations:** The Lowy Medical Research Institute United States

**Keywords:** Convincing, Important

This study presents **important** findings on the relationship between nutrient availability and NAD/NADH levels, which in turn regulate biomass production in cancer cells. The authors provide **convincing** evidence to support their claims, offering insight into why it is difficult to predict which nutrients limit cancer cell growth: both cell type and nutrient availability together determine the oxidative capacity that constrains the synthesis of various metabolic intermediates. The manuscript will be of broad interest to researchers working in cancer and cell metabolism.

# Referee report: Reviewer #1 (Public review)

**DOI:** 10.7554/eLife.107123.3.sa1

Summary:

This manuscript investigates how cellular NAD/NADH ratios are controlled in cancer cell lines in vitro. The authors build on previous work, which shows that serine synthesis is sensitive to NAD/NADH ratios and PHGDH expression. Here, the authors demonstrate that serine synthesis is variable across a panel of cell lines, even when controlling for expression of serine synthesis enzymes such as PHGDH. The authors show that cellular NAD/NADH ratios correlate with the ability to synthesize serine and grow in serine-deprived environments when PHGDH levels remain constant. Investigating this variability in NAD/NADH ratios, the authors find that the cells that can positively respond to serine deprivation are able to increase oxygen consumption and cellular NAD/NADH ratios. Cells that do not increase oxygen consumption in response to serine deprivation do not increase NAD/NADH ratios and cannot grow well without serine. The authors go on to show that in cells with the ability to increase oxygen consumption upon serine deprivation, PHGDH expression alone is sufficient to fully restore growth-serine; in cells that cannot increase oxygen consumption, both PHGDH expression and interventions to increase NAD/NADH ratios are required to increase growth. Thus, cells need both PHGDH and NAD/NADH increases to maximize serine synthesis in response to serine deprivation. The authors previously showed that lipid synthesis likewise requires NAD regeneration. Interestingly, one cell line that does not increase oxygen consumption in response to serine limitation tends to increase oxygen consumption in response to lipid deprivation; accordingly, depriving this cell line of lipids increases the synthesis of serine. Together, these findings show that how cells respond to nutrient deprivation is highly variable and that the response to nutrient deprivation (for example, whether or not oxygen consumption is increased) will determine how well cells tolerate depletion of nutrients with related biosynthetic constraints. This work sheds light on the complexity of cancer cell metabolism and helps to explain why it is difficult to predict which nutrients will be limiting to any cancer cell type or environment.

Strengths:

(1) The authors use multiple interventions to manipulate NAD/NADH ratios in cells.

(2) Experiments are well controlled and appropriately interpreted.

Comments on revised version:

The authors thoughtfully and thoroughly responded to all reviewer comments. The revised manuscript addresses the critiques.

# Referee report: Reviewer #2 (Public review)

**DOI:** 10.7554/eLife.107123.3.sa2

In the manuscript "Cancer cells differentially modulate mitochondrial respiration to alter redox state and enable biomass synthesis in nutrient-limited environments", Chang et al investigate how cancer cells respond to the limitation of certain environmental nutrients by regulating the cellular NAD+/NADH ratio. They focus on serine and lipid metabolism, pathways known to be controlled by the NAD+/NADH ratio, and propose that changes in mitochondrial respiration in response to deprivation of these nutrients can influence the NAD+/NADH ratio, thereby impacting biomass synthesis.

While the study is descriptive in nature and does not investigate specific molecular mechanisms that explain the crosstalk between nutrient availability and mitochondrial redox changes, the experimental component is robust, and the conclusions are well supported by the results. Some suggestions could further refine the conclusions and enhance the quality of the manuscript.

Comments on revised version:

The authors have provided a very comprehensive response. Their updated paper has improved, and the critiques have been mitigated.

# Author comment: Author response

**DOI:** 10.7554/eLife.107123.3.sa3

**Authors:** Sarah M Chang, Muhammad Bin Munim, Sonia E Trojan, Huel Cox, Anna Shevzov-Zebrun, Keene L Abbott, Renee Chang, Matthew G Vander Heiden

**Affiliations:** Massachusetts Institute of Technology Cambridge United States; Massachusetts Institute of Technology Cambridge United States; Massachusetts Institute of Technology Cambridge United States; Massachusetts Institute of Technology Cambridge United States; Massachusetts Institute of Technology Cambridge United States; Stanford University Stanford United States; Massachusetts Institute of Technology Cambridge United States; Massachusetts Institute of Technology Cambridge United States

The following is the authors’ response to the original reviews.

**eLife Assessment**

This study presents valuable findings on the relationship between nutrient availability and NAD/NADH levels, which in turn regulate biomass production in cancer cells. The authors provide solid evidence to support their claims, offering insight into why it is difficult to predict which nutrients limit cancer cell growth: both cell type and nutrient availability together determine the oxidative capacity that constrains the synthesis of various metabolic intermediates. The manuscript will be of interest to researchers working in cancer and cell metabolism.

We thank the eLife Editor for evaluating our manuscript and for the positive comments.

**Reviewer #1 (Public review):**

Summary:

This manuscript investigates how cellular NAD/NADH ratios are controlled in cancer cell lines in vitro. The authors build on previous work, which shows that serine synthesis is sensitive to NAD/NADH ratios and PHGDH expression. Here, the authors demonstrate that serine synthesis is variable across a panel of cell lines, even when controlling for expression of serine synthesis enzymes such as PHGDH. The authors show that cellular NAD/NADH ratios correlate with the ability to synthesize serine and grow in serine-deprived environments when PHGDH levels remain constant. Investigating this variability in NAD/NADH ratios, the authors find that the cells that can positively respond to serine deprivation are able to increase oxygen consumption and cellular NAD/NADH ratios. Cells that do not increase oxygen consumption in response to serine deprivation do not increase NAD/NADH ratios and cannot grow well without serine. The authors go on to show that in cells with the ability to increase oxygen consumption upon serine deprivation, PHGDH expression alone is sufficient to fully restore growth-serine; in cells that cannot increase oxygen consumption, both PHGDH expression and interventions to increase NAD/NADH ratios are required to increase growth. Thus, cells need both PHGDH and NAD/NADH increases to maximize serine synthesis in response to serine deprivation. The authors previously showed that lipid synthesis likewise requires NAD regeneration. Interestingly, one cell line that does not increase oxygen consumption in response to serine limitation tends to increase oxygen consumption in response to lipid deprivation; accordingly, depriving this cell line of lipids increases the synthesis of serine. Together, these findings show that how cells respond to nutrient deprivation is highly variable and that the response to nutrient deprivation (for example, whether or not oxygen consumption is increased) will determine how well cells tolerate depletion of nutrients with related biosynthetic constraints. This work sheds light on the complexity of cancer cell metabolism and helps to explain why it is difficult to predict which nutrients will be limiting to any cancer cell type or environment.

Strengths:

(1) The authors use multiple interventions to manipulate NAD/NADH ratios in cells.

(2) Experiments are well controlled and appropriately interpreted.

Weaknesses:

Overall the data support the conclusions of the manuscript. I have only two minor comments and suggestions:

We thank Reviewer 1 for their insightful comments, which have helped us improve the manuscript.

(1) Figure 2B/C: data are presented as relative to +serine, which shows how some cells respond to -serine, but may also be of interest to see how absolute (not relative) NAD/NADH levels correlate with serine synthesis and serine-independent proliferation. In other words, is it the dynamic increase in the ratio that is most important, or the absolute level of the ratio?

We thank Reviewer 1 for raising this point about whether it is the absolute NAD+/NADH ratio, or the change in NAD+/NADH ratio, that is important for increasing serine synthesis and allowing proliferation under serine depleted conditions. We reported relative ratios for accessibility to a general audience, but agree that this information is informative and should be presented. We assessed the NAD+/NADH ratio using an enzymatic assay, which does not directly measure absolute concentrations of NAD+ or NADH (PMID: 26232225). However, we previously confirmed the assay is in the same linear range for both NAD+ and NADH, and thus is valid for assessing the NAD+/NADH ratio. We now provide the unnormalized NAD+/NADH ratio data in Supplementary Figure 2G of the revised manuscript. This shows that the considered cells exhibit a range of NAD+/NADH ratios, and redox responsive cells do not cluster in having a higher or lower NAD+/NADH ratio.

To more formally answer Reviewer 1’s question about whether the absolute ratio or change in ratio is important for increasing serine synthesis, we measured the correlation coefficient between the unnormalized NAD+/NADH ratios and the proliferation rate of all examined cancer cells cultured with or without serine. These data are presented in Author response image 1. Of note, we find that there is a significant positive correlation between the raw values of the measured NAD+/NADH ratio and proliferation rate in both serine-replete (r = .371) and serine depleted (r = .562) conditions. However, this correlation is not strong, and when examining the cancer cells whose proliferation in serine depleted conditions cannot be fully explained by serine synthesis enzyme expression (Calu6, 8988T, A549, MIA PaCa-2, H1299, and HCT116), there is no significant correlation between the raw NAD+/NADH ratio and proliferation rate in serine depleted conditions. The association between the relative change in the NAD+/NADH ratio and proliferation rate is much stronger upon serine deprivation (r = .571), as presented in Figure 2C of the revised manuscript. This suggests that the dynamic increase in the ratio is more tightly linked to the change in serine synthesis rate and proliferation in serine depleted environments, and we discuss this point in the revised manuscript with the following text:

“Of note, whether the NAD+/NADH ratio of a cell was more or less oxidized in serine-replete conditions was not predictive of response to serine withdrawal (Supplementary Figure 2G).” (Lines 163-165)

**Author response image 1.** Correlations between unnormalized NAD+/NADH ratios and cell proliferation rates between (A) all cancer cells examined (Calu6, MCF7, MDA-MB-231, A549, 8988T, MIA PaCa-2, A375, H1299, HCT116, MDA-MB-231 with PHGDH overexpression) in serine-replete conditions, (B) all cancer cells examined in serine depleted conditions, and (C) select cancer cells (labeled in gray) where serine synthesis enzyme protein expression does not fully explain proliferation in serine depleted conditions. Pearson correlation coefficient and P values were calculated by simple linear regression, *p<0.05, **p<0.01. Data shown are means of three biological replicates ± SD.

(2) Line 177-178: the authors write, "We hypothesized that the elevated NAD+/NADH ratio represented a cellular response to make the NAD+/NADH ratio more oxidized to enable serine synthesis". I recommend modest edits to avoid anthropomorphizing. It is possible that the ratio responds for reasons yet to be determined and not necessarily because the cell is deliberately trying to enable serine synthesis.

We thank Reviewer 1 for raising this point. We agree that our data do not show whether the ratio is elevated for the deliberate purpose of enabling serine synthesis and have edited the text accordingly with the following edit to that line of the revised manuscript:

“We hypothesized that a more oxidized NAD+/NADH ratio could support greater serine synthesis and thus sought to identify the processes that increase the NAD+/NADH ratio in some but not all cancer cells.” (Lines 190-192)

**Reviewer #2 (Public review):**

In the manuscript "Cancer cells differentially modulate mitochondrial respiration to alter redox state and enable biomass synthesis in nutrient-limited environments", Chang et al investigate how cancer cells respond to the limitation of certain environmental nutrients by regulating the cellular NAD+/NADH ratio. They focus on serine and lipid metabolism, pathways known to be controlled by the NAD+/NADH ratio, and propose that changes in mitochondrial respiration in response to deprivation of these nutrients can influence the NAD+/NADH ratio, thereby impacting biomass synthesis.

While the study is descriptive in nature and does not investigate specific molecular mechanisms that explain the crosstalk between nutrient availability and mitochondrial redox changes, the experimental component is robust, and the conclusions are well supported by the results. Some suggestions could further refine the conclusions and enhance the quality of the manuscript.

We thank Reviewer 2 for their time and for their suggestions to improve the manuscript.

Main critiques:

(1) Throughout the manuscript, the authors utilise the number of cell doublings per day as an endpoint readout of cell proliferation. It would be advisable to include a quantification of the cell number and to display the proliferation rate over time. This would provide valuable insights into the timeline of cellular responses and avoid potential confounding effects associated with the use of Sulforhodamine B dye, an indirect measure of cell proliferation based on protein content, which may be influenced by some of the interventions. Furthermore, it will help determine whether specific treatments reduce cellular doublings resulting from cell death. This concern is particularly evident in treatments with rotenone, e.g., Fig. 1G, where the increase in doublings could be attributed to cell death.

We thank the reviewer for this suggestion and agree that assessment of cell count provides additional information beyond Sulforhodamine B dye as an indirect measure of proliferation. To address this, we directly measured cell number over time using Incucyte Live-Cell imaging analysis applied to A549 and H1299 cells cultured with or without serine for 72 hours. Consistent with results using sulforhodamine B, A549 cells doubled at a rate of 0.874 per day and H1299 cells doubled at a rate of 1.034 per day in serine-replete conditions. In serine depleted conditions, A549 cells doubled at a rate of 0.205 per day while H1299 cells doubled at a rate of 0.544 per day. We have added the cell number measurements over time as well as the corresponding calculated doublings per day in Supplementary Figure 2D and Supplementary Figure 2E of the revised manuscript.

We also agree with Reviewer 2 that serine deprivation and rotenone treatment could potentially impact cell viability, which might confound phenotypes, including NAD+/NADH ratio measurements. To assess whether serine deprivation and rotenone treatment cause cell death, we measured cell viability using Sytox Green after exposing cells to these conditions for 72 hours. We find that there is indeed more cell death in cells cultured without serine at most concentrations of rotenone. However, cell death did not exceed 4% in any of the conditions tested, suggesting this is not a major contributor to the cell doubling phenotypes. These data are now presented in Supplementary Figure 1C of the revised manuscript. However, in light of Reviewer 2’s comments, along with a comment from Reviewer 3 about whether rotenone induces ROS and cellular stress responses, we have decided to remove the proliferation data involving rotenone that were in Figure 1F and 1G of the original manuscript. The rationale is that the potential confounding impacts of rotenone on viability make interpreting the proliferation data difficult. Instead, we have focused Figure 1 of the revised manuscript on the observation that there is specifically a correlation between the cell NAD+/NADH ratio and serine synthesis.

(2) The authors propose a model in which the deprivation of extracellular nutrients impacts mitochondrial respiration, which in turn increases the NAD+/NADH ratio and ultimately affects metabolic biosynthetic pathways that occur in the cytosol, such as serine biosynthesis. The mechanism by which nutrient availability is sensed and transmitted across different cellular compartments to regulate mitochondrial redox status remains unclear. This concern is particularly relevant for serine metabolism, as its synthesis occurs in the cytosol, but the authors connect it to mitochondrial respiration. Compartment-specific measurements of NAD+/NADH ratio would help to understand to what extent the redox state is affected by nutrients in the mitochondria and in the cytoplasm (see also minor critiques point 2). Moreover, the use of the genetic tool LbNox could be employed to manipulate the NAD+/NADH ratio in a compartmentspecific manner, while also avoiding the toxicity of certain compounds, such as rotenone. This set of experiments would add depth to the investigation, which might otherwise appear too descriptive.

(A) Compartment-specific measurements of NAD+/NADH ratio would help to understand to what extent the redox state is affected by nutrients in the mitochondria and in the cytoplasm

The question of how nutrient availability is sensed and transmitted across cellular compartments to impact mitochondrial respiration is important. However, rigorous assessment of compartment-specific metabolism is quite challenging, as we are not aware of tools to accurately measure redox ratios in a compartment-specific manner. Direct assessment of cofactor levels in subcellular compartments requires long isolation times and are unlikely to be accurate (PMID: 27565352). Rapid immunopurification of mitochondria has been used to estimate metabolite levels and ratios, but accurate measurements are hindered by rapid oxidation of NADH to NAD+. The use of fluorescence lifetime imaging (FLIM) to monitor NADH levels does not allow for accurate monitoring of the NAD+/NADH ratio as NAD+ cannot be visualized and NADH cannot be distinguished from NADPH. Additionally, the resolution of FLIM to interrogate compartment-specific signals is limited (PMID: 38594590). Fluorescent sensors, such as SoNar, have been used to image the NAD+/NADH ratio in compartments, though SoNar is sensitive to pH changes, which vary across compartments, and it has been argued that these sensors are more suitable for qualitative, not quantitative, changes in the NAD+/NADH ratio (PMIDs: 25955212, 29181426). It has also been argued that sensors are not amenable to measurement of mitochondrial ratios, as the predicted ratios are too reduced for the range of the sensors. Given these technical limitations, we opted to attempt a rapid subcellular fractionation (~25 second process to separate cytoplasm and mitochondria) followed by enzyme-based measurements of the NAD+/NADH ratio (PMID: 36883551), acknowledging the limitations of this approach. We find that across both A549 and H1299 cells, the mitochondrial NAD+/NADH ratio is lower than the cytosolic NAD+/NADH ratio, as expected. Using this approach, we find that in A549 cells, serine depletion leads to a decreased cytosolic NAD+/NADH ratio compared to serine-replete conditions while having no impact on the mitochondrial NAD+/NADH ratio. On the other hand, serine depletion leads to an elevated cytosolic NAD+/NADH ratio in H1299 cells while also having no impact on the mitochondrial NAD+/NADH ratio. In parallel, we used extracellular pyruvate exposure as a positive control, which should support cytosolic NAD+ regeneration, and rotenone as a negative control, which should suppress mitochondrial NAD+ regeneration. We show that pyruvate led to an elevated cytosolic NAD+/NADH ratio whereas rotenone treatment led to a decreased cytosolic NAD+/NADH ratio. Despite rotenone inhibiting complex I of the electron transport chain, we did not observe a change in the mitochondrial NAD+/NADH ratio (Author response image 2). This likely indicates that this assay is not sensitive enough to detect changes in mitochondrial NAD+/NADH, and we opted not to include these data in the revised manuscript given the limitations of the approach.

**Author response image 2.** Rapid subcellular fractionation to examine compartment-specific NAD+/NADH ratios. (A) Cytosolic and mitochondrial NAD+/NADH ratios of A549 cells grown with or without serine for 24 hours, n=3. (B) Cytosolic and mitochondrial NAD+/NADH ratios of H1299 cells grown with or without serine for 24 hours, n=3. (C) Cytosolic and mitochondrial NAD+/NADH ratios of H1299 cells treated with either 1 mM pyruvate or 50 nM rotenone for 24 hours, n=3. P-values were calculated using a Student’s t-test, *p<0.05, **p<0.01. Data shown are means ± SD.

We nevertheless draw the following conclusions from these data:

(1) Changes to mitochondrial NAD+/NADH either do not occur or are not captured with this approach. Even rotenone treatment, which inhibits complex I and might be expected to change mitochondrial redox state, does not change the measured mitochondrial NAD+/NADH ratio.

(2) The whole cell NAD+/NADH ratio most likely reflects changes in the cytosolic NAD+/NADH ratio. While observing no impact on the mitochondrial NAD+/NADH ratio after rotenone treatment, we still find the cytosolic NAD+/NADH ratio is decreased. Moreover, both pyruvate and serine depletion led to an elevated cytosolic NAD+/NADH ratio in H1299 cells, which we observe at the whole cell level.

(3) H1299 cells depleted of serine elevate the cytosolic NAD+/NADH ratio, while rotenone treatment decreased the cytosolic NAD+/NADH ratio despite changes in mitochondrial respiration. This suggests that redox shuttles, such as the malate aspartate shuttle, play a role in communicating changes in mitochondrial redox dynamics to the cytoplasm. We test this hypothesis as described in response to Reviewer 2, point B, below.

(B) The mechanism by which nutrient availability is sensed and transmitted across different cellular compartments to regulate mitochondrial redox status remains unclear

Multiple known shuttles are involved in exchanging redox equivalents between the mitochondria and the cytosol. It is likely that multiple shuttles are involved, or could be involved in the right context, but one major shuttle is the malate aspartate shuttle (MAS), and the MAS has been shown previously to support de novo serine synthesis (PMID: 37647199). Thus, we hypothesized that the MAS is involved in the response involving elevated mitochondrial respiration in H1299 cells to increase the whole cell NAD+/NADH ratio upon serine deprivation. To test this, we used CRISPR/Cas9 to generate H1299 cells lacking MAS components GOT1, MDH1, or GOT2 and measured the cell NAD+/NADH ratio. We did not knock out MDH2 given its integral role in the TCA cycle. We find that when MDH1 and GOT2 are knocked-out, H1299 cells no longer exhibit elevated whole cell NAD+/NADH ratios upon serine deprivation. Consistently, removing MDH1 and GOT2 also blunted the increase in oxygen consumption as well as the increase in serine synthesis upon serine deprivation. This suggests that MDH1 and GOT2 activity though the MAS support the process by which mitochondrial NAD+ regeneration is transmitted to the cytoplasm to support serine synthesis. We have added these data as Supplementary Figure 7 in the revised manuscript.

(C) Moreover, the use of the genetic tool LbNox could be employed to manipulate the NAD+/NADH ratio in a compartment-specific manner

We thank Reviewer 2 for the suggestion to consider whether *Lb*NOX might be used to manipulate the NAD+/NADH ratio in a compartment-specific manner. We expressed *Lb*NOX in both the cytoplasm and the mitochondria of A549 (serine non-responsive) cells. We predicted that if *Lb*NOX expression, either in the cytoplasm or the mitochondria, affected the NAD+/NADH ratio, proliferation in serine depleted conditions might be improved. However, we found that expressing *Lb*NOX in the cytoplasm or the mitochondria of A549 cells had no effect on the NAD+/NADH ratio. Thus, *Lb*NOX expression in either compartment also did not change proliferation in serine depleted conditions. These data are consistent with the known limitations of this genetic tool. While *Lb*NOX can increase NADH oxidation in response to some interventions like rotenone, it does not necessarily change the NAD+/NADH ratio of unperturbed cells. This was reported in the original description of *Lb*NOX (PMID: 27124460). We confirmed that *Lb*NOX was successfully expressed via immunoblotting, and also confirmed that *Lb*NOX functioned by showing either cytoplasmic or mitochondrial *Lb*NOX expression improves cell proliferation following complex I inhibition. Thus, expressing *Lb*NOX in A549 cells is not informative for understanding compartment specific metabolism following serine deprivation. Nevertheless, as this question is likely to come up for other readers, we have included these data as Supplementary Figure 6 in the revised manuscript.

**Reviewer #2 (Recommendations for the authors):**

Minor critiques:

(1) It seems clear from the authors' data that the response to serine depletion in terms of cell proliferation is not determined exclusively by PHGDH levels. It would be useful to measure the levels of the other two enzymes in the serine synthesis pathway and also to measure serine uptake under normal conditions in the different groups of cells. This information could provide some insight into the different responses of cancer cell lines to serine deprivation.

(A) It would be useful to measure the levels of the other two enzymes in the serine synthesis pathway

Reviewer 2 raises a fair point, and we agree that measuring levels of other enzymes in the serine synthesis pathway is informative. Thus, we measured the expression of phosphoserine aminotransferase 1 (PSAT1) and phosphoserine phosphatase (PSPH) across all cancer cells examined and find that, similar to PHGDH protein expression, PSAT1 and PSPH protein expression is lower in many cancer cells that are more sensitive to serine withdrawal (e.g. MCF7). However, among the cancer cells where PHGDH protein expression did not explain the response to serine withdrawal, the protein expression of PSAT1 and PSPH also did not explain how well the cells proliferate without environmental serine. These data have been included in Supplementary Figure 2B of the revised manuscript.

Of note, we measured serine synthesis enzyme expression for the six cancer cell lines whose proliferation in serine depleted conditions better correlated with a change in the NAD+/NADH ratio than it did with PHGDH expression: Calu6, 8988T, A549, MIA PaCa2, H1299, and HCT116. For these cells, we correlated proliferation upon serine depletion with PHGDH, PSAT1, and PSPH protein expression and found that interestingly, there was a significant negative correlation between PHGDH protein expression and proliferation upon serine deprivation. This was not observed for PSAT1 expression, and a statistically significant positive correlation between proliferation and PSPH protein expression was noted, though the variation in PSPH protein expression was large. We have added these correlation data to the revised manuscript as Supplementary Figure 2F.

(B) It would be useful to measure…serine uptake under normal conditions in the different groups of cells

Per the Reviewer’s request, we performed absolute quantification of serine uptake rates in serine-replete conditions for three serine “non-responder” cancer cells (Calu6, 8988T, A549) and three serine “responder” cancer cells (MIA PaCa-2, H1299, HCT116). We did not observe a notable difference in serine uptake rate and whether cells responded to serine deprivation. Additionally, with the exception of 8988T cells having a higher serine uptake rate than the other cells, there was no statistical difference in serine uptake across the cancer cells tested (Author response image 3).

**Author response image 3.** Basal serine uptake rate of exponentially growing cells in serine replete conditions. Serine levels were measured using GC MS before and after 24 hours of serine depletion and normalized by area under the growth curve (PMID: 26954548). P-values were calculated using one-way ANOVA followed by a post-hoc Tukey HSD test, *p<0.05, **p<0.01

(2) The authors experimentally demonstrated that some cancer cells respond to serine depletion with an increase in mitochondrial respiration, but the molecular mechanism behind this is not addressed. There is some evidence in the literature showing that serine acts as an activator of the glycolytic enzyme PKM, which is coherent with an increased mitochondrial respiration in the absence of serine (PMID: 23064226). The authors could discuss their findings in the context of this paper. Additionally, they could provide some insights about baseline mitochondrial activity in the different cell lines. Indeed, it seems that "redox responsive cells" might have an overall increased basal OCR.

We appreciate the suggestion that pyruvate kinase M (PKM) may mediate the elevation in mitochondrial respiration in response to serine depletion. Given that serine is an allosteric activator of PKM, and PKM suppression can increase mitochondrial OCR, we discuss this possibility in the Discussion section of the revised manuscript using the following text:

“Interestingly, serine is an allosteric activator of the glycolytic enzyme pyruvate kinase, which converts phosphoenolpyruvate to pyruvate and generates ATP (Chaneton, 2012). Thus, decreased environment serine availability in addition to differences in pyruvate kinase activity may yield lower glycolytic ATP, resulting in greater mitochondrial respiration in serine redox responder cancer cells.” (Lines 443-447)

Additionally, we appreciate the reviewer’s observation that redox responsive cells may have an overall increased basal respiration rate. We directly measured mitochondrial dependent oxygen consumption in the same assay to test whether redox responsive cells exhibit higher mitochondrial respiration. We find that while the redox responsive H1299 and MIA-PaCa2 cells have higher mitochondrial respiration than non-responsive cells, HCT116 cells that are also redox responsive to serine deprivation, did not exhibit higher mitochondrial respiration compared to redox non-responsive Calu6, 8988T, and A549 cells (Author response image 4). However, when comparing redox non-responders versus responders as a whole, there was a statistically significant difference in basal OCR. Together, this suggests that basal mitochondrial respiration rate in serine-replete conditions may be related in some cases to whether cancer cells elevate mitochondrial respiration and the NAD+/NADH ratio upon serine deprivation, but this cannot be the full explanation given the HCT116 cell data. We also acknowledge the reviewer’s statement that we do not understand the molecular mechanism by which respiration responds to serine deprivation and explicitly state this in the revised manuscript.

**Author response image 4.** Basal Oxygen consumption rate (OCR) of cancer cells in serine-replete conditions. (A) Kinetic OCR measurements of cancer cells before and after rotenone and anti-mycin injection, n=8. Data shown are means ± SD. (B) Quantified mitochondrial OCR (removing residual OCR), n=8. Values are averages obtained over three measurements. P-values were calculated via nested ANOVA, ****p<0.001

(3) There is a discrepancy between the basal values of the OCR from the same cell lines in different experiments, i.e., Figure 3A and Supp. Figure 3C, or in different experiments, Figure 3A, Figure 5E, and Figure 6A. The authors need to comment on/clarify that. Moreover, authors are encouraged to show ECAR values to support the conclusion that lactate production is not differentially affected by serine depletion, and thus, does not contribute to the increase in the NAD+/NADH ratio.

We recognize the differences in basal OCR values across different experiments. Given experiment-to-experiment variation and the need for different cartridges for each Seahorse experiment, we have found that measured OCR values using Seahorse assays vary across experiments despite the same conditions. Additionally, while we aim to seed the same number of cells per assay, cell seeding and cell quantification after each Seahorse assay can contribute to variation. Given this variability on a per-assay basis, we performed a singular experiment across all examined cancer cell lines considered to minimize variation in oxygen sensor calibration and address the reviewer question about whether absolute differences might contribute to response. These data are shown in Author response image 4.

Regarding the reviewer’s request to present ECAR data, we note that measuring ECAR is dependent on using unbuffered media and for this reason do not routinely measure ECAR. Our concern is that removing serum from the culture conditions can impact OCR measurements, and we instead prioritized maintaining the same media composition across all sets of experiments (i.e., cell proliferation assays, NAD+/NADH assays, kinetic tracing assays, and OCR measurements). Additionally, we point out that ECAR does not directly measure lactate. We refer the Reviewer to data included in the manuscript where GC-MS was used to directly measure lactate secretion over time for cells cultured with or without serine. These data are presented as Supplementary Figure 3B in the revised manuscript.

(4) There seems to be also a discrepancy between the levels of M+2 citrate and the fraction labelled (Figure 5C versus Supplementary Figure 6C) in the H1299 cell line upon serine depletion, whereby the M+2 fraction seems unexpectedly lower in serinedeprived cells. In those conditions, H1299 cells showed an increased mitochondrial respiration, which is consistent with increased total citrate levels. This could be explained by a faster TCA cycle activity and the presence of higher-order isotopologues of citrate upon serine starvation. Is this the case? Showing the abundance of the different citrate isotopologues and their contribution to the total pool would help to interpret the results.

We thank Reviewer 2 for this thoughtful comment regarding the discrepancy between M+2 citrate produced (normalized ion counts per cell) versus fraction of the total intracellular citrate pool that is M+2 labeled in serine depleted H1299 cells. In our kinetic U-^13^C-glucose tracing experiments, where we performed isotope labeling for up to 15 minutes, we only see a greater presence of M+3 citrate from fully labeled glucose without robust changes in M+4, M+5, or M+6 citrate (Author response image 5). An elevated M+3 citrate could represent pyruvate carboxylase activity, where M+3 labeled pyruvate is converted to M+3 oxaloacetate that then reacts with unlabeled acetyl-CoA to generate M+3 citrate.

We also find that the total citrate pool in H1299 cells is elevated upon serine depletion (see Supplementary Figure 6C in the original manuscript). Thus, the fractional contribution of an isotope to the citrate pool may decrease despite an increase in the amount of the particular isotope. In the original manuscript, we included data from kinetic U-^13^C-glutamine tracing in H1299 cells cultured with or without serine (Supplementary Figure 6I,J of the original manuscript). We find that H1299 cells depleted of serine exhibit greater M+4 citrate (via oxidative decarboxylation) and greater M+5 citrate (via reductive carboxylation) compared to serine-replete H1299 cells. Thus, one other potential explanation for why M+2 citrate from kinetic U-^13^C-glucose tracing represents a lower fraction of the total citrate pool in serine depleted H1299 cells is because there is a larger contribution from glutamine to the citrate pool. While there was no difference in the fraction of the citrate pool that consists of M+4 citrate, there was a greater fraction of the citrate pool labeled by M+5 citrate upon kinetic U-^13^C-glutamine tracing in serine depleted H1299 cells (see Author response image 6A, B). There was also a greater fraction of the citrate pool from M+6 citrate upon kinetic U-^3^C-glutamine tracing in serine depleted H1299 cells (Author response image 6C). This would require M+3 pyruvate labeling from glutamine, which may be due to malic enzyme, which converts M+4 malate to M+3 pyruvate. M+3 pyruvate may also be formed by PEPCK, which could convert M+4 oxaloacetate to M+3 phosphoenolpyruvate, leading to M+3 pyruvate. While understanding the source of M+6 citrate from glutamine is out of the scope of this study, it may highlight an interesting metabolic shift in H1299 cells depleted of serine that could elevate the total intracellular citrate pool.

**Author response image 5.** Citrate isotopologues. (A. M+3; B. M+4; C. M+5; D. M+6) from kinetic U-^13^C-glucose tracing in H1299 cells depleted of serine for 24 hours. For all measurements, citrate values were normalized to internal norvaline standard and cell number for each condition, n=3. Data shown are means ± SD.

**Author response image 6.** Fraction of the citrate pool labeled by U-^13^C-glutamine in H1299 cells depleted of serine for 24 hours. (A) Fraction of the total citrate pool that is M+4 citrate (formed via oxidative decarboxylation), n=3. (B) Fraction of the total citrate pool that is M+5 citrate (formed via reductive carboxylation), n=3. (C) Fraction of the total citrate pool that is M+6 citrate, n=3. Data shown are means ± SD.

(5) The lipid depletion part of the paper seems to be somewhat tangential. The effect of lipid depletion on the NAD+/NADH ratio in A549 cells is modest, and the effects of dual serine and lipid depletion on OCR and NAD+/NADH ratio are not consistent. Moreover, if the authors want to show that these different nutritional environments affect lipid synthesis, apart from glucose incorporation into citrate, they would need to show actual carbon incorporation into palmitate, probably at longer time points.

We apologize for the lack of clarity for how mitochondrial respiration and the NAD+/NADH ratio play a role in governing glucose oxidation to citrate. To better highlight our logic and rationale for investigating alterations in NAD+/NADH homeostasis and citrate synthesis under lipid depletion, we have added the following text to the revised manuscript:

“Oxidative biosynthetic reactions other than serine synthesis can also be constrained by the NAD+/NADH ratio. For example, cancer cells deprived of environmental lipids increase oxidative citrate production, and we have previously found that citrate synthesis, either through glucose oxidation or glutamine oxidation, is limited by NAD+ availability (Li, 2022) (Figure 5A, Supplementary Figure 8A). Thus, we sought to uncover whether the increase in the cell NAD+/NADH ratio by mitochondrial respiration in response to serine withdrawal specifically supports greater serine synthesis or also leads to greater oxidative citrate production.” (Lines 307-313)

While we have previously shown that alterations to the NAD+/NADH ratio can modify both citrate production and palmitate synthesis under lipid depleted conditions (PMID: 35739397), we agree with Reviewer 2 that no conclusion can be made about lipid synthesis without direct measurements and have revised the manuscript accordingly.

(6) In Figure 6C-6F, showing the results of the controls (+serine +lipids) will help to clarify the extent to which serine and citrate synthesis rates are affected by the different interventions.

We thank the reviewer for the comment. Because we specifically asked how dual serine and lipid starvation impacted either serine or citrate synthesis compared to singular nutrient deprivation alone, we performed the experiments focusing on these conditions. We felt that conducting an experiment that specifically targeted our question would be make the findings more accessible as we had compared the +serine +lipid conditions to either serine or lipid depletion alone earlier in our manuscript (Figure 2D and Figure 5G,H of the revised manuscript).

**Reviewer #3 (Public review):**

Summary:

The manuscript by Chang and colleagues provides new insights into how cancer cells adapt their metabolism under nutrient-deprived conditions. They find cells respond differentially to serine and lipid deprivation via oxidising the cell redox state, which enables biomass synthesis and cell proliferation. They identified mitochondrial respiration as the major mechanism that dictates the endogenous NAD+/NADH ratio. By incorporating a dual stress paradigm, serine and lipid deprivation, the study further suggests that the NAD+/NADH ratio can serve as a link to orchestrate the complex interplay between multiple nutrient changes in the tumour microenvironment.

Strengths:

A novel aspect of this study is the idea that cancer cells are not uniformly passive victims of nutrient limitation; some can actively invoke endogenous NAD+ regeneration to combat nutrient stress. The conclusion is well-supported by comparing multiple cell lines from different tissues and genetic backgrounds, which improves generalizability. While most of the smaller conclusions align with common reasoning and expectations, the step-by-step deduction that leads to a novel 'big picture' is commendable. Another notable strength is the integration of dual stress (lipid and serine deprivation), which better mimics the complex tumor microenvironment with multiple nutrient fluctuations, raising the translational potential of these findings. The observation that lipid-deprived cells can stimulate serine synthesis and support proliferation in a subset of cancer cell lines offers a novel perspective on metabolic plasticity under starvation conditions.

We thank Reviewer 3 for their time and for their comments to help us improve the manuscript. We also thank them for highlighting the strengths and significance of our findings.

Weaknesses:

(1) Although the authors derive a novel and valuable overarching concept, the presentation of this "big picture" is not clearly articulated, making it less accessible to readers outside the immediate field. It would greatly enhance the manuscript to include a clearer summary of the overarching model and its implications. Additionally, discussing the potential clinical significance and applications of the findings would increase the relevance and broader impact of the work. Finally, the manuscript's clarity and credibility are undermined by inconsistent figure labeling and the lack of statistical analysis, particularly for the Western blot data.

(A) It would greatly enhance the manuscript to include a clearer summary of the overarching model and its implications. Additionally, discussing the potential clinical significance and applications of the findings would increase the relevance and broader impact of the work.

We appreciate Reviewer 3’s suggestion to help clarify the findings of this study. To better articulate our overarching model, we have added the following text to the end of the Results section of the revised manuscript

“Taken together, we propose a model where environmental nutrient availability can impact mitochondrial respiration based on the specific cancer. Because mitochondrial respiration is a major pathway that regenerates NAD^+^, changes to mitochondrial respiration can alter the cell NAD+/NADH ratio, influencing the activity of major NAD^+^-requiring metabolic reactions such as serine synthesis and citrate synthesis that can be important for proliferation. We further propose that changes to the cell NAD+/NADH ratio can impact all oxidative biosynthetic reactions if the enzyme machinery is present, but that specificity for how the cell NAD+/NADH ratio changes is dependent on both cell-intrinsic factors and cellextrinsic factors (Figure 7)." (Lines 396-404)

Additionally, a new model figure was added as Figure 7 in the revised manuscript, which may help with understanding for a general audience.

To better highlight the potential clinical significance of these findings, we have added the following at the end of the Discussion section of the revised manuscript:

“Better understanding the mechanisms cells use to alter respiration and adjust the NAD+/NADH ratio in response to available nutrients could inform the complex interplay between cell-intrinsic and cell-extrinsic factors that determine cancer metabolic dependencies. This is particularly important to consider when targeting metabolism for cancer treatment. Many newer therapies targeting metabolism have not been successful in part because of metabolic plasticity to nutrient shifts (Amoedo, 2017; Fendt, 2020; Xiao, 2023). Co-targeting mitochondrial function limits metabolic adaptations and may also help predict the tissue nutrient conditions that result in pathway dependencies for specific cancers. Thus, better understanding how the cell NAD+/NADH ratio responds to nutrient levels in different cancers could improve selection of patients for cancer therapies that impact metabolism.” (Lines 483-492)

(B) “…the manuscript's clarity and credibility are undermined by inconsistent figure labeling and the lack of statistical analysis, particularly for the Western blot data.”

We apologize to the reviewer for any inconsistency in data presentation. To address the comment related to inconsistent figure labeling, we ensured all figures in the revised manuscript are labeled to allow readers to recognize what cell lines are used, what conditions are tested, what parameters are measured, and how the data may or may not be normalized. To address the reviewer’s comments about lack of statistical analysis, in the revised manuscript we ensured that statistical analyses are included for data presented in each figure, when appropriate. We also include a section titled “Statistics and Reproducibility” in the Methods section. In our revised manuscript, we have ensured that the p-value threshold is consistent throughout all figures, and have removed “ns” across the manuscript for consistency as suggested by Reviewer 3 in their minor comments. We also removed any explicit p-values included in figures where the p-values were close to reaching the threshold for significance (a=0.05). We have also performed additional statistical analyses where needed, including adding the pvalues for linear regression analyses, and ensured new data added to the revised manuscript also included appropriate statistical analyses.

For western blot data, we show representative immunoblots. However, we measured PHGDH, PSAT1, and PSPH protein expression in three biological replicates across examined cancer cells and quantified the average serine synthesis protein expression from each replicate performed with error bars that denote standard deviation (see Author response image 7). We performed a nested ANOVA to examine whether there was a statistically significant difference in PHGDH, PSAT1, and PSPH protein expression between non-responder and responder cancer cells. Interestingly, as noted in our response to Reviewer 2, we find a significant negative association between PHGDH protein expression and response to serine deprivation among the six cancer cells where PHGDH protein expression did not explain proliferation upon serine depletion.

**Author response image 7.** Serine synthesis enzyme protein expression in serine-replete and serine depleted cancer cells. (A) Immunoblots examining the expression of PHGDH, PSAT1, and PSPH in cancer cells as shown. HSP90 was used as a loading control. Data are from two separate biological replicates. (B) Mean levels of PHGDH, PSAT1, and PSPH normalized to loading control HSP90 across cancer cells from three separate biological replicates. Yellow denotes cancer cells that do not elevate mitochondrial respiration in response to serine depletion (non-responders). Blue denotes cancer cells that do elevate mitochondrial respiration in response to serine depletion (responders). P-values were calculated with nested ANOVA comparing non-responders and responders, **p<0.01

(2) While this study identifies changes in serine synthesis, mitochondrial respiration, PHGDH protein levels, and NAD+/NADH ratio in different cell lines, some of these relationships appear correlative rather than causally established (Figure 2; Figure 5; Figure 6). Some claims are thus overinterpreted. For example, the co-occurrence of increased NAD+/NADH ratio and citrate levels under lipid deprivation in A549 cells does not establish causality (Figure 5). Direct perturbation experiments that manipulate NAD+/NADH and assess downstream effects on citrate synthesis would substantially strengthen the conclusions.

We agree with Reviewer 3 that corresponding changes in proliferation, mitochondrial respiration, and serine synthesis are correlated to the NAD+/NADH ratio. As shown in Figure 4, we perturbed the NAD+/NADH ratio with FCCP and rotenone to measure downstream effects on serine synthesis. We also agree with the reviewer that doing similar experiments in the lipid depletion condition would highlight the relationship between the NAD+/NADH ratio and citrate synthesis. However, we point out that these experiments were already published in a manuscript from our group specifically showing that the NAD+/NADH ratio is limiting for citrate synthesis (PMID: 35739397). In that manuscript, the NAD+/NADH ratio was perturbed using electron transport chain inhibitors, including complex I inhibitors, which decreases the cell NAD+/NADH ratio. Exogenous electron acceptors were used to rescue the NAD+/NADH ratio, and under those conditions, cell proliferation, the NAD+/NADH ratio, and glucose and glutamine oxidation to citrate were measured with and without lipid depletion. We showed that decreasing the NAD+/NADH ratio decreases citrate synthesis through both glucose and glutamine oxidation and also affects palmitate synthesis. We could rescue citrate and palmitate synthesis by supplementing cells with exogenous electron acceptors. We also show that expressing cytosolic or mitochondrial NADH oxidase (*Lb*NOX; PMID: 27124460) in mitochondrial complex I-inhibited cells rescues proliferation in lipid depleted conditions and that *Lb*NOX expression raises oxidative citrate production at baseline. Given the extensive prior work showing the relationship between the NAD+/NADH ratio, oxidative citrate synthesis, and palmitate synthesis, efforts to repeat these same experiments for this manuscript were not warranted. We do show in the current manuscript that treating cells with AKB or FCCP, which raises the NAD+/NADH ratio, also increases glucose oxidation to citrate (Figure 5D of the original and revised manuscripts). We did this to confirm that the elevated M+2 citrate production from glucose in serine starved H1299 cells was related to an increase in the NAD+/NADH ratio as opposed to a specific response to serine depletion.

The study focuses predominantly on mitochondrial respiration as a source of NAD+ regeneration. However, it will also be interesting to check other significant pathways, such as NAD+ salvage, which have been implicated in supporting serine biosynthesis. In addition, the subcellular distribution of NAD+ may distinguish whether some cells are truly redox-unresponsive. Mitochondrial NAD+ regeneration might counteract the cytosolic NAD+ consumption, rendering a relatively stable intracellular NAD+/NADH ratio. The malate-aspartate shuttle can be an interesting aspect.

(A) The role of NAD+ salvage and serine biosynthesis

Per the reviewer’s request, we investigated whether NAD+ salvage might be involved in supporting serine synthesis. Specifically, the reviewer comments highlight an interesting question about whether NAD+ salvage may differentially contribute to serine synthesis between cancer cells that elevate mitochondrial respiration in response to serine depletion and cancer cells that do not change mitochondrial respiration in response to serine depletion. Specifically, we wondered whether cancer cells that do not elevate mitochondrial respiration in response to serine depletion depend more on NAD+ salvage to support proliferation in serine depleted conditions. To test this, we treated A549 and H1299 cells in serine depleted conditions with increasing doses of the nicotinamide phosphoribosyltransferase (NAMPT) inhibitor FK866. However, we found no statistically significant difference in sensitivity to FK866 upon serine depletion in these cells based on ANCOVA analysis (p=0.9332). Interestingly, we observe that A549 cells are more sensitive to FK866 treatment than H1299 cells in serine-replete media conditions (ANCOVA analysis, p=0.0004). This suggests that A549 cells at baseline may have greater dependence on NAD+ salvage compared to H1299 cells, though this is not specific to the response to serine depletion. We then asked whether nicotinamide mononucleotide (NMN), the product of NAMPT and the immediate precursor to NAD+ in the salvage pathway, would rescue the proliferation of A549 cells cultured without serine. We find that adding 100 µM NMN, a concentration that can impact PHGDHdriven serine synthesis (PMID: 30157431), does not change proliferation of A549 cells cultured without serine, unlike supplementing cells with AKB or FCCP, which increase NADH oxidation to NAD+. Together, these data suggest that NAD+ salvage does not play a major role in differentiating the redox response to serine deprivation between responder and non-responder cells. We have added these data as Supplementary Figure 3C,D of the revised manuscript.

(B) The role of the malate-aspartate shuttle and serine biosynthesis

The MAS has been shown to play an important role in serine synthesis (PMID: 37647199) and may facilitate elevation in mitochondrial respiration in response to serine depletion. As stated in response to Reviewer 2, measuring subcellular compartmentspecific NAD+/NADH ratios accurately is not feasible, so we utilized a functional approach to interrogate the role of compartmentalization. Specifically, we tested a role for the malate-aspartate shuttle (MAS). Using CRISPR/Cas9, we generated GOT1, MDH1, and GOT2 deleted H1299 cells. We did not knock out MDH2 given its integral role in the TCA cycle. Using the knockout lines, we measured the whole cell NAD+/NADH ratio and found that MDH1 and GOT2 KO cells no longer exhibited an elevated cell NAD+/NADH ratio upon serine depletion compared to non-targeting controls (NTC). Consistently, MDH1 and GOT2 KO cells did not elevate OCR upon serine deprivation, nor did they exhibit greater serine synthesis rates compared to NTC cells. This suggests that MDH1 and GOT2 activity support the process by which mitochondrial NAD+ regeneration provides cytosolic NAD+ to support serine synthesis. We next asked whether MAS protein expression differed between cells that elevate respiration in response to serine depletion and cells that do not. While enzyme expression is not equivalent to activity, we wondered whether MAS protein expression would be lower in cells that do not increase their mitochondrial respiration upon serine depletion. However, we observed no major difference in GOT1, GOT2, MDH1, or MDH2 protein expression across the cancer cells examined (Author response image 8). Further experimentation is needed to measure MAS activity across lines and may reveal a mechanism by which mitochondrial respiration is governed by nutrient availability, such as levels of environmental serine.

**Author response image 8.** Protein expression of the malate aspartate shuttle enzymes GOT1, MDH1, GOT2, and MDH2 in cancer cells cultured without serine for 24 hours. Membranes were first probed for GOT1 or GOT2 then stripped and re-probed for MDH1 or MDH2.

(3) The authors should acknowledge the limitations of short-term isotope tracing in their experimental design. Differences in metabolic rates across cell lines can affect the kinetics of metabolite labeling, limiting the direct comparability of metabolic fluxes between them. As a result, observed changes may reflect transient adaptations rather than stable metabolic reprogramming. It is important to clarify that the study primarily captures short-term responses, and the conclusions may not extrapolate to longer-term adaptations or protein-level changes under sustained nutrient stress.

We thank the reviewer for this comment. We apologize for any confusion around experimental approaches. We agree that in the case of acute changes in nutrient availability at the start of kinetic isotope tracing, the observed changes may reflect transient adaptations. However, cells are exposed to conditions for 24 hours prior to performing kinetic tracing. This approach allows us to examine changes that occurred in response to the nutrient condition, not acute changes. Additionally, we add fresh, prewarmed treatment media at least two hours prior to commencing kinetic isotope tracing. Upon analysis of kinetic isotope tracing, we examine whether cells were at metabolic steady state by monitoring metabolite levels over the course of tracing. For example, in the kinetic glucose tracing experiments in serine depleted cells, total serine levels are relatively stable throughout the experiment, and we find that total serine levels are greater in H1299 cells after 24 hours of serine starvation. Data showing total metabolite pools over the course of tracing are shown in the Supplementary Figures (for example, see Supplementary Figure 8C-H in the revised manuscript). The period of treatment prior to the start of kinetic isotope tracing is described in the figure legends and further detailed in the “Kinetic U-^13^C-Glucose Isotope Tracing Experiments” section of the Methods in the revised manuscript. To improve clarity, we added a kinetic graph showing total serine levels over time in Supplementary Figure 2I of the revised manuscript as this can address whether synthesis rates are captured while cells are at metabolic steady state. We also discuss these considerations better in the revised manuscript with the following text:

“Importantly, we confirmed kinetic U-^13^C-glucose tracing was performed at metabolic steady state by ensuring metabolite levels were stable at each collected time point (Supplementary Figure 2I)” (Lines 178-180).

**Reviewer #3 (Recommendations for the authors):**

It is important to note that, in many cases, the data show only trends rather than statistically significant differences, or, if significance testing was performed, the results are not clearly labeled. For example, in Figure 1B, no p value was denoted in the figure, and the scale bar is quite high, precluding the conclusion that "AKB and rotenone dosedependently increased and decreased the cell NAD+/NADH ratio". In Figure 2E, no pvalue was shown to support the result that "H1299 cells had higher serine level than A549 cells". Inconsistencies in how significance is denoted across figures (e.g., asterisks vs. numerical values; "ns" vs. no label) make interpretation difficult. Marginal significance (e.g., p = 0.06 in Figure B) can be reported explicitly, but all figures should clearly denote whether comparisons are significant or not. Conclusions drawn from nonsignificant trends should be appropriately stated.

We thank Reviewer 3 for this important comment and for highlighting specific instances where the manuscript could be improved. Please see response to Reviewer 3, Major Comment 1B. We also agree with Reviewer 3 that it is integral to ensure that conclusions made from non-significant trends are appropriately stated. For example, we explicitly mention that there was no statistically significant difference between the serine synthesis rate of A549 cells depleted of serine versus A549 cells depleted of both serine and lipids (Line 375). As another example, we changed the phrase “Moreover lipid depletion led to a greater fraction of total serine derived from glucose in serine depleted A549 cells” to “Moreover, lipid depletion appeared to lead to a greater fraction…” (Line 376).

Western blot data supporting PHGDH expression variability across cell lines (e.g., Supplementary Figure 2B, 3E) appear to rely on single experiments. At least three biological replicates are required to substantiate claims about discordance between PHGDH levels and serine sensitivity. Supplementary Figure 4G presents overexpression validation based on a single Western blot without quantification. Including statistical validation from biological replicates would strengthen this point.

We thank Reviewer 3 for this suggestion. Western blots were repeated 3 times, although data from a representative blot is shown. Please see response to Reviewer 3, Major Comment 1B.

Certain data visualizations (e.g., Figure 2C) lack annotation indicating which data points correspond to which cell lines, limiting interpretability. All figures should include clear labels, consistent statistical notation, and complete legends. The author uses different color labels (redox-responsive (blue) and unresponsive (yellow) cell lines), which provides mechanistic clarity; however, this classification was not consistently used across the manuscript (e.g., Figures 2d and 2e). To further improve reader comprehension, consider adding conceptual schematic diagrams before each main result section to illustrate experimental logic, and a final diagram summarizing the proposed mechanism.

We apologize for any unclear data presentation. In the revised manuscript we have added greater clarity around what cell lines are used in each experiment and have added explicit labeling to specify cancer cell lines in Figure 2C of the revised manuscript. Throughout, we have ensured that any serine redox non-responder cell lines are labeled in yellow while serine redox non-responder cell lines are labeled in blue. We have also ensured that any lipid redox responder cells are labeled in green while lipid redox non-responder cells are labeled in dark purple, a change from the original manuscript. Finally, we have also added a schematic to summarize the proposed model in Figure 7 of the revised manuscript.

Although the authors provide justification for using H1299 and A549 as representative cell lines to study serine depletion, it remains unclear whether these two lines are equally suitable for investigating lipid depletion. Additional rationale or supporting data would help clarify their appropriateness for the lipid-related experiments.

We thank Reviewer 3 for this suggestion. We opted to study H1299 and A549 cells under lipid deprivation to assess their responses in relation to the response to serine deprivation. We specifically wanted to know whether these findings related to serine deprivation applied to other nutrient depleted conditions. We clarify this logic in the revised manuscript by adding the following text:

“Oxidative biosynthetic reactions other than serine synthesis can also be constrained by the NAD+/NADH ratio. For example, cancer cells deprived of environmental lipids increase oxidative citrate production, and we have previously found that citrate synthesis, either through glucose oxidation or glutamine oxidation, is limited by NAD+ availability (Li, 2022) (Figure 5A, Supplementary Figure 8A). Thus, we sought to uncover whether the increase in the cell NAD+/NADH ratio by mitochondrial respiration in response to serine withdrawal specifically supports greater serine synthesis or also leads to greater oxidative citrate production.” (Lines 307-313)

We have also included more detailed justification for focusing our studies on A549 and H1299 to study serine depletion by adding the following statements to the manuscript:

“We performed focused comparisons between A549 and H1299 cells because they exhibit differences in proliferation upon serine deprivation that are not explained by PHGDH protein expression, demonstrate differing responses of the cell NAD+/NADH ratio upon serine deprivation, and have similar basal proliferation rates.” (Lines 171-175)

The concentration of serine in replete media should be explicitly stated and justified. If the intention is to mimic physiological conditions, alignment with human plasma levels would increase translational relevance.

We agree that explicitly stating the concentration of serine in replete media is important. In the revised manuscript, we explicitly state that DMEM contains 400 uM of serine and that we use this concentration for serine-replete conditions (Line 102). While an important application of our manuscript is to better explain metabolic changes that can occur in physiologic conditions, we acknowledge that we did not test levels found in different tissues. Rather, by examining extreme conditions of high and low serine, we hoped to dissect how cells adapt to nutrient conditions, and testing the more subtle responses based on tissue serine levels will require a dedicated study.

Rotenone may elevate ROS levels and trigger cellular stress responses, potentially confounding proliferation assays. The authors should validate that concentrations used do not induce cytotoxicity or excessive oxidative stress, and ideally measure ROS levels to support interpretation.

We thank Reviewer 3 for raising this important point. We explicitly measured cell viability with the doses of rotenone used in this manuscript in cells cultured with or without serine. We find that rotenone dose-dependently increases cytotoxicity in A549 cells grown in serine-replete conditions in a statistically significant manner as calculated by simple linear regression. However, the cytotoxicity from rotenone is low (at most 4% in serine depleted conditions) and does not explain differences to rotenone sensitivity with respect to serine synthesis. These data have been added to Supplementary Figure 1C of the revised manuscript.

Evidence for lipid depletion can enhance serine synthesis in A549 cells is inadequate, for the marginal difference in NAD+/NADH ratio and slight increase of M+3 serine levels. The statement "any perturbation that increases the NAD+/NADH ratio led to both elevated serine and citrate production, regardless of what nutrient was depleted from the environment" (introduction section) should be reworded.

We thank Reviewer 3 for this suggestion. We have changed the above statement to the following:

“Lastly, we find that any perturbation that increases the NAD+/NADH ratio, including lipid deprivation, could paradoxically improve the proliferation of cells in serine depleted conditions.” (Lines 90-92).
